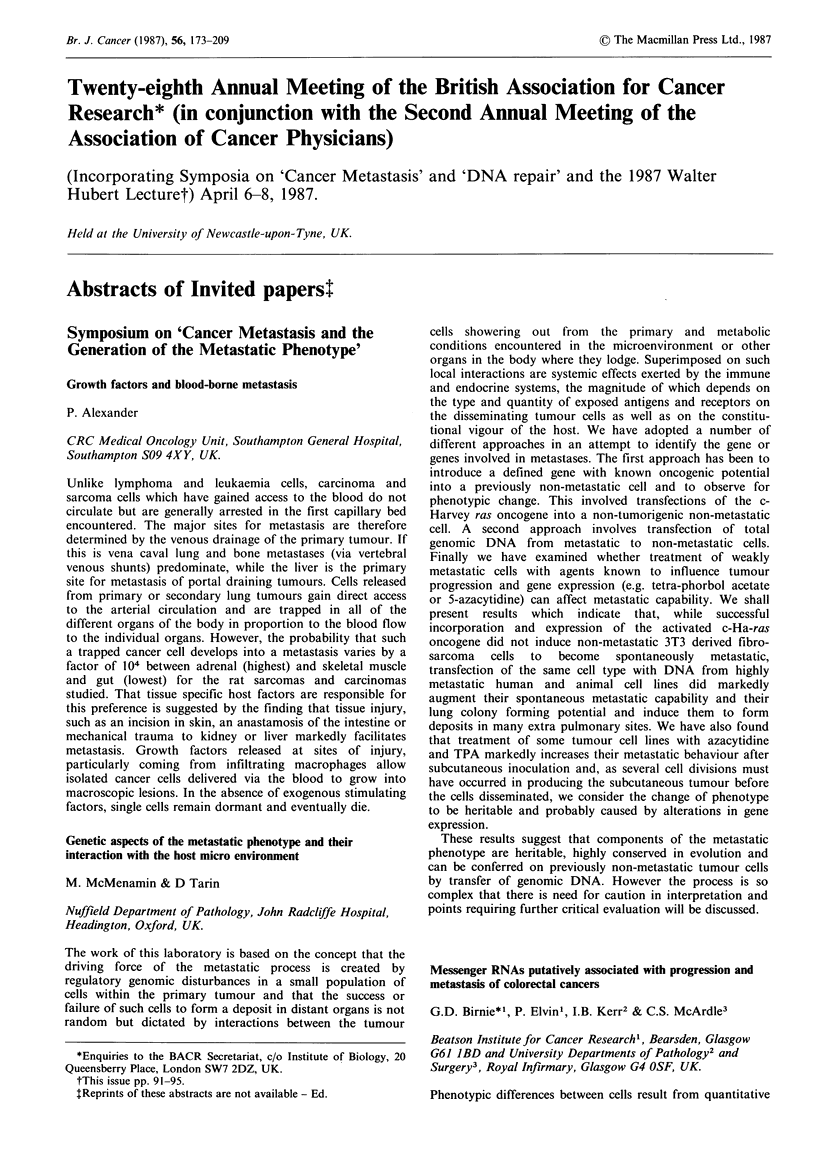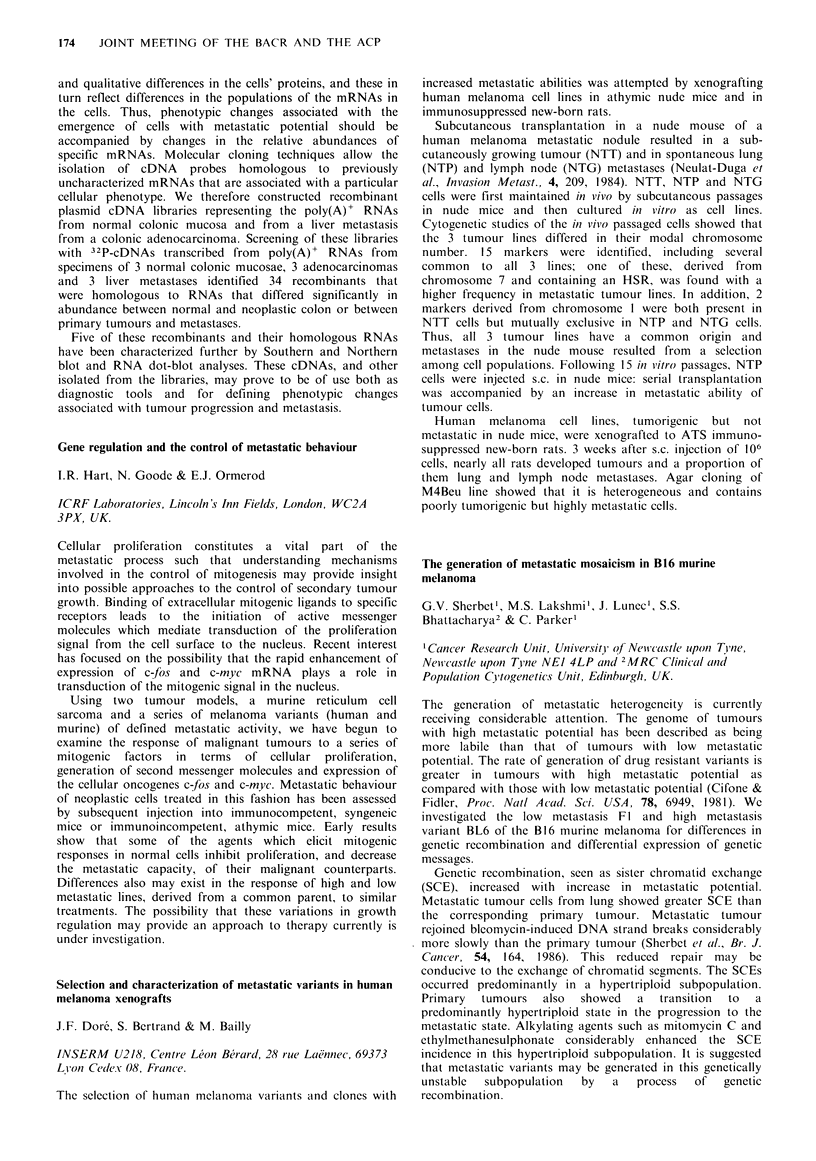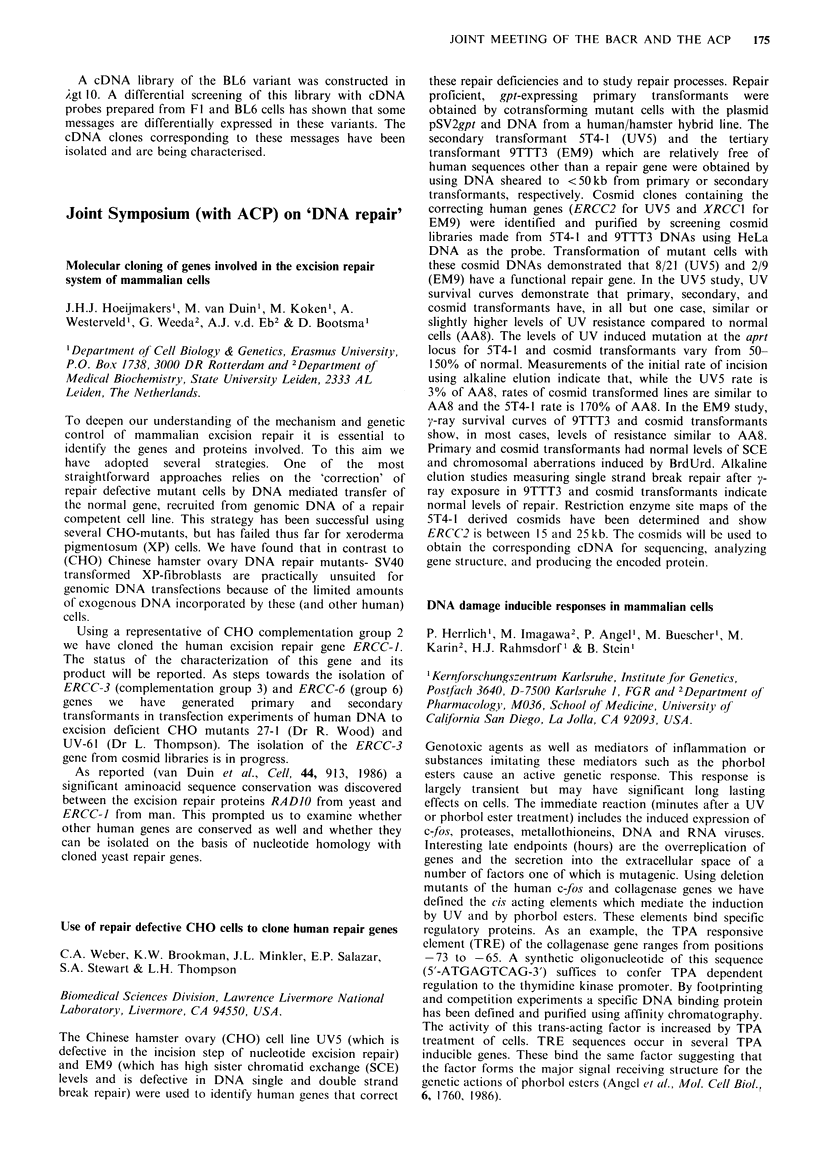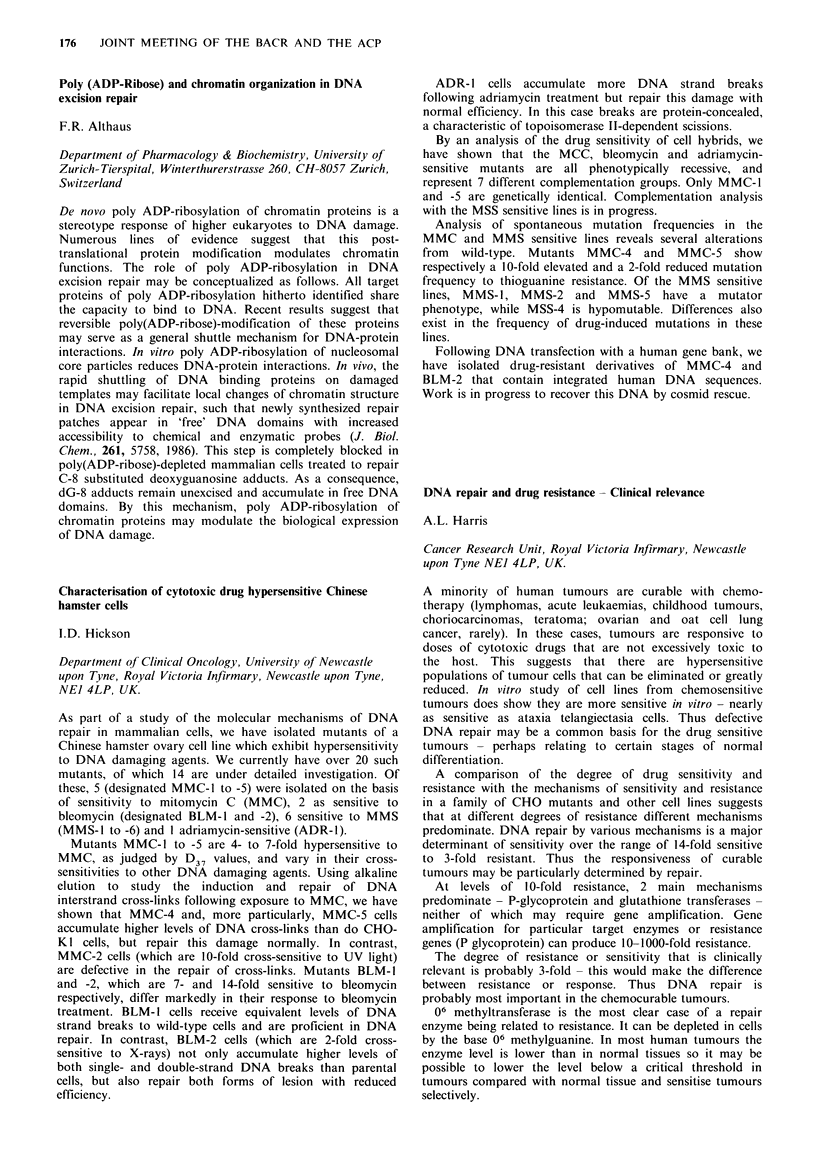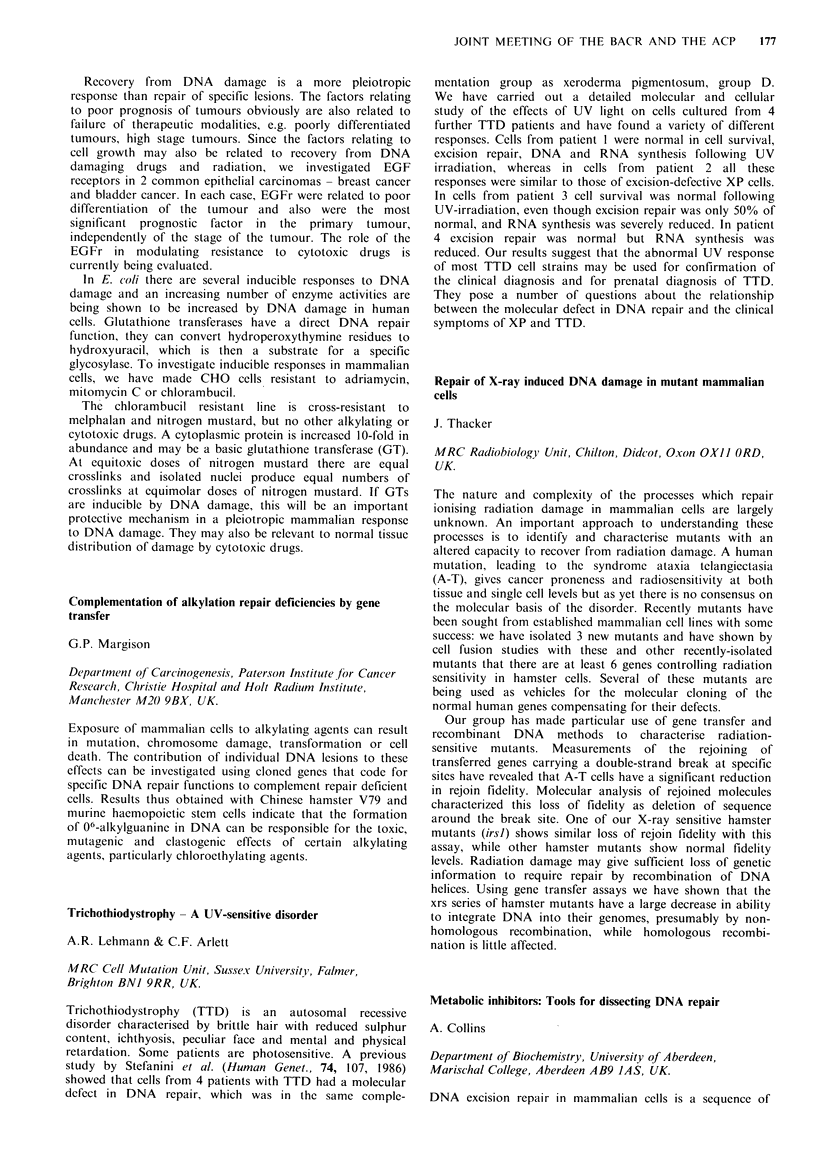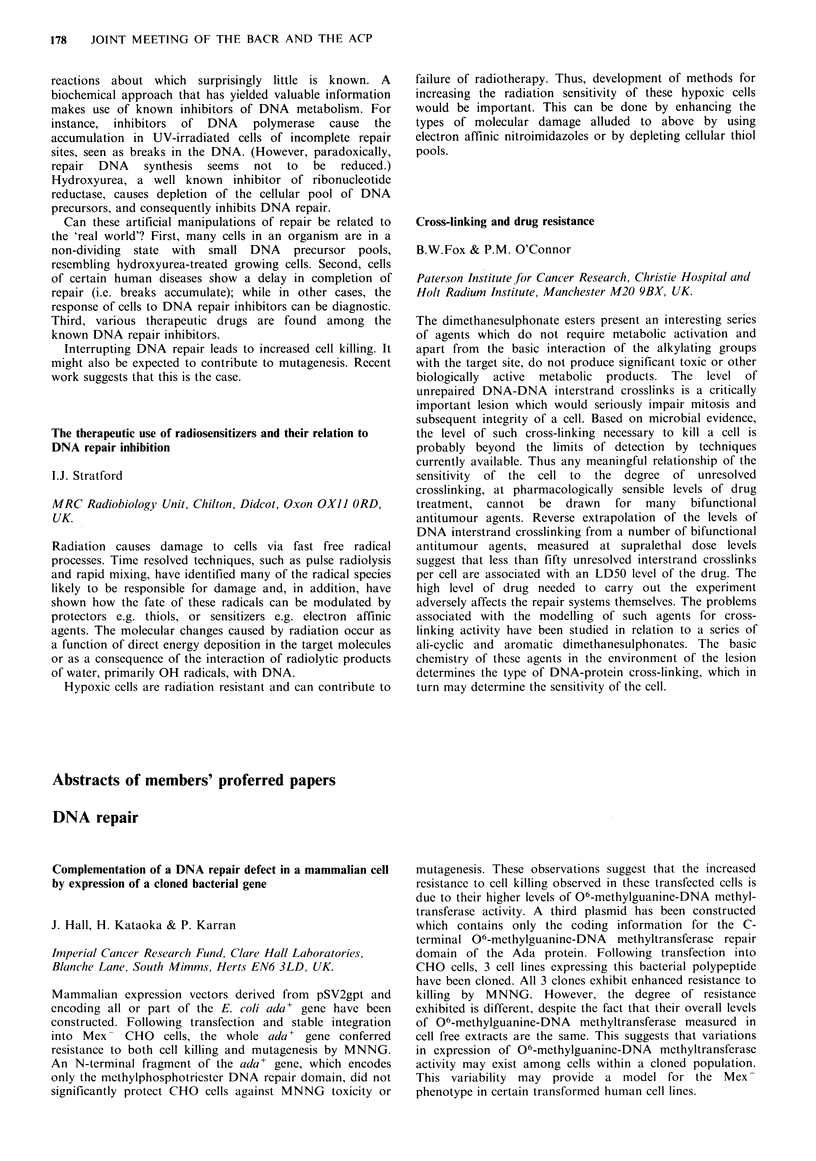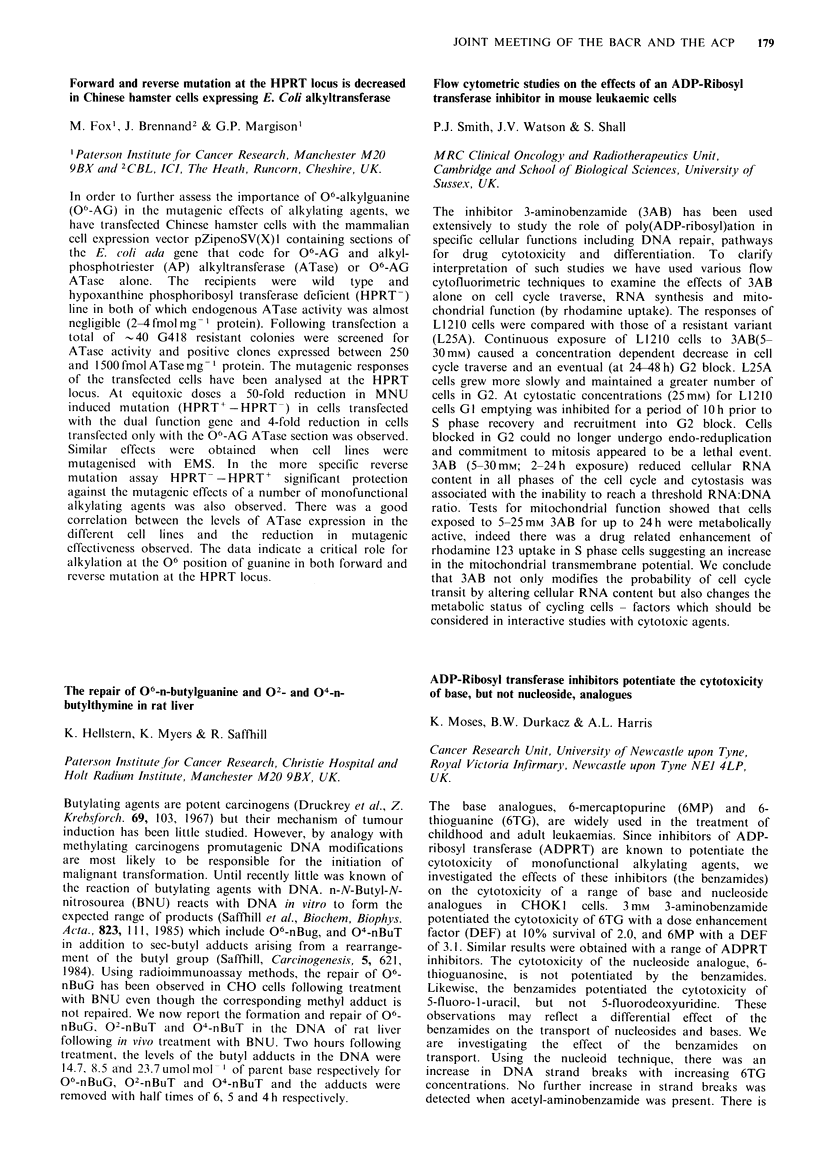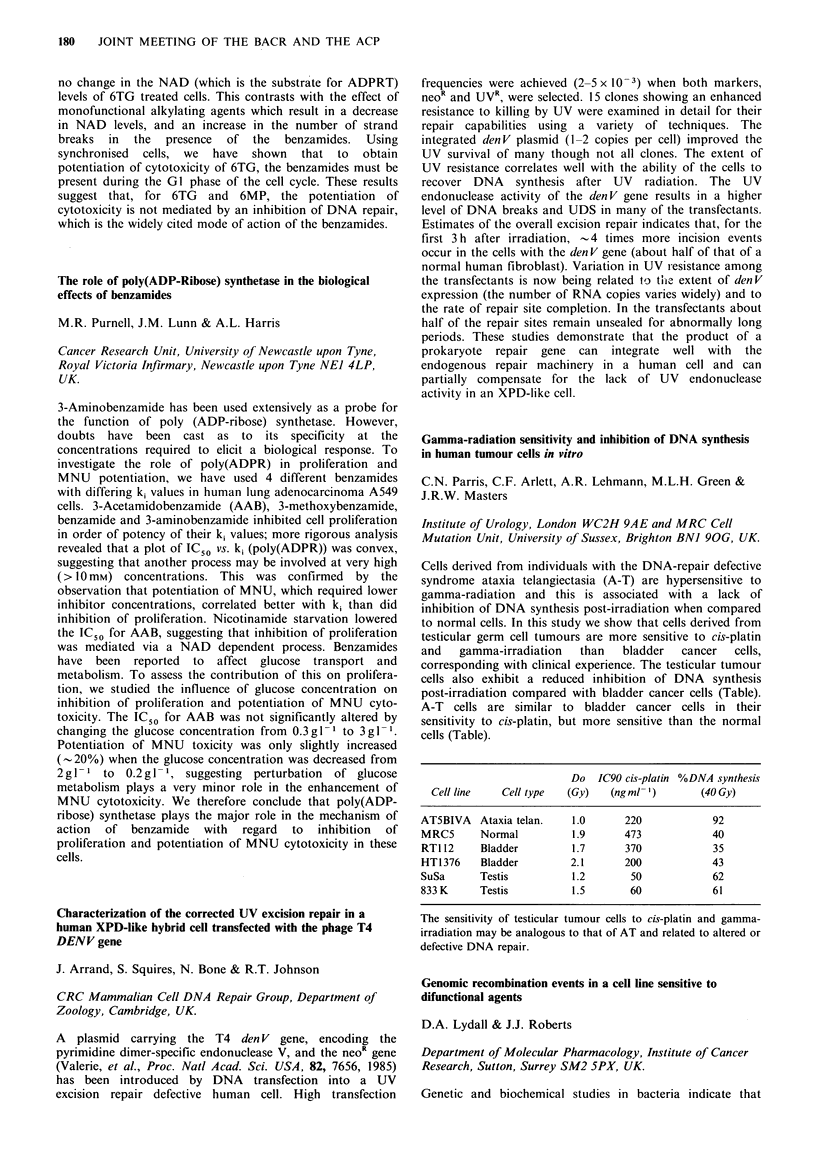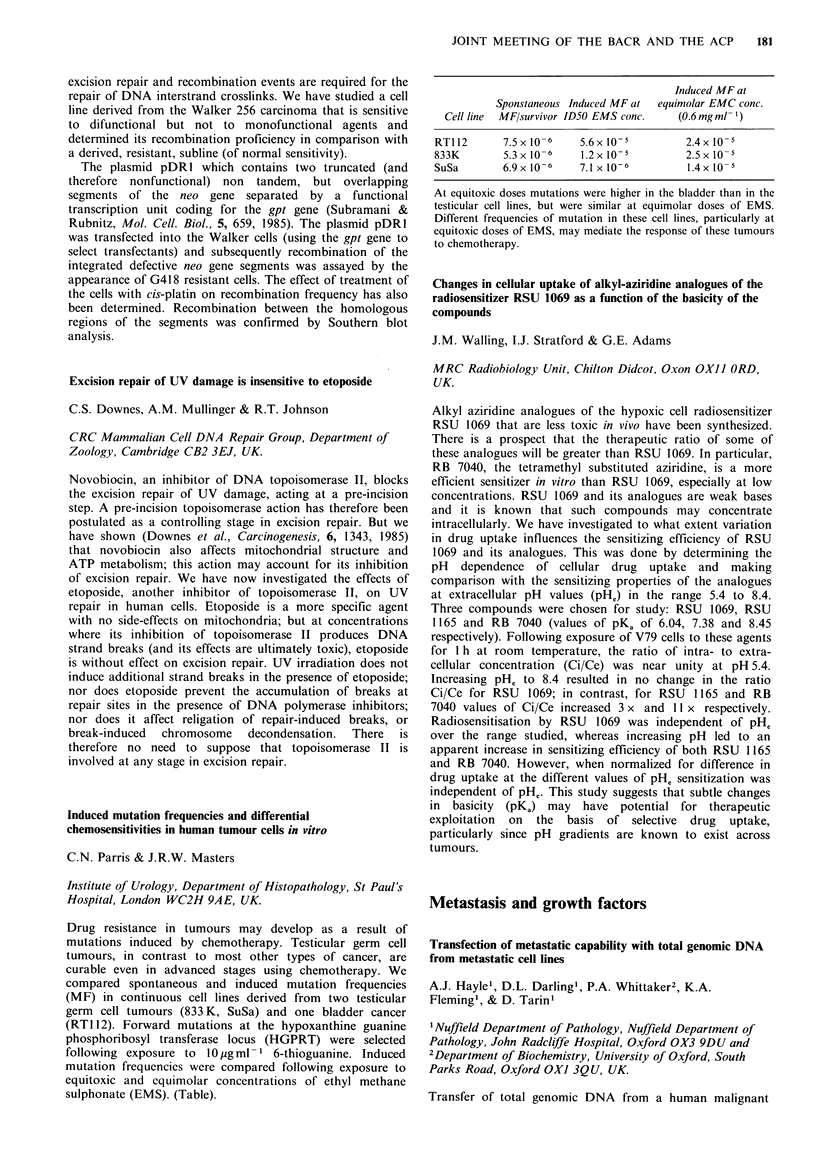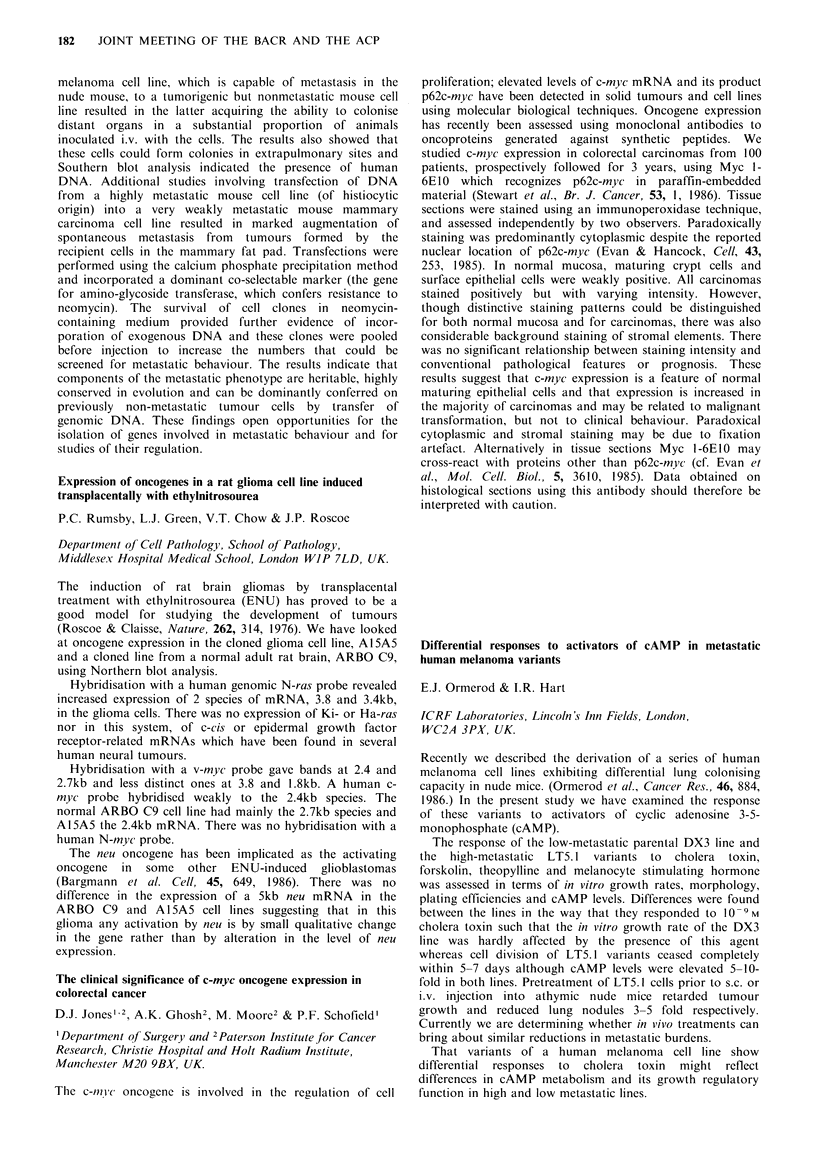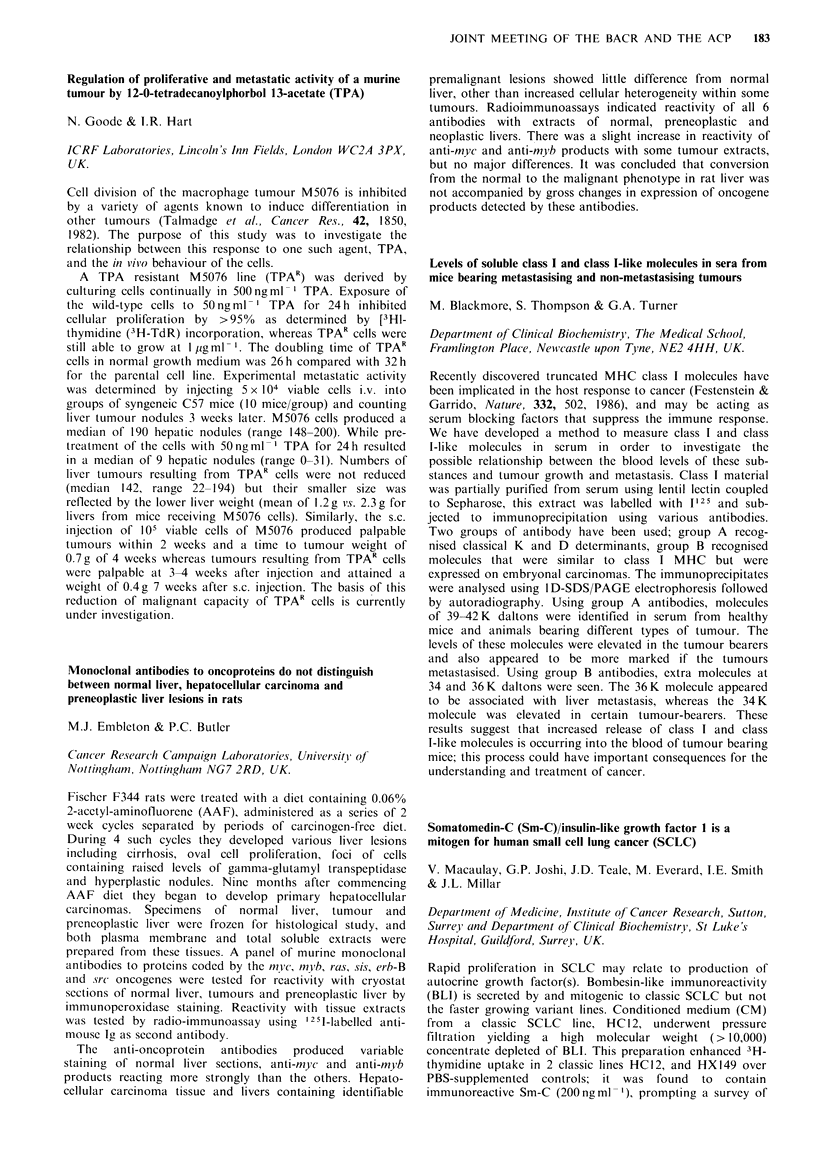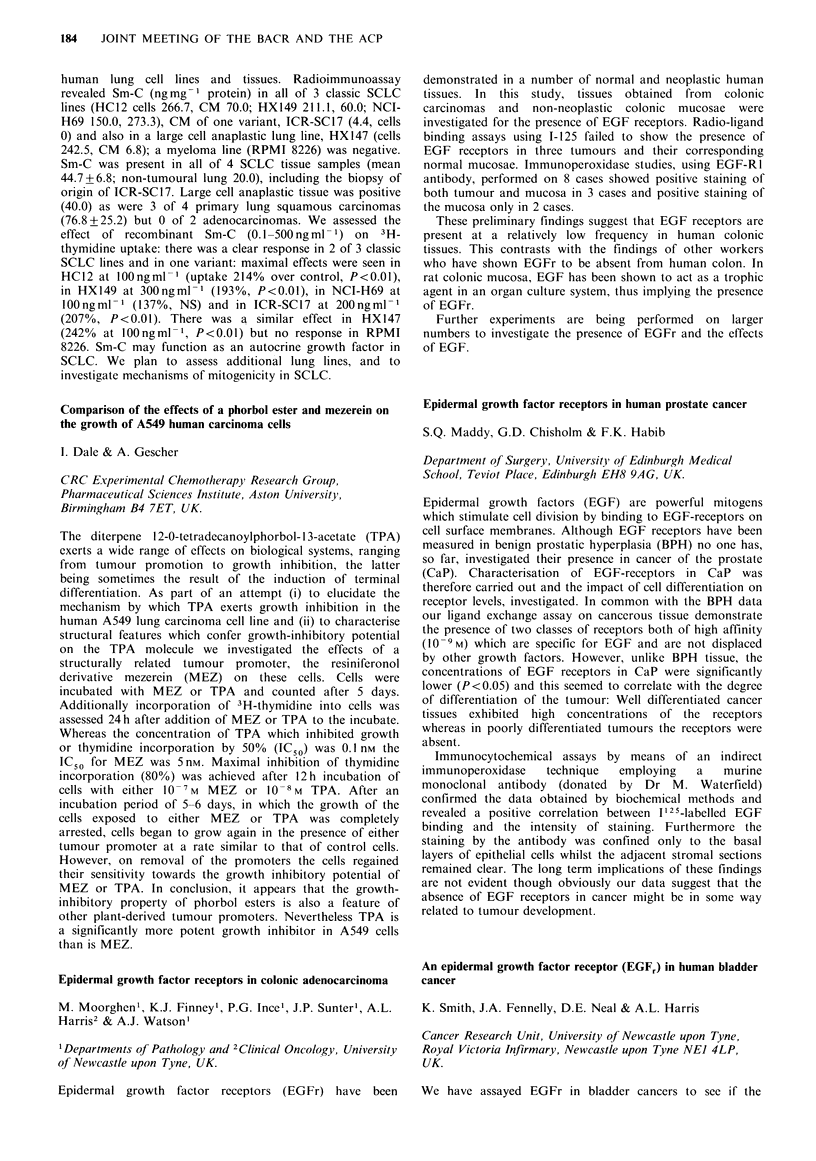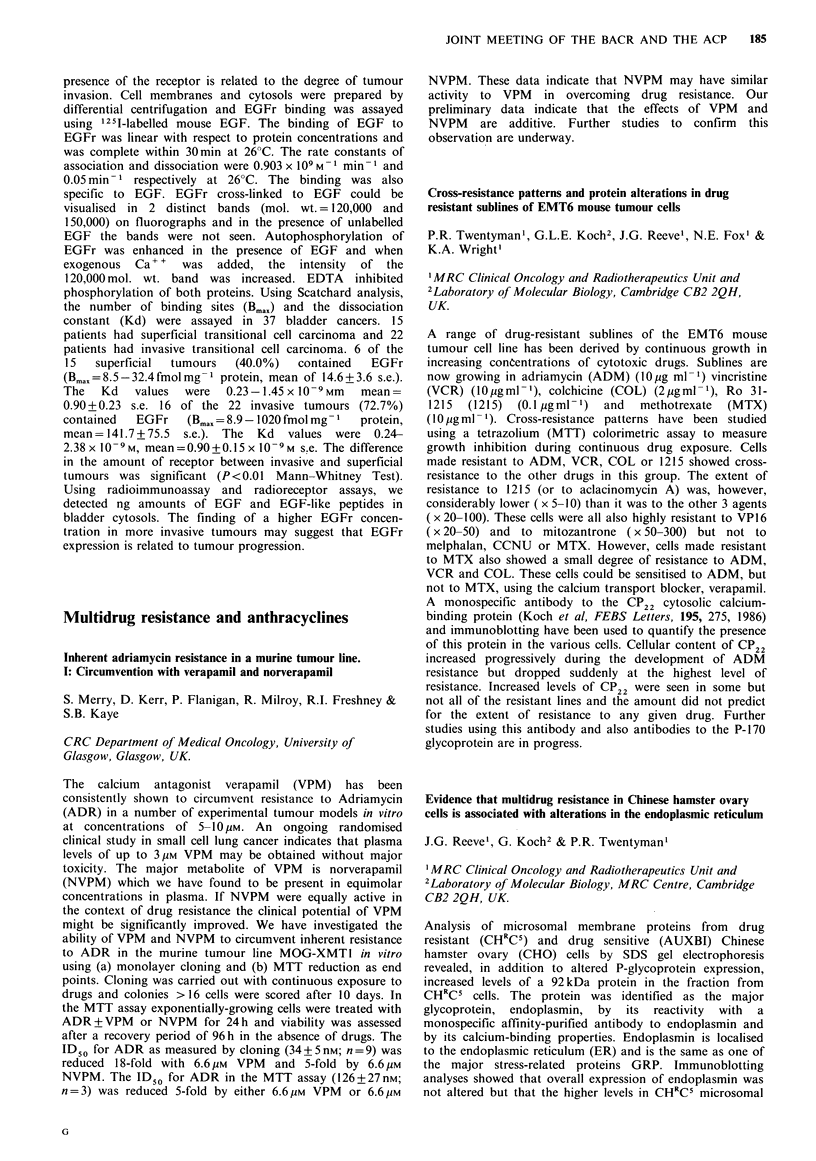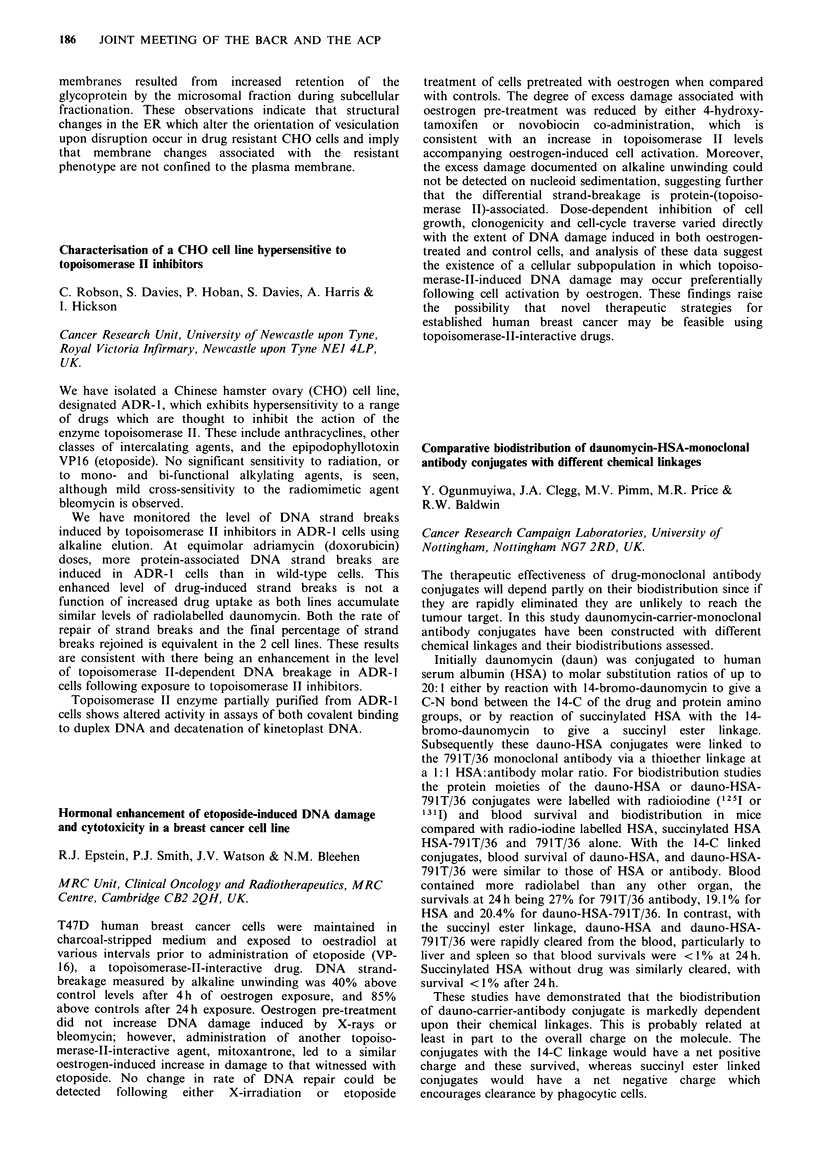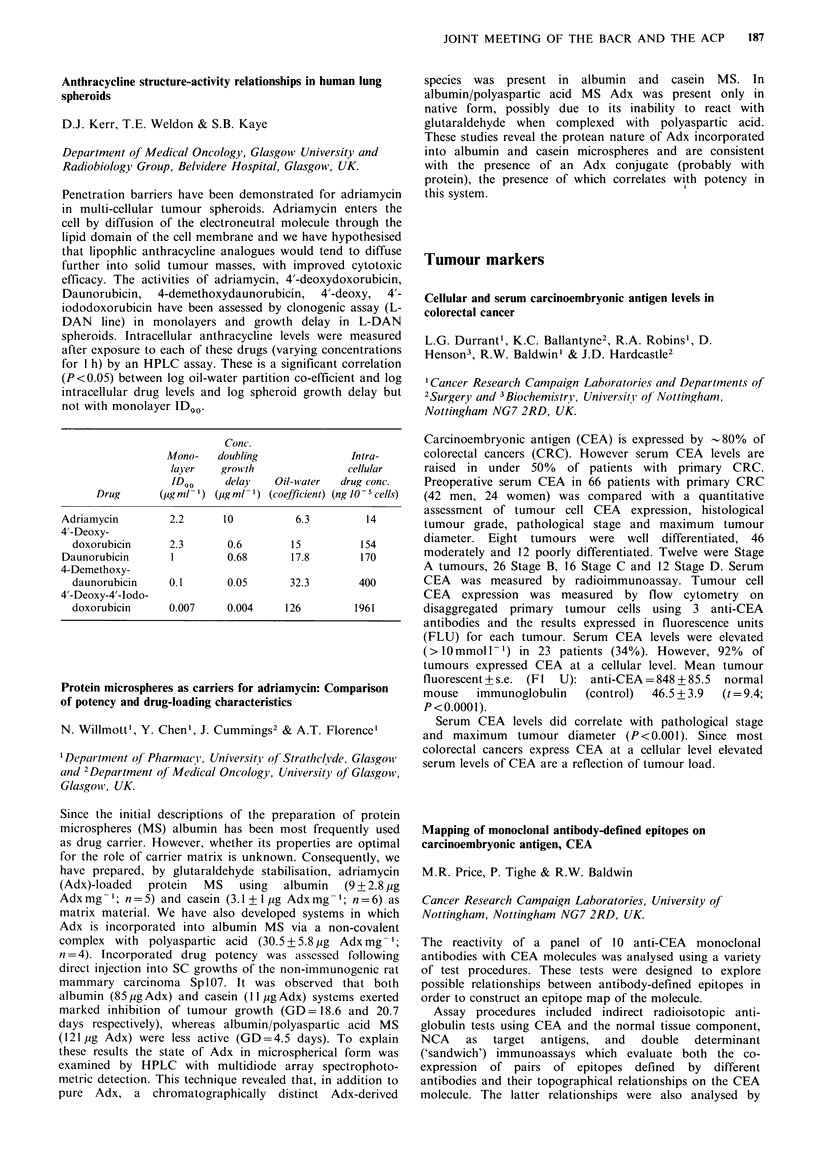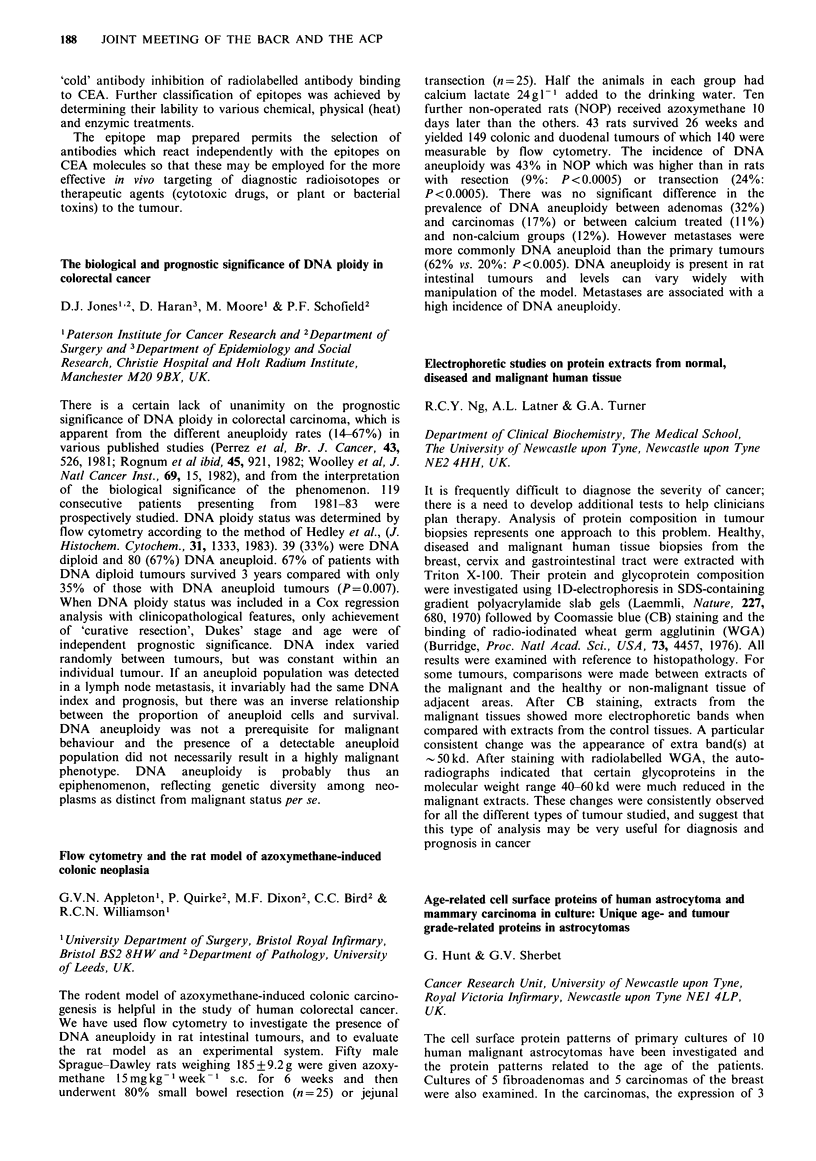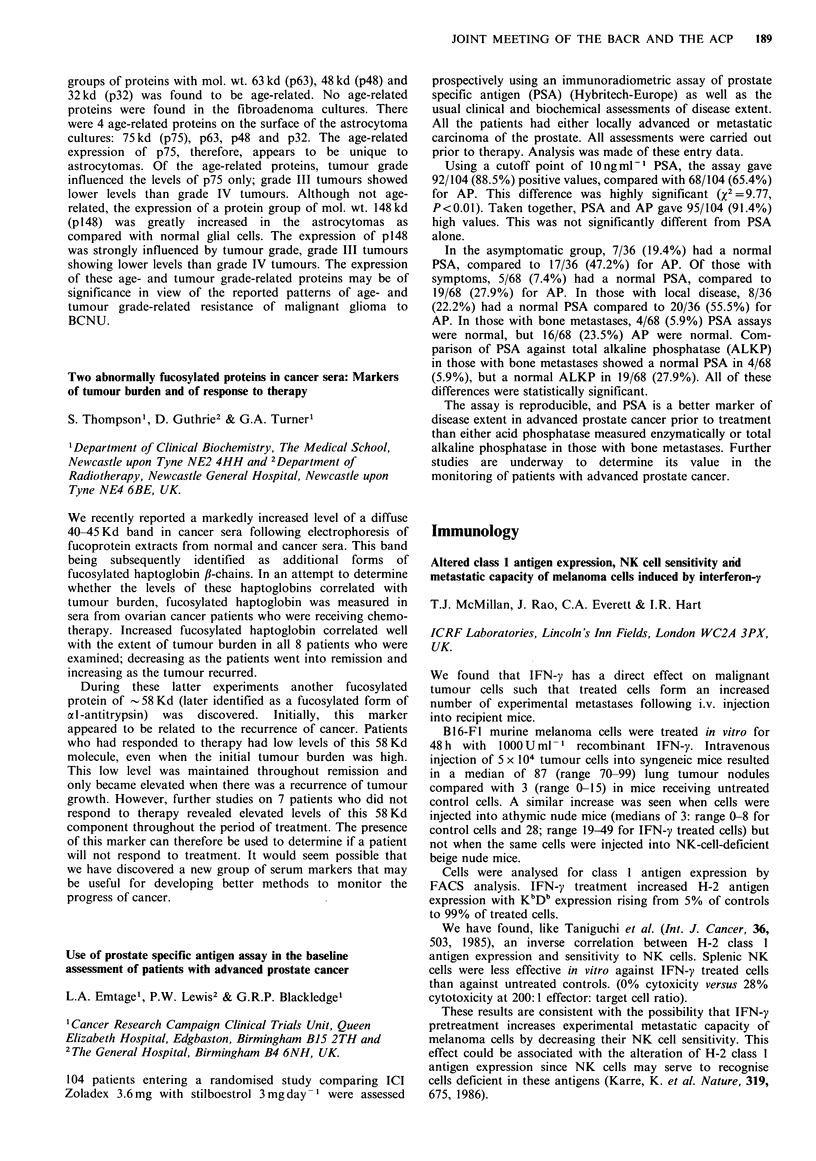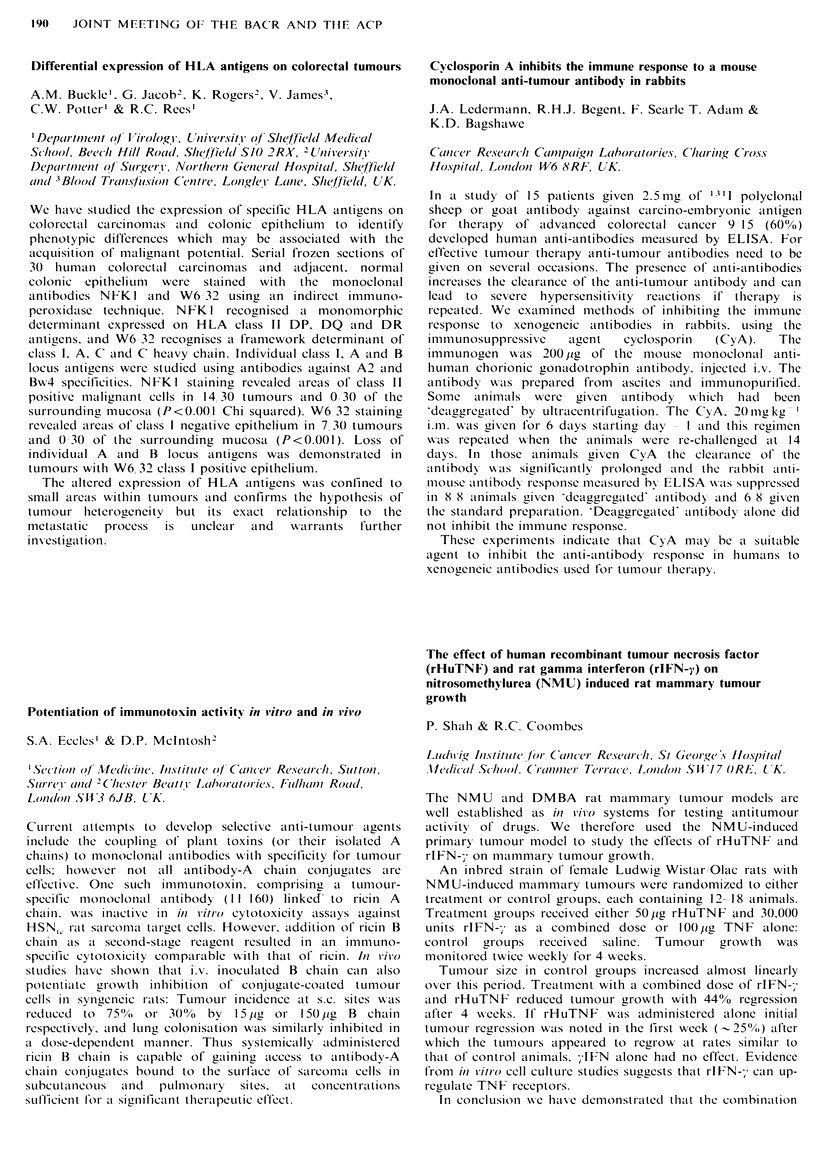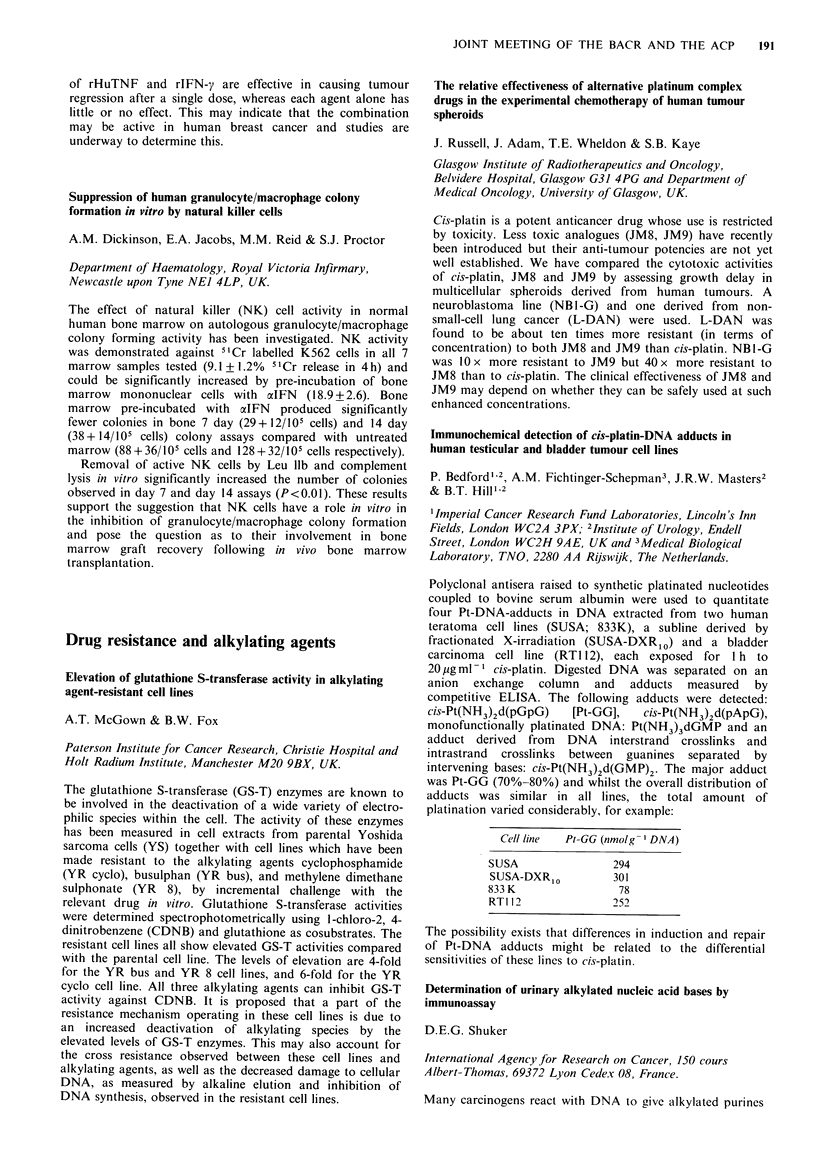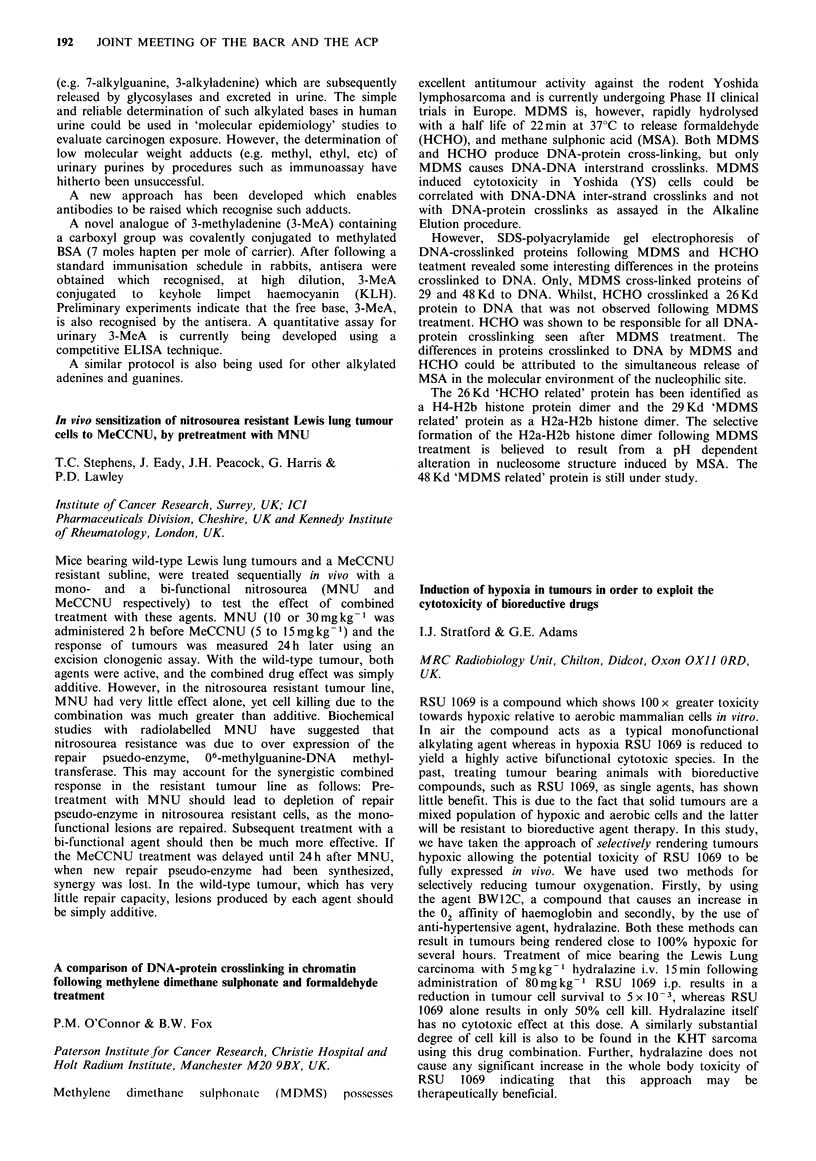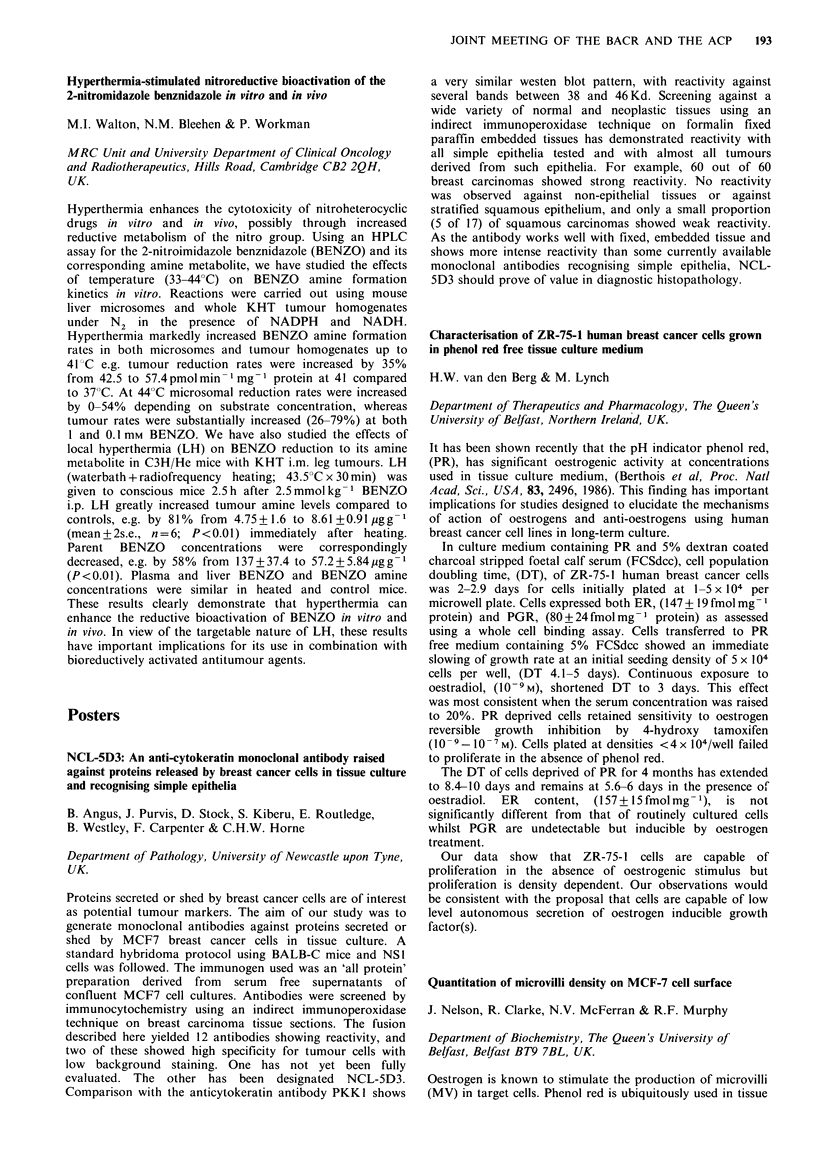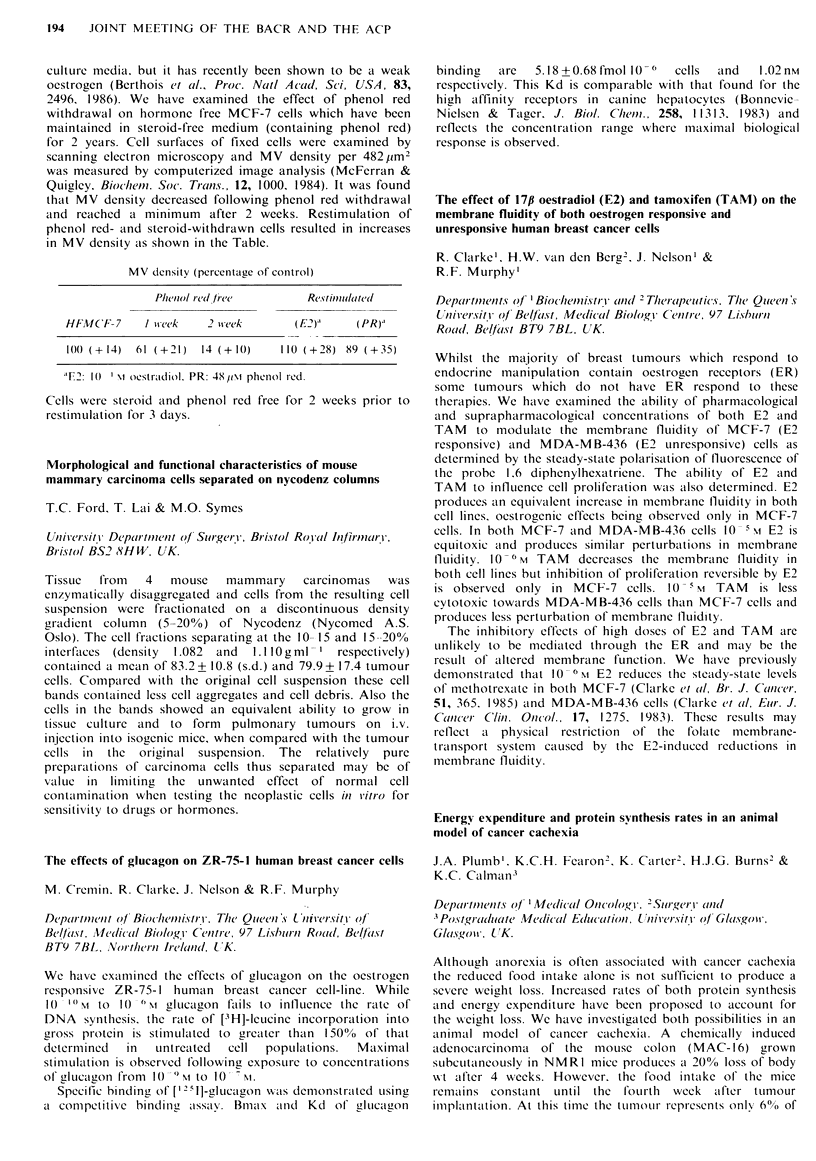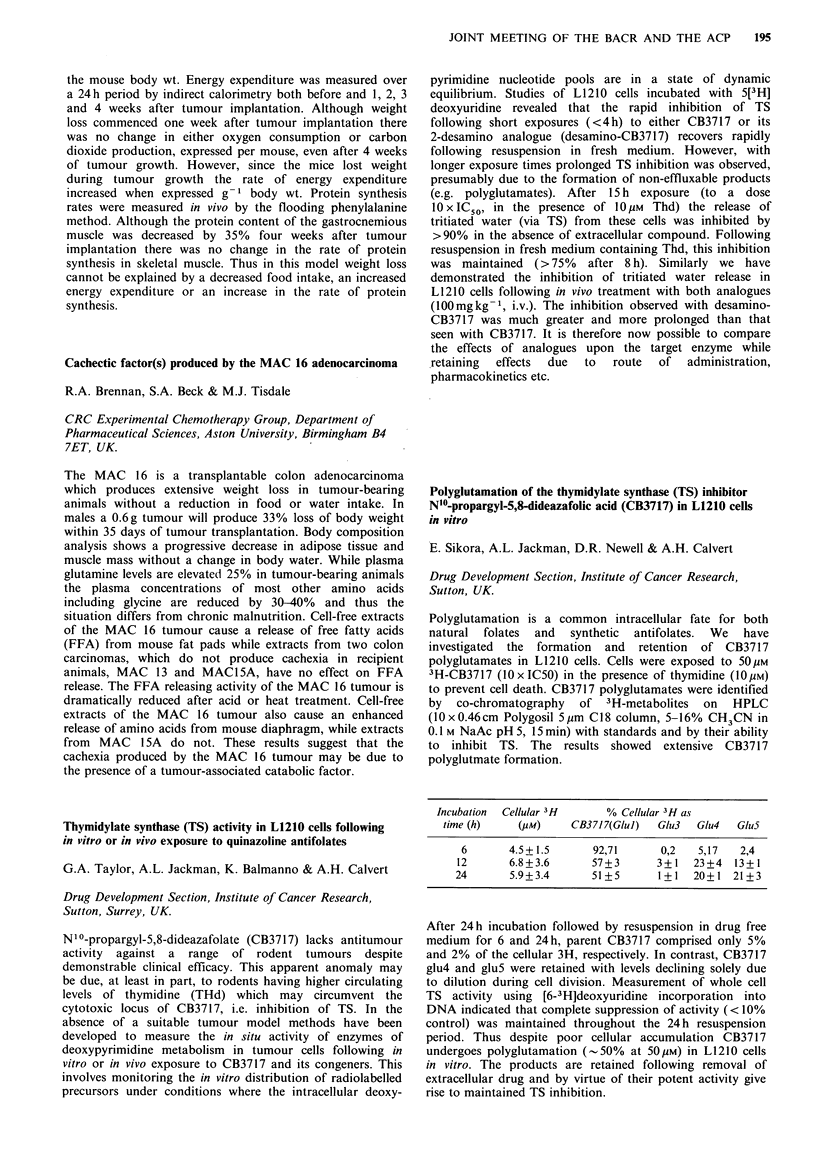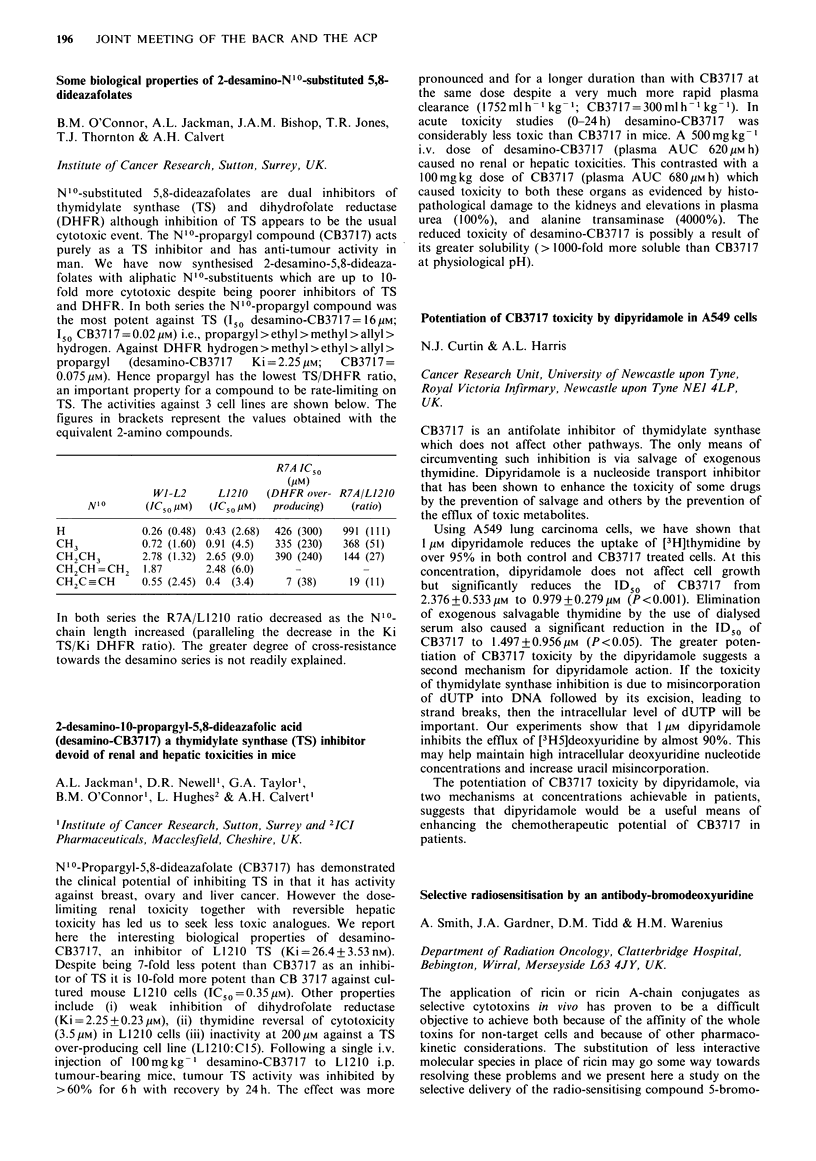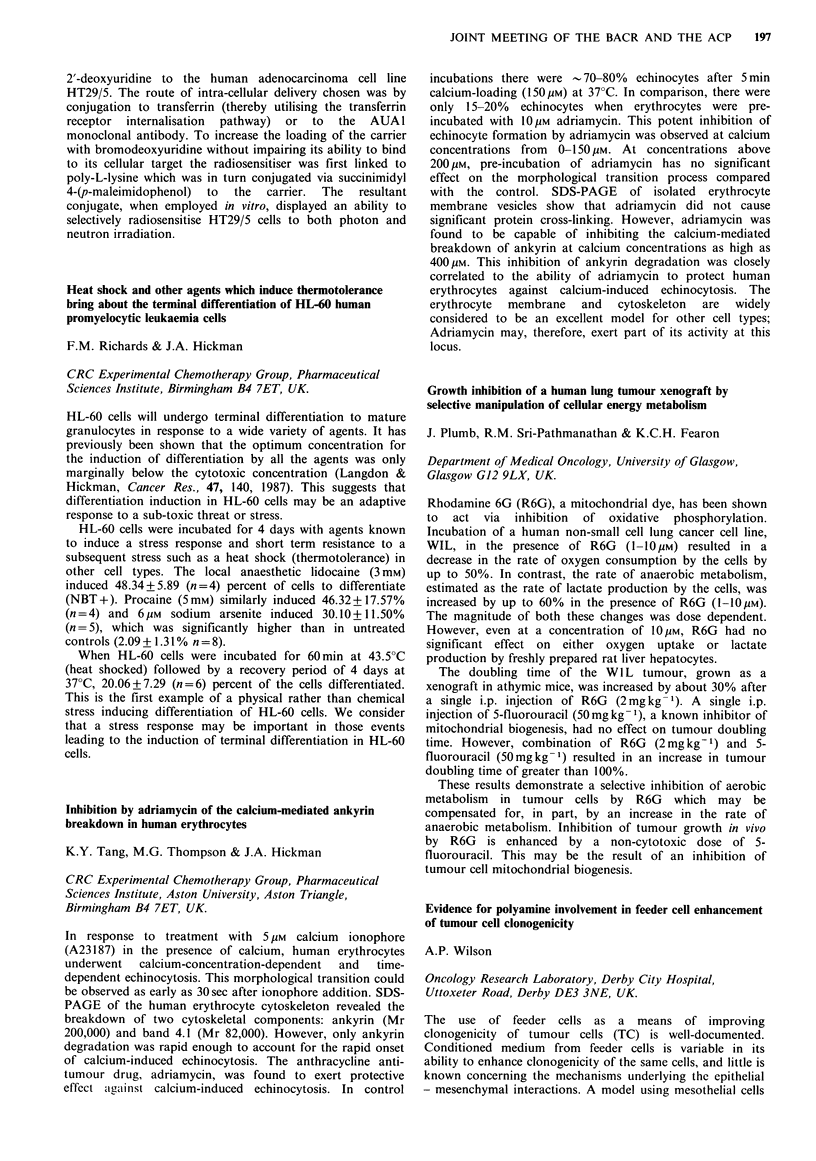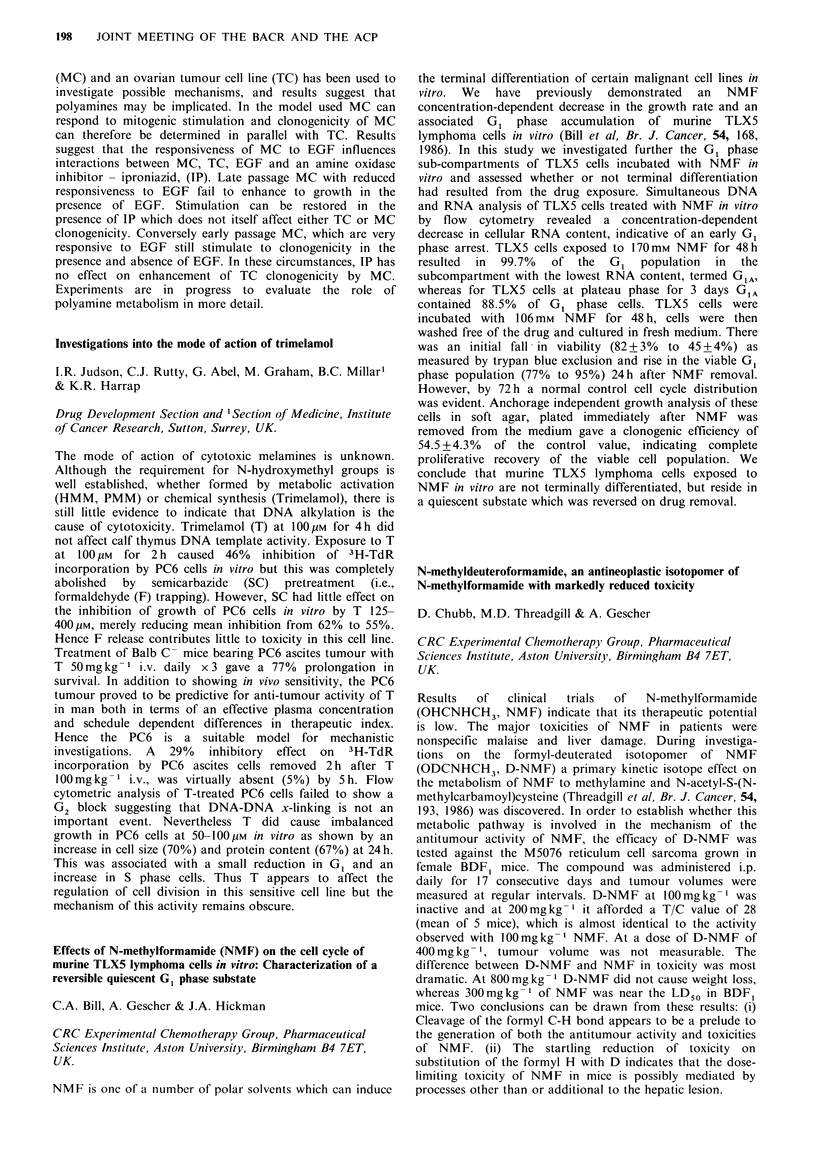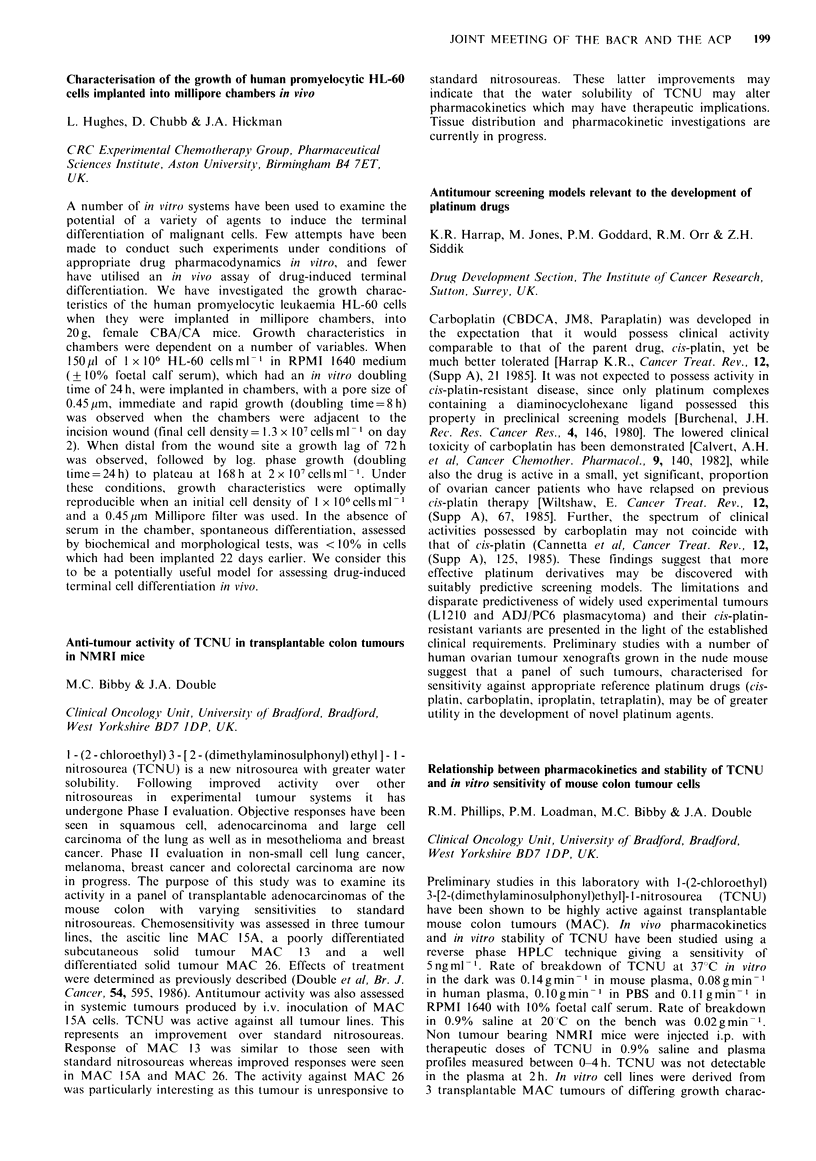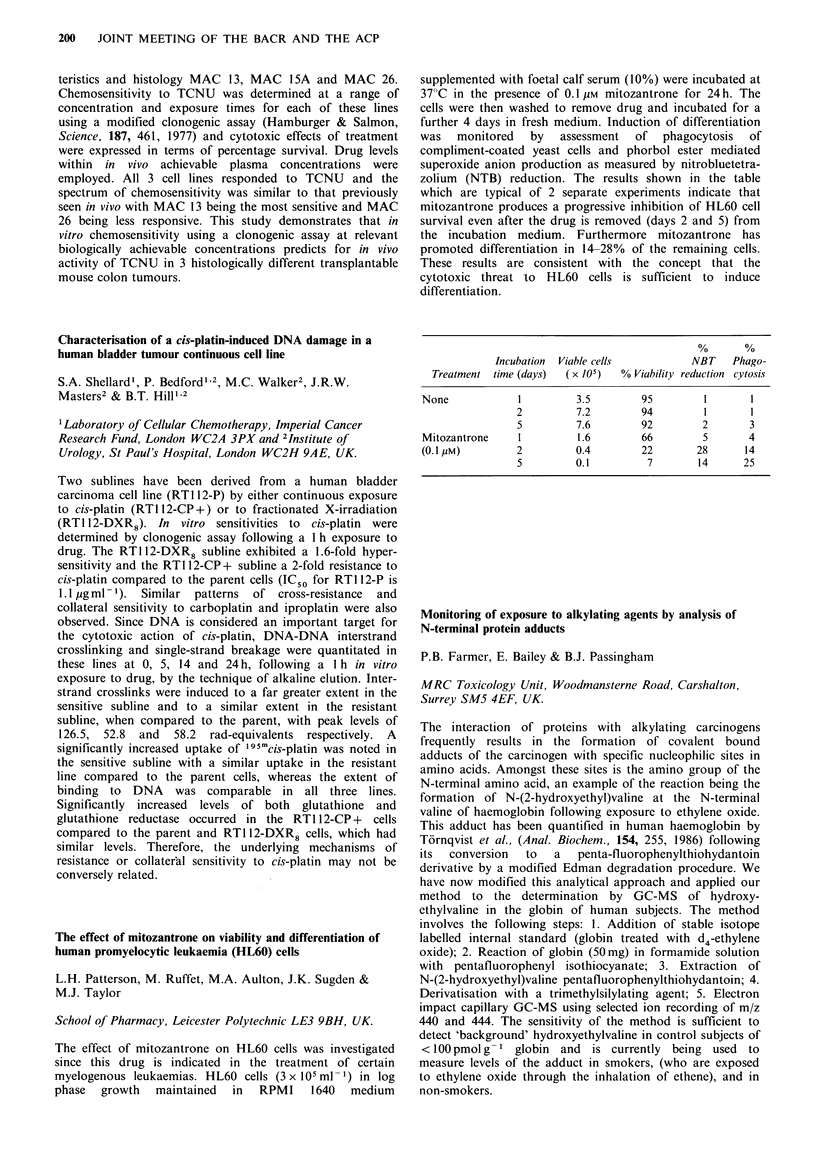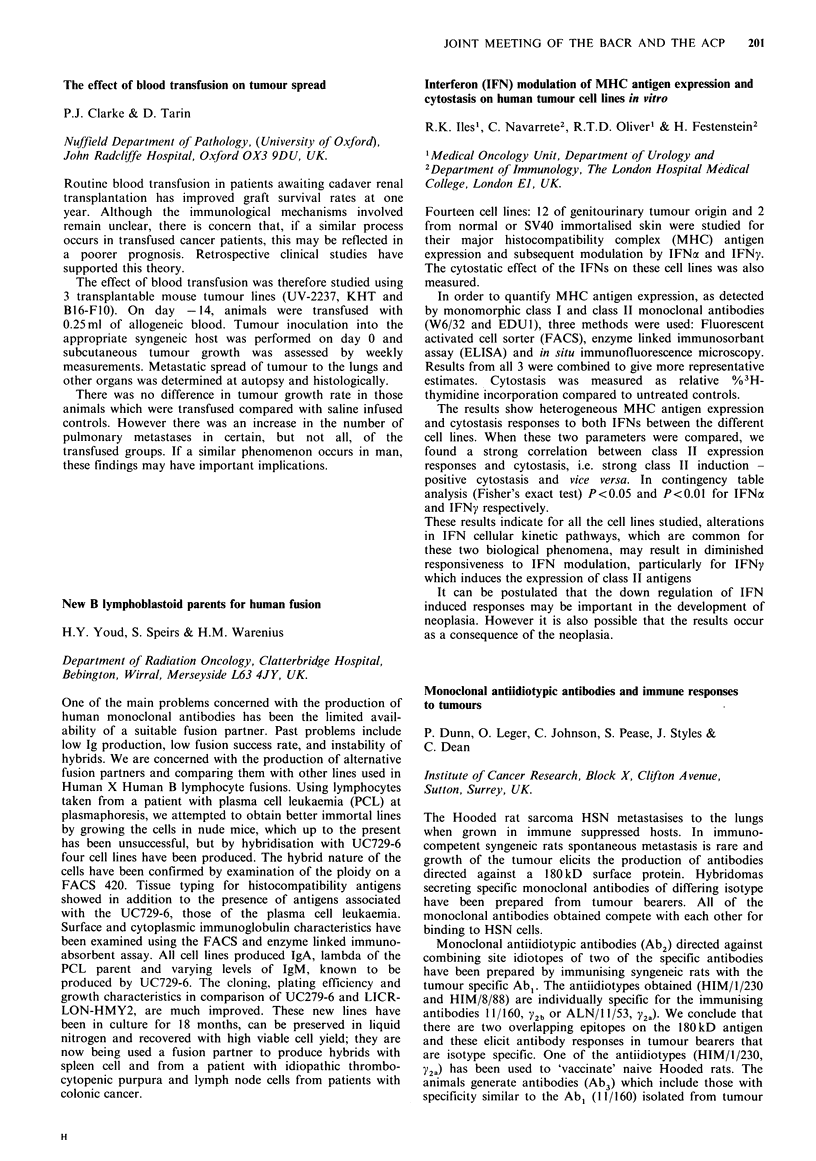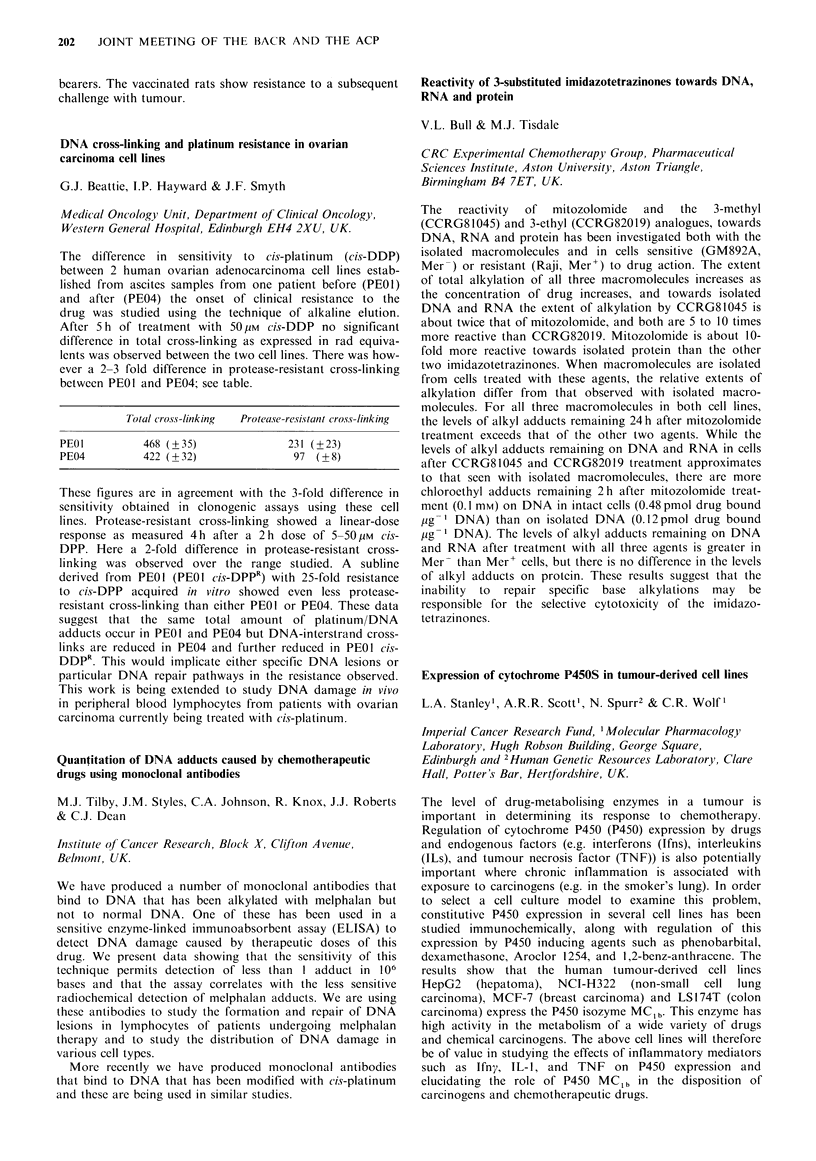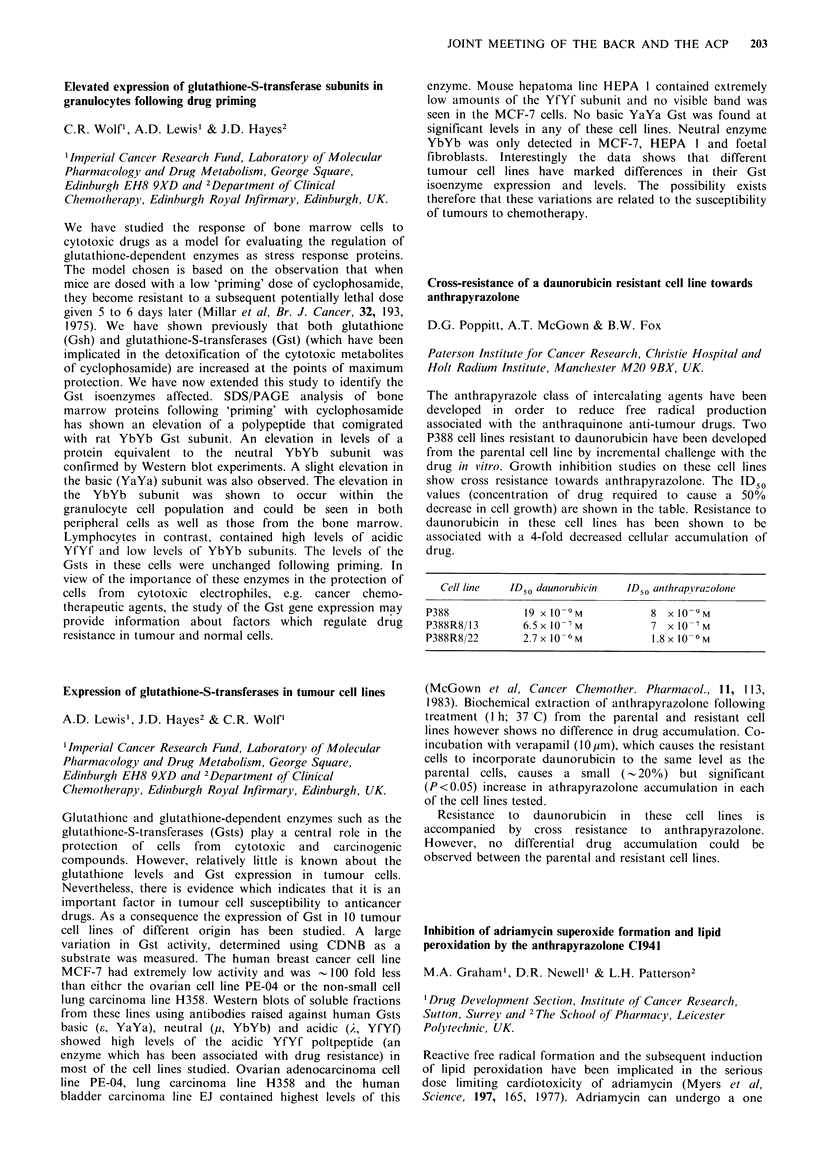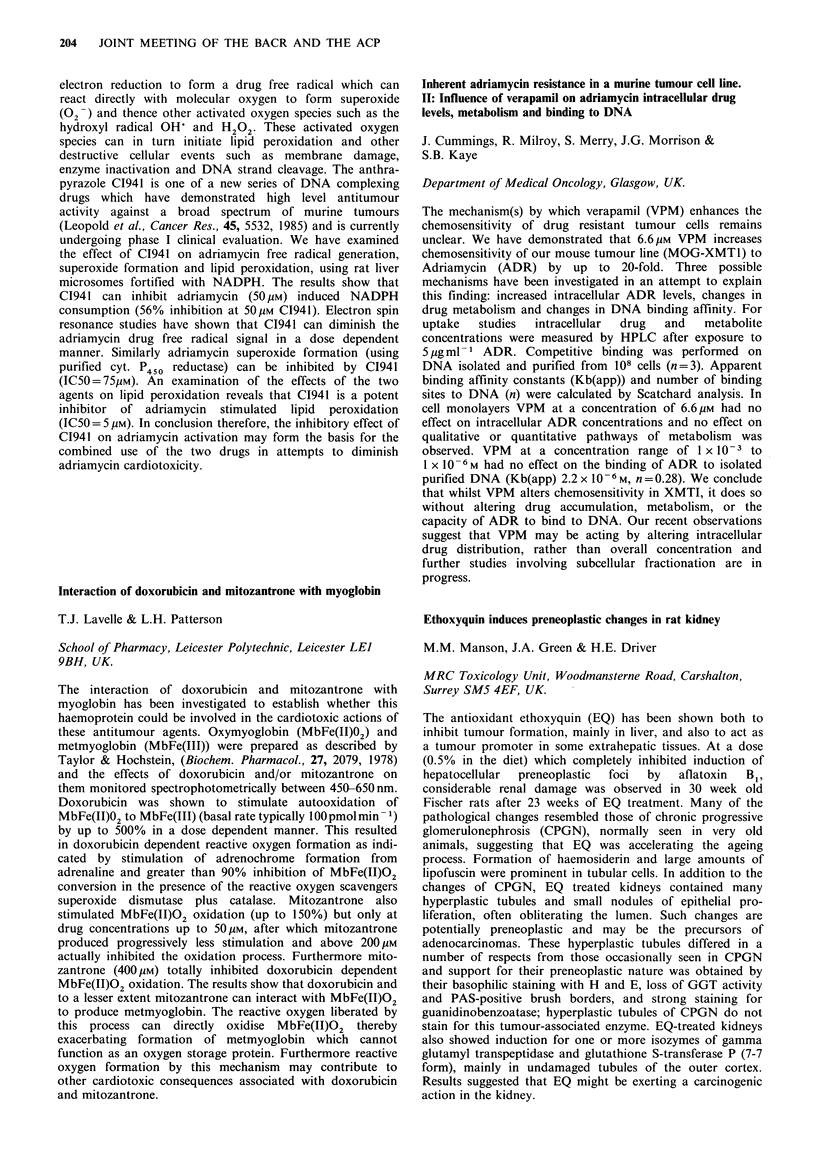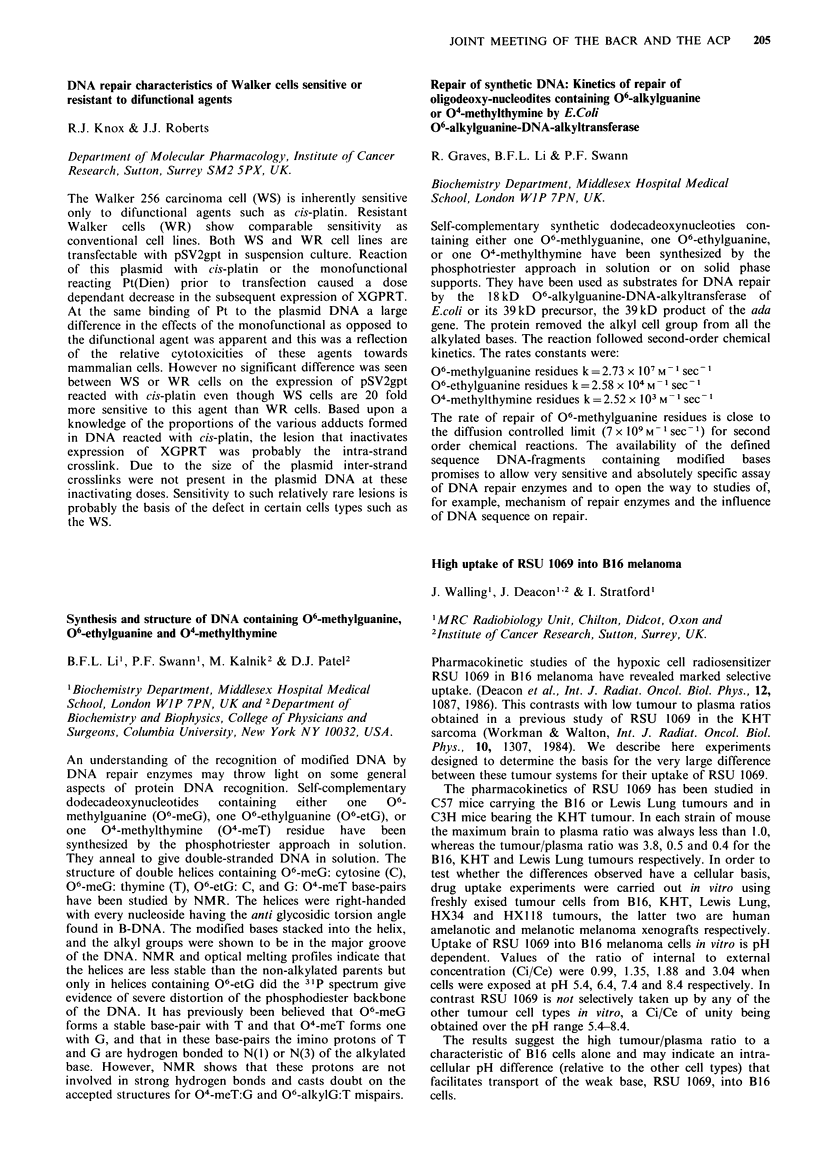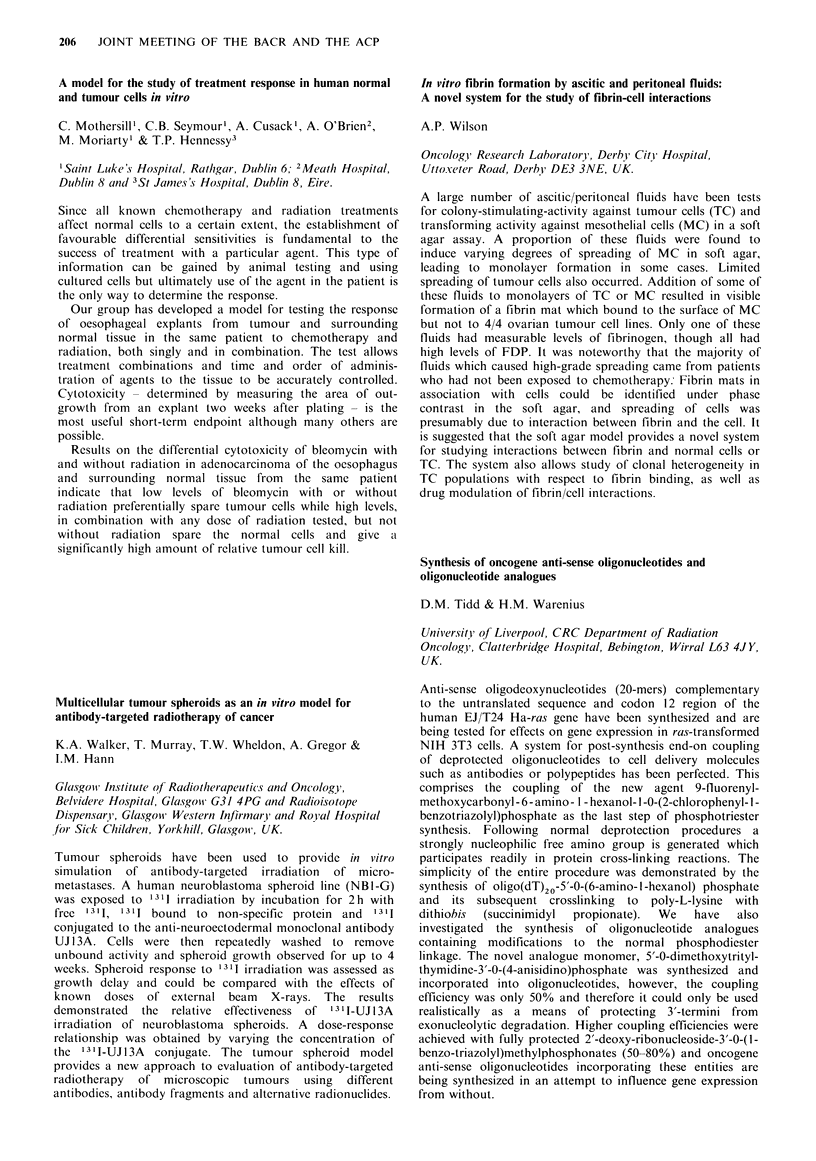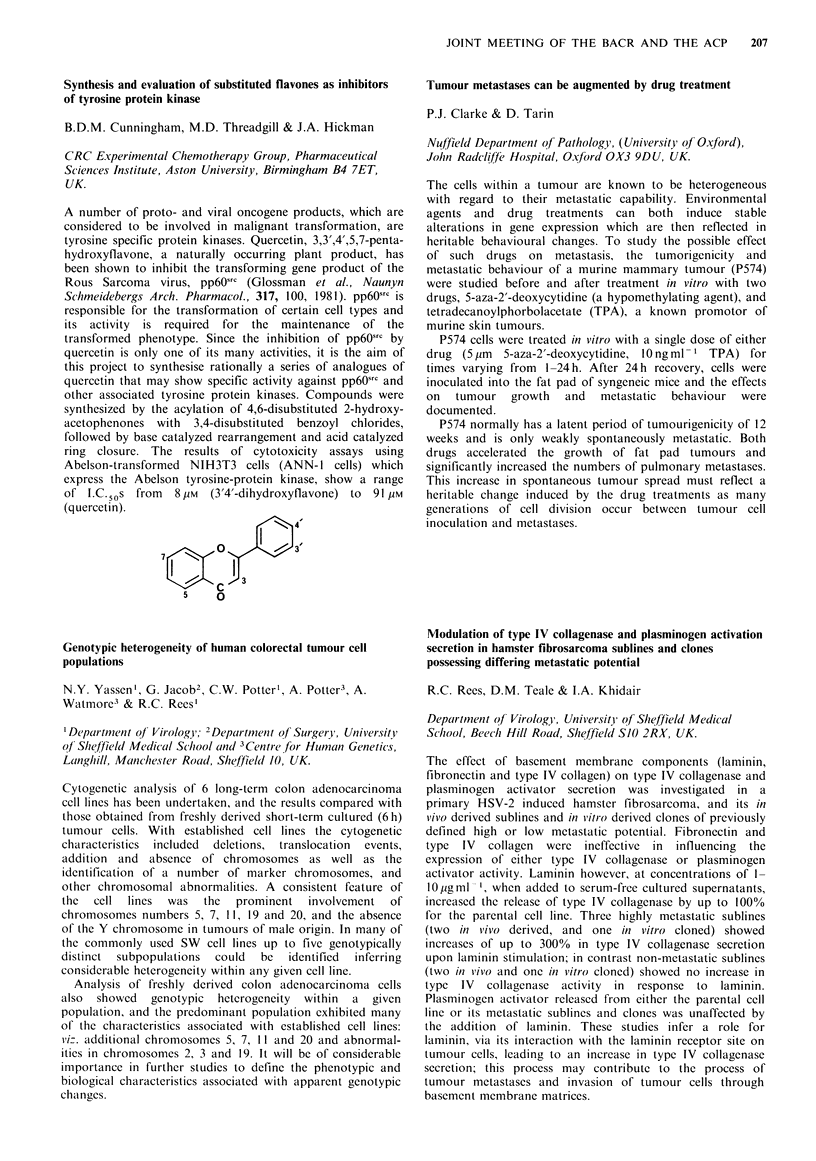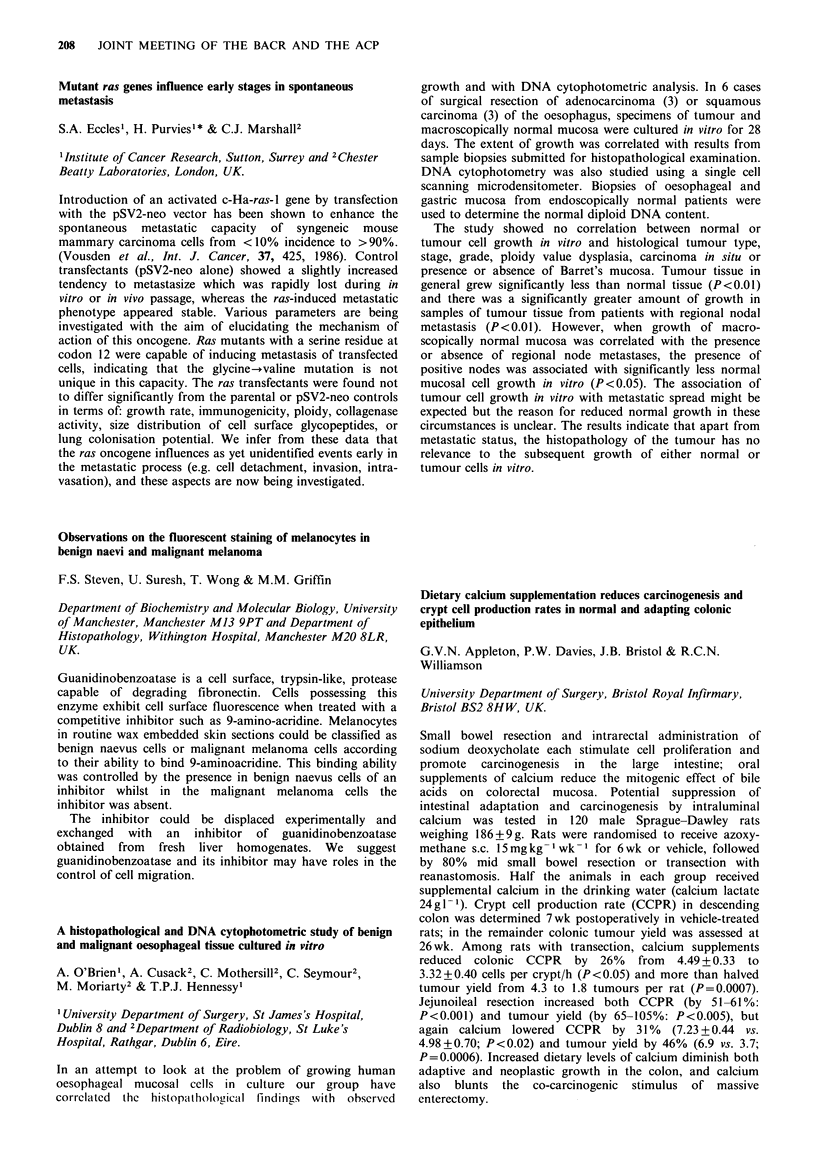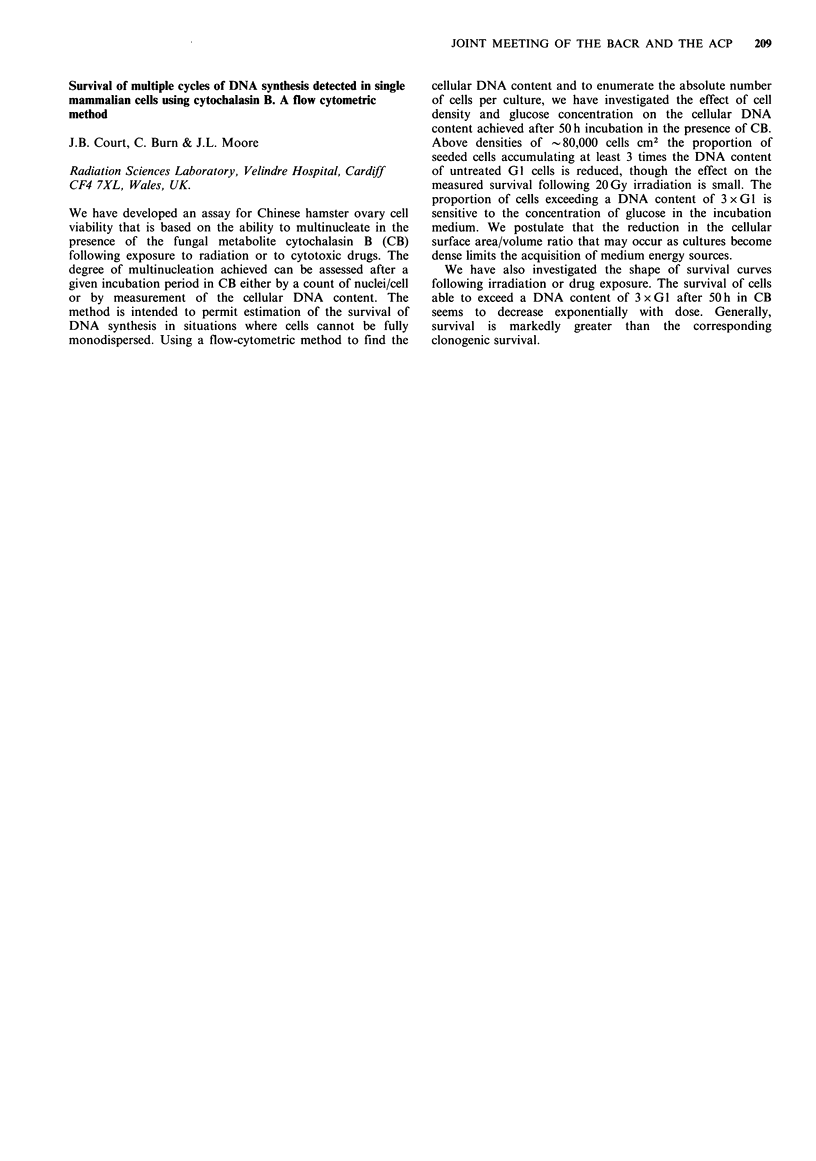# Twenty-eighth annual meeting of the British Association for Cancer Research (in conjunction with the second annual meeting of the Association of Cancer Physicians). April 6-8, 1987, Newcastle-upon-Tyne, UK. Abstracts.

**DOI:** 10.1038/bjc.1987.180

**Published:** 1987-08

**Authors:** 


*Br. J. Cancer* (1987), **56**, 173–209 © The Macmillan Press Ltd., 1987

### Twenty-eighth Annual Meeting of the British Association for Cancer Research* (in conjunction with the Second Annual Meeting of the Association of Cancer Physicians)

(Incorporating Symposia on 'Cancer Metastasis' and 'DNA repair' and the 1987 Walter Hubert Lecture†) April 6–8, 1987.

*Held at the University of Newcastle-upon-Tyne, UK.*

### Abstracts of Invited papers‡

### Symposium on 'Cancer Metastasis and the Generation of the Metastatic Phenotype'

### Growth factors and blood-borne metastasis

P. Alexander

*CRC Medical Oncology Unit, Southampton General Hospital, Southampton S09 4XY, UK.*

Unlike lymphoma and leukaemia cells, carcinoma and sarcoma cells which have gained access to the blood do not circulate but are generally arrested in the first capillary bed encountered. The major sites for metastasis are therefore determined by the venous drainage of the primary tumour. If this is vena caval lung and bone metastases (via vertebral venous shunts) predominate, while the liver is the primary site for metastasis of portal draining tumours. Cells released from primary or secondary lung tumours gain direct access to the arterial circulation and are trapped in all of the different organs of the body in proportion to the blood flow to the individual organs. However, the probability that such a trapped cancer cell develops into a metastasis varies by a factor of $10^4$ between adrenal (highest) and skeletal muscle and gut (lowest) for the rat sarcomas and carcinomas studied. That tissue specific host factors are responsible for this preference is suggested by the finding that tissue injury, such as an incision in skin, an anastamosis of the intestine or mechanical trauma to kidney or liver markedly facilitates metastasis. Growth factors released at sites of injury, particularly coming from infiltrating macrophages allow isolated cancer cells delivered via the blood to grow into macroscopic lesions. In the absence of exogenous stimulating factors, single cells remain dormant and eventually die.

### Genetic aspects of the metastatic phenotype and their interaction with the host micro environment

M. McMenamin & D Tarin

*Nuffield Department of Pathology, John Radcliffe Hospital, Headington, Oxford, UK.*

The work of this laboratory is based on the concept that the driving force of the metastatic process is created by regulatory genomic disturbances in a small population of cells within the primary tumour and that the success or failure of such cells to form a deposit in distant organs is not random but dictated by interactions between the tumour cells showering out from the primary and metabolic conditions encountered in the microenvironment or other organs in the body where they lodge. Superimposed on such local interactions are systemic effects exerted by the immune and endocrine systems, the magnitude of which depends on the type and quantity of exposed antigens and receptors on the disseminating tumour cells as well as on the constitutional vigour of the host. We have adopted a number of different approaches in an attempt to identify the gene or genes involved in metastases. The first approach has been to introduce a defined gene with known oncogenic potential into a previously non-metastatic cell and to observe for phenotypic change. This involved transfections of the c-Harvey *ras* oncogene into a non-tumorigenic non-metastatic cell. A second approach involves transfection of total genomic DNA from metastatic to non-metastatic cells. Finally we have examined whether treatment of weakly metastatic cells with agents known to influence tumour progression and gene expression (e.g. tetra-phorbol acetate or 5-azacytidine) can affect metastatic capability. We shall present results which indicate that, while successful incorporation and expression of the activated c-Ha-*ras* oncogene did not induce non-metastatic 3T3 derived fibrosarcoma cells to become spontaneously metastatic, transfection of the same cell type with DNA from highly metastatic human and animal cell lines did markedly augment their spontaneous metastatic capability and their lung colony forming potential and induce them to form deposits in many extra pulmonary sites. We have also found that treatment of some tumour cell lines with azacytidine and TPA markedly increases their metastatic behaviour after subcutaneous inoculation and, as several cell divisions must have occurred in producing the subcutaneous tumour before the cells disseminated, we consider the change of phenotype to be heritable and probably caused by alterations in gene expression.

These results suggest that components of the metastatic phenotype are heritable, highly conserved in evolution and can be conferred on previously non-metastatic tumour cells by transfer of genomic DNA. However the process is so complex that there is need for caution in interpretation and points requiring further critical evaluation will be discussed.

### Messenger RNAs putatively associated with progression and metastasis of colorectal cancers

G.D. Birnie*[1], P. Elvin[1], I.B. Kerr[2] & C.S. McArdle[3]

*Beatson Institute for Cancer Research[1], Bearsden, Glasgow G61 1BD and University Departments of Pathology[2] and Surgery[3], Royal Infirmary, Glasgow G4 0SF, UK.*

Phenotypic differences between cells result from quantitative

*Enquiries to the BACR Secretariat, c/o Institute of Biology, 20 Queensberry Place, London SW7 2DZ, UK.
†This issue pp. 91–95.
‡Reprints of these abstracts are not available – Ed.

and qualitative differences in the cells' proteins, and these in turn reflect differences in the populations of the mRNAs in the cells. Thus, phenotypic changes associated with the emergence of cells with metastatic potential should be accompanied by changes in the relative abundances of specific mRNAs. Molecular cloning techniques allow the isolation of cDNA probes homologous to previously uncharacterized mRNAs that are associated with a particular cellular phenotype. We therefore constructed recombinant plasmid cDNA libraries representing the poly$(A)^+$ RNAs from normal colonic mucosa and from a liver metastasis from a colonic adenocarcinoma. Screening of these libraries with $^{32}$P-cDNAs transcribed from poly$(A)^+$ RNAs from specimens of 3 normal colonic mucosae, 3 adenocarcinomas and 3 liver metastases identified 34 recombinants that were homologous to RNAs that differed significantly in abundance between normal and neoplastic colon or between primary tumours and metastases.

Five of these recombinants and their homologous RNAs have been characterized further by Southern and Northern blot and RNA dot-blot analyses. These cDNAs, and other isolated from the libraries, may prove to be of use both as diagnostic tools and for defining phenotypic changes associated with tumour progression and metastasis.

### Gene regulation and the control of metastatic behaviour

I.R. Hart, N. Goode & E.J. Ormerod

*ICRF Laboratories, Lincoln's Inn Fields, London, WC2A 3PX, UK.*

Cellular proliferation constitutes a vital part of the metastatic process such that understanding mechanisms involved in the control of mitogenesis may provide insight into possible approaches to the control of secondary tumour growth. Binding of extracellular mitogenic ligands to specific receptors leads to the initiation of active messenger molecules which mediate transduction of the proliferation signal from the cell surface to the nucleus. Recent interest has focused on the possibility that the rapid enhancement of expression of c-*fos* and c-*myc* mRNA plays a role in transduction of the mitogenic signal in the nucleus.

Using two tumour models, a murine reticulum cell sarcoma and a series of melanoma variants (human and murine) of defined metastatic activity, we have begun to examine the response of malignant tumours to a series of mitogenic factors in terms of cellular proliferation, generation of second messenger molecules and expression of the cellular oncogenes c-*fos* and c-*myc*. Metastatic behaviour of neoplastic cells treated in this fashion has been assessed by subsequent injection into immunocompetent, syngeneic mice or immunoincompetent, athymic mice. Early results show that some of the agents which elicit mitogenic responses in normal cells inhibit proliferation, and decrease the metastatic capacity, of their malignant counterparts. Differences also may exist in the response of high and low metastatic lines, derived from a common parent, to similar treatments. The possibility that these variations in growth regulation may provide an approach to therapy currently is under investigation.

### Selection and characterization of metastatic variants in human melanoma xenografts

J.F. Doré, S. Bertrand & M. Bailly

*INSERM U218, Centre Léon Bérard, 28 rue Laënnec, 69373 Lyon Cedex 08, France.*

The selection of human melanoma variants and clones with increased metastatic abilities was attempted by xenografting human melanoma cell lines in athymic nude mice and in immunosuppressed new-born rats.

Subcutaneous transplantation in a nude mouse of a human melanoma metastatic nodule resulted in a subcutaneously growing tumour (NTT) and in spontaneous lung (NTP) and lymph node (NTG) metastases (Neulat-Duga *et al., Invasion Metast.*, **4**, 209, 1984). NTT, NTP and NTG cells were first maintained *in vivo* by subcutaneous passages in nude mice and then cultured *in vitro* as cell lines. Cytogenetic studies of the *in vivo* passaged cells showed that the 3 tumour lines differed in their modal chromosome number. 15 markers were identified, including several common to all 3 lines; one of these, derived from chromosome 7 and containing an HSR, was found with a higher frequency in metastatic tumour lines. In addition, 2 markers derived from chromosome 1 were both present in NTT cells but mutually exclusive in NTP and NTG cells. Thus, all 3 tumour lines have a common origin and metastases in the nude mouse resulted from a selection among cell populations. Following 15 *in vitro* passages, NTP cells were injected s.c. in nude mice: serial transplantation was accompanied by an increase in metastatic ability of tumour cells.

Human melanoma cell lines, tumorigenic but not metastatic in nude mice, were xenografted to ATS immunosuppressed new-born rats. 3 weeks after s.c. injection of $10^6$ cells, nearly all rats developed tumours and a proportion of them lung and lymph node metastases. Agar cloning of M4Beu line showed that it is heterogeneous and contains poorly tumorigenic but highly metastatic cells.

### The generation of metastatic mosaicism in B16 murine melanoma

G.V. Sherbet[1], M.S. Lakshmi[1], J. Lunec[1], S.S. Bhattacharya[2] & C. Parker[1]

[1]*Cancer Research Unit, University of Newcastle upon Tyne, Newcastle upon Tyne NE1 4LP and* [2]*MRC Clinical and Population Cytogenetics Unit, Edinburgh, UK.*

The generation of metastatic heterogeneity is currently receiving considerable attention. The genome of tumours with high metastatic potential has been described as being more labile than that of tumours with low metastatic potential. The rate of generation of drug resistant variants is greater in tumours with high metastatic potential as compared with those with low metastatic potential (Cifone & Fidler, *Proc. Natl Acad. Sci. USA*, **78**, 6949, 1981). We investigated the low metastasis F1 and high metastasis variant BL6 of the B16 murine melanoma for differences in genetic recombination and differential expression of genetic messages.

Genetic recombination, seen as sister chromatid exchange (SCE), increased with increase in metastatic potential. Metastatic tumour cells from lung showed greater SCE than the corresponding primary tumour. Metastatic tumour rejoined bleomycin-induced DNA strand breaks considerably more slowly than the primary tumour (Sherbet *et al., Br. J. Cancer*, **54**, 164, 1986). This reduced repair may be conducive to the exchange of chromatid segments. The SCEs occurred predominantly in a hypertriploid subpopulation. Primary tumours also showed a transition to a predominantly hypertriploid state in the progression to the metastatic state. Alkylating agents such as mitomycin C and ethylmethanesulphonate considerably enhanced the SCE incidence in this hypertriploid subpopulation. It is suggested that metastatic variants may be generated in this genetically unstable subpopulation by a process of genetic recombination.

A cDNA library of the BL6 variant was constructed in λgt 10. A differential screening of this library with cDNA probes prepared from F1 and BL6 cells has shown that some messages are differentially expressed in these variants. The cDNA clones corresponding to these messages have been isolated and are being characterised.

### Joint Symposium (with ACP) on 'DNA repair'

### Molecular cloning of genes involved in the excision repair system of mammalian cells

J.H.J. Hoeijmakers[1], M. van Duin[1], M. Koken[1], A. Westerveld[1], G. Weeda[2], A.J. v.d. Eb[2] & D. Bootsma[1]

[1]*Department of Cell Biology & Genetics, Erasmus University, P.O. Box 1738, 3000 DR Rotterdam and* [2]*Department of Medical Biochemistry, State University Leiden, 2333 AL Leiden, The Netherlands.*

To deepen our understanding of the mechanism and genetic control of mammalian excision repair it is essential to identify the genes and proteins involved. To this aim we have adopted several strategies. One of the most straightforward approaches relies on the 'correction' of repair defective mutant cells by DNA mediated transfer of the normal gene, recruited from genomic DNA of a repair competent cell line. This strategy has been successful using several CHO-mutants, but has failed thus far for xeroderma pigmentosum (XP) cells. We have found that in contrast to (CHO) Chinese hamster ovary DNA repair mutants- SV40 transformed XP-fibroblasts are practically unsuited for genomic DNA transfections because of the limited amounts of exogenous DNA incorporated by these (and other human) cells.

Using a representative of CHO complementation group 2 we have cloned the human excision repair gene *ERCC-1*. The status of the characterization of this gene and its product will be reported. As steps towards the isolation of *ERCC-3* (complementation group 3) and *ERCC-6* (group 6) genes we have generated primary and secondary transformants in transfection experiments of human DNA to excision deficient CHO mutants 27-1 (Dr R. Wood) and UV-61 (Dr L. Thompson). The isolation of the *ERCC-3* gene from cosmid libraries is in progress.

As reported (van Duin *et al.*, *Cell*, **44**, 913, 1986) a significant aminoacid sequence conservation was discovered between the excision repair proteins *RAD10* from yeast and *ERCC-1* from man. This prompted us to examine whether other human genes are conserved as well and whether they can be isolated on the basis of nucleotide homology with cloned yeast repair genes.

### Use of repair defective CHO cells to clone human repair genes

C.A. Weber, K.W. Brookman, J.L. Minkler, E.P. Salazar, S.A. Stewart & L.H. Thompson

*Biomedical Sciences Division, Lawrence Livermore National Laboratory, Livermore, CA 94550, USA.*

The Chinese hamster ovary (CHO) cell line UV5 (which is defective in the incision step of nucleotide excision repair) and EM9 (which has high sister chromatid exchange (SCE) levels and is defective in DNA single and double strand break repair) were used to identify human genes that correct these repair deficiencies and to study repair processes. Repair proficient, *gpt*-expressing primary transformants were obtained by cotransforming mutant cells with the plasmid pSV2*gpt* and DNA from a human/hamster hybrid line. The secondary transformant 5T4-1 (UV5) and the tertiary transformant 9TTT3 (EM9) which are relatively free of human sequences other than a repair gene were obtained by using DNA sheared to <50 kb from primary or secondary transformants, respectively. Cosmid clones containing the correcting human genes (*ERCC2* for UV5 and *XRCC1* for EM9) were identified and purified by screening cosmid libraries made from 5T4-1 and 9TTT3 DNAs using HeLa DNA as the probe. Transformation of mutant cells with these cosmid DNAs demonstrated that 8/21 (UV5) and 2/9 (EM9) have a functional repair gene. In the UV5 study, UV survival curves demonstrate that primary, secondary, and cosmid transformants have, in all but one case, similar or slightly higher levels of UV resistance compared to normal cells (AA8). The levels of UV induced mutation at the *aprt* locus for 5T4-1 and cosmid transformants vary from 50–150% of normal. Measurements of the initial rate of incision using alkaline elution indicate that, while the UV5 rate is 3% of AA8, rates of cosmid transformed lines are similar to AA8 and the 5T4-1 rate is 170% of AA8. In the EM9 study, γ-ray survival curves of 9TTT3 and cosmid transformants show, in most cases, levels of resistance similar to AA8. Primary and cosmid transformants had normal levels of SCE and chromosomal aberrations induced by BrdUrd. Alkaline elution studies measuring single strand break repair after γ-ray exposure in 9TTT3 and cosmid transformants indicate normal levels of repair. Restriction enzyme site maps of the 5T4-1 derived cosmids have been determined and show *ERCC2* is between 15 and 25 kb. The cosmids will be used to obtain the corresponding cDNA for sequencing, analyzing gene structure, and producing the encoded protein.

### DNA damage inducible responses in mammalian cells

P. Herrlich[1], M. Imagawa[2], P. Angel[1], M. Buescher[1], M. Karin[2], H.J. Rahmsdorf[1] & B. Stein[1]

[1]*Kernforschungszentrum Karlsruhe, Institute for Genetics, Postfach 3640, D-7500 Karlsruhe 1, FGR and* [2]*Department of Pharmacology, M036, School of Medicine, University of California San Diego, La Jolla, CA 92093, USA.*

Genotoxic agents as well as mediators of inflammation or substances imitating these mediators such as the phorbol esters cause an active genetic response. This response is largely transient but may have significant long lasting effects on cells. The immediate reaction (minutes after a UV or phorbol ester treatment) includes the induced expression of c-*fos*, proteases, metallothioneins, DNA and RNA viruses. Interesting late endpoints (hours) are the overreplication of genes and the secretion into the extracellular space of a number of factors one of which is mutagenic. Using deletion mutants of the human c-*fos* and collagenase genes we have defined the *cis* acting elements which mediate the induction by UV and by phorbol esters. These elements bind specific regulatory proteins. As an example, the TPA responsive element (TRE) of the collagenase gene ranges from positions −73 to −65. A synthetic oligonucleotide of this sequence (5′-ATGAGTCAG-3′) suffices to confer TPA dependent regulation to the thymidine kinase promoter. By footprinting and competition experiments a specific DNA binding protein has been defined and purified using affinity chromatography. The activity of this trans-acting factor is increased by TPA treatment of cells. TRE sequences occur in several TPA inducible genes. These bind the same factor suggesting that the factor forms the major signal receiving structure for the genetic actions of phorbol esters (Angel *et al.*, *Mol. Cell Biol.*, **6**, 1760, 1986).

### Poly (ADP-Ribose) and chromatin organization in DNA excision repair

F.R. Althaus

*Department of Pharmacology & Biochemistry, University of Zurich-Tierspital, Winterthurerstrasse 260, CH-8057 Zurich, Switzerland*

*De novo* poly ADP-ribosylation of chromatin proteins is a stereotype response of higher eukaryotes to DNA damage. Numerous lines of evidence suggest that this post-translational protein modification modulates chromatin functions. The role of poly ADP-ribosylation in DNA excision repair may be conceptualized as follows. All target proteins of poly ADP-ribosylation hitherto identified share the capacity to bind to DNA. Recent results suggest that reversible poly(ADP-ribose)-modification of these proteins may serve as a general shuttle mechanism for DNA-protein interactions. *In vitro* poly ADP-ribosylation of nucleosomal core particles reduces DNA-protein interactions. *In vivo*, the rapid shuttling of DNA binding proteins on damaged templates may facilitate local changes of chromatin structure in DNA excision repair, such that newly synthesized repair patches appear in 'free' DNA domains with increased accessibility to chemical and enzymatic probes (*J. Biol. Chem.*, **261**, 5758, 1986). This step is completely blocked in poly(ADP-ribose)-depleted mammalian cells treated to repair C-8 substituted deoxyguanosine adducts. As a consequence, dG-8 adducts remain unexcised and accumulate in free DNA domains. By this mechanism, poly ADP-ribosylation of chromatin proteins may modulate the biological expression of DNA damage.

### Characterisation of cytotoxic drug hypersensitive Chinese hamster cells

I.D. Hickson

*Department of Clinical Oncology, University of Newcastle upon Tyne, Royal Victoria Infirmary, Newcastle upon Tyne, NE1 4LP, UK.*

As part of a study of the molecular mechanisms of DNA repair in mammalian cells, we have isolated mutants of a Chinese hamster ovary cell line which exhibit hypersensitivity to DNA damaging agents. We currently have over 20 such mutants, of which 14 are under detailed investigation. Of these, 5 (designated MMC-1 to -5) were isolated on the basis of sensitivity to mitomycin C (MMC), 2 as sensitive to bleomycin (designated BLM-1 and -2), 6 sensitive to MMS (MMS-1 to -6) and 1 adriamycin-sensitive (ADR-1).

Mutants MMC-1 to -5 are 4- to 7-fold hypersensitive to MMC, as judged by $D_{37}$ values, and vary in their cross-sensitivities to other DNA damaging agents. Using alkaline elution to study the induction and repair of DNA interstrand cross-links following exposure to MMC, we have shown that MMC-4 and, more particularly, MMC-5 cells accumulate higher levels of DNA cross-links than do CHO-K1 cells, but repair this damage normally. In contrast, MMC-2 cells (which are 10-fold cross-sensitive to UV light) are defective in the repair of cross-links. Mutants BLM-1 and -2, which are 7- and 14-fold sensitive to bleomycin respectively, differ markedly in their response to bleomycin treatment. BLM-1 cells receive equivalent levels of DNA strand breaks to wild-type cells and are proficient in DNA repair. In contrast, BLM-2 cells (which are 2-fold cross-sensitive to X-rays) not only accumulate higher levels of both single- and double-strand DNA breaks than parental cells, but also repair both forms of lesion with reduced efficiency.

ADR-1 cells accumulate more DNA strand breaks following adriamycin treatment but repair this damage with normal efficiency. In this case breaks are protein-concealed, a characteristic of topoisomerase II-dependent scissions.

By an analysis of the drug sensitivity of cell hybrids, we have shown that the MCC, bleomycin and adriamycin-sensitive mutants are all phenotypically recessive, and represent 7 different complementation groups. Only MMC-1 and -5 are genetically identical. Complementation analysis with the MSS sensitive lines is in progress.

Analysis of spontaneous mutation frequencies in the MMC and MMS sensitive lines reveals several alterations from wild-type. Mutants MMC-4 and MMC-5 show respectively a 10-fold elevated and a 2-fold reduced mutation frequency to thioguanine resistance. Of the MMS sensitive lines, MMS-1, MMS-2 and MMS-5 have a mutator phenotype, while MSS-4 is hypomutable. Differences also exist in the frequency of drug-induced mutations in these lines.

Following DNA transfection with a human gene bank, we have isolated drug-resistant derivatives of MMC-4 and BLM-2 that contain integrated human DNA sequences. Work is in progress to recover this DNA by cosmid rescue.

### DNA repair and drug resistance – Clinical relevance

A.L. Harris

*Cancer Research Unit, Royal Victoria Infirmary, Newcastle upon Tyne NE1 4LP, UK.*

A minority of human tumours are curable with chemotherapy (lymphomas, acute leukaemias, childhood tumours, choriocarcinomas, teratoma; ovarian and oat cell lung cancer, rarely). In these cases, tumours are responsive to doses of cytotoxic drugs that are not excessively toxic to the host. This suggests that there are hypersensitive populations of tumour cells that can be eliminated or greatly reduced. *In vitro* study of cell lines from chemosensitive tumours does show they are more sensitive *in vitro* – nearly as sensitive as ataxia telangiectasia cells. Thus defective DNA repair may be a common basis for the drug sensitive tumours – perhaps relating to certain stages of normal differentiation.

A comparison of the degree of drug sensitivity and resistance with the mechanisms of sensitivity and resistance in a family of CHO mutants and other cell lines suggests that at different degrees of resistance different mechanisms predominate. DNA repair by various mechanisms is a major determinant of sensitivity over the range of 14-fold sensitive to 3-fold resistant. Thus the responsiveness of curable tumours may be particularly determined by repair.

At levels of 10-fold resistance, 2 main mechanisms predominate – P-glycoprotein and glutathione transferases – neither of which may require gene amplification. Gene amplification for particular target enzymes or resistance genes (P glycoprotein) can produce 10–1000-fold resistance.

The degree of resistance or sensitivity that is clinically relevant is probably 3-fold – this would make the difference between resistance or response. Thus DNA repair is probably most important in the chemocurable tumours.

$0^6$ methyltransferase is the most clear case of a repair enzyme being related to resistance. It can be depleted in cells by the base $0^6$ methylguanine. In most human tumours the enzyme level is lower than in normal tissues so it may be possible to lower the level below a critical threshold in tumours compared with normal tissue and sensitise tumours selectively.

Recovery from DNA damage is a more pleiotropic response than repair of specific lesions. The factors relating to poor prognosis of tumours obviously are also related to failure of therapeutic modalities, e.g. poorly differentiated tumours, high stage tumours. Since the factors relating to cell growth may also be related to recovery from DNA damaging drugs and radiation, we investigated EGF receptors in 2 common epithelial carcinomas – breast cancer and bladder cancer. In each case, EGFr were related to poor differentiation of the tumour and also were the most significant prognostic factor in the primary tumour, independently of the stage of the tumour. The role of the EGFr in modulating resistance to cytotoxic drugs is currently being evaluated.

In *E. coli* there are several inducible responses to DNA damage and an increasing number of enzyme activities are being shown to be increased by DNA damage in human cells. Glutathione transferases have a direct DNA repair function, they can convert hydroperoxythymine residues to hydroxyuracil, which is then a substrate for a specific glycosylase. To investigate inducible responses in mammalian cells, we have made CHO cells resistant to adriamycin, mitomycin C or chlorambucil.

The chlorambucil resistant line is cross-resistant to melphalan and nitrogen mustard, but no other alkylating or cytotoxic drugs. A cytoplasmic protein is increased 10-fold in abundance and may be a basic glutathione transferase (GT). At equitoxic doses of nitrogen mustard there are equal crosslinks and isolated nuclei produce equal numbers of crosslinks at equimolar doses of nitrogen mustard. If GTs are inducible by DNA damage, this will be an important protective mechanism in a pleiotropic mammalian response to DNA damage. They may also be relevant to normal tissue distribution of damage by cytotoxic drugs.

### Complementation of alkylation repair deficiencies by gene transfer

G.P. Margison

*Department of Carcinogenesis, Paterson Institute for Cancer Research, Christie Hospital and Holt Radium Institute, Manchester M20 9BX, UK.*

Exposure of mammalian cells to alkylating agents can result in mutation, chromosome damage, transformation or cell death. The contribution of individual DNA lesions to these effects can be investigated using cloned genes that code for specific DNA repair functions to complement repair deficient cells. Results thus obtained with Chinese hamster V79 and murine haemopoietic stem cells indicate that the formation of $0^6$-alkylguanine in DNA can be responsible for the toxic, mutagenic and clastogenic effects of certain alkylating agents, particularly chloroethylating agents.

### Trichothiodystrophy – A UV-sensitive disorder

A.R. Lehmann & C.F. Arlett

*MRC Cell Mutation Unit, Sussex University, Falmer, Brighton BN1 9RR, UK.*

Trichothiodystrophy (TTD) is an autosomal recessive disorder characterised by brittle hair with reduced sulphur content, ichthyosis, peculiar face and mental and physical retardation. Some patients are photosensitive. A previous study by Stefanini *et al.* (*Human Genet.*, **74**, 107, 1986) showed that cells from 4 patients with TTD had a molecular defect in DNA repair, which was in the same complementation group as xeroderma pigmentosum, group D. We have carried out a detailed molecular and cellular study of the effects of UV light on cells cultured from 4 further TTD patients and have found a variety of different responses. Cells from patient 1 were normal in cell survival, excision repair, DNA and RNA synthesis following UV irradiation, whereas in cells from patient 2 all these responses were similar to those of excision-defective XP cells. In cells from patient 3 cell survival was normal following UV-irradiation, even though excision repair was only 50% of normal, and RNA synthesis was severely reduced. In patient 4 excision repair was normal but RNA synthesis was reduced. Our results suggest that the abnormal UV response of most TTD cell strains may be used for confirmation of the clinical diagnosis and for prenatal diagnosis of TTD. They pose a number of questions about the relationship between the molecular defect in DNA repair and the clinical symptoms of XP and TTD.

### Repair of X-ray induced DNA damage in mutant mammalian cells

J. Thacker

*MRC Radiobiology Unit, Chilton, Didcot, Oxon OX11 0RD, UK.*

The nature and complexity of the processes which repair ionising radiation damage in mammalian cells are largely unknown. An important approach to understanding these processes is to identify and characterise mutants with an altered capacity to recover from radiation damage. A human mutation, leading to the syndrome ataxia telangiectasia (A-T), gives cancer proneness and radiosensitivity at both tissue and single cell levels but as yet there is no consensus on the molecular basis of the disorder. Recently mutants have been sought from established mammalian cell lines with some success: we have isolated 3 new mutants and have shown by cell fusion studies with these and other recently-isolated mutants that there are at least 6 genes controlling radiation sensitivity in hamster cells. Several of these mutants are being used as vehicles for the molecular cloning of the normal human genes compensating for their defects.

Our group has made particular use of gene transfer and recombinant DNA methods to characterise radiation-sensitive mutants. Measurements of the rejoining of transferred genes carrying a double-strand break at specific sites have revealed that A-T cells have a significant reduction in rejoin fidelity. Molecular analysis of rejoined molecules characterized this loss of fidelity as deletion of sequence around the break site. One of our X-ray sensitive hamster mutants (*irs1*) shows similar loss of rejoin fidelity with this assay, while other hamster mutants show normal fidelity levels. Radiation damage may give sufficient loss of genetic information to require repair by recombination of DNA helices. Using gene transfer assays we have shown that the xrs series of hamster mutants have a large decrease in ability to integrate DNA into their genomes, presumably by non-homologous recombination, while homologous recombination is little affected.

### Metabolic inhibitors: Tools for dissecting DNA repair

A. Collins

*Department of Biochemistry, University of Aberdeen, Marischal College, Aberdeen AB9 1AS, UK.*

DNA excision repair in mammalian cells is a sequence of reactions about which surprisingly little is known. A biochemical approach that has yielded valuable information makes use of known inhibitors of DNA metabolism. For instance, inhibitors of DNA polymerase cause the accumulation in UV-irradiated cells of incomplete repair sites, seen as breaks in the DNA. (However, paradoxically, repair DNA synthesis seems not to be reduced.) Hydroxyurea, a well known inhibitor of ribonucleotide reductase, causes depletion of the cellular pool of DNA precursors, and consequently inhibits DNA repair.

Can these artificial manipulations of repair be related to the 'real world'? First, many cells in an organism are in a non-dividing state with small DNA precursor pools, resembling hydroxyurea-treated growing cells. Second, cells of certain human diseases show a delay in completion of repair (i.e. breaks accumulate); while in other cases, the response of cells to DNA repair inhibitors can be diagnostic. Third, various therapeutic drugs are found among the known DNA repair inhibitors.

Interrupting DNA repair leads to increased cell killing. It might also be expected to contribute to mutagenesis. Recent work suggests that this is the case.

### The therapeutic use of radiosensitizers and their relation to DNA repair inhibition

I.J. Stratford

*MRC Radiobiology Unit, Chilton, Didcot, Oxon OX11 0RD, UK.*

Radiation causes damage to cells via fast free radical processes. Time resolved techniques, such as pulse radiolysis and rapid mixing, have identified many of the radical species likely to be responsible for damage and, in addition, have shown how the fate of these radicals can be modulated by protectors e.g. thiols, or sensitizers e.g. electron affinic agents. The molecular changes caused by radiation occur as a function of direct energy deposition in the target molecules or as a consequence of the interaction of radiolytic products of water, primarily OH radicals, with DNA.

Hypoxic cells are radiation resistant and can contribute to failure of radiotherapy. Thus, development of methods for increasing the radiation sensitivity of these hypoxic cells would be important. This can be done by enhancing the types of molecular damage alluded to above by using electron affinic nitroimidazoles or by depleting cellular thiol pools.

### Cross-linking and drug resistance

B.W.Fox & P.M. O'Connor

*Paterson Institute for Cancer Research, Christie Hospital and Holt Radium Institute, Manchester M20 9BX, UK.*

The dimethanesulphonate esters present an interesting series of agents which do not require metabolic activation and apart from the basic interaction of the alkylating groups with the target site, do not produce significant toxic or other biologically active metabolic products. The level of unrepaired DNA-DNA interstrand crosslinks is a critically important lesion which would seriously impair mitosis and subsequent integrity of a cell. Based on microbial evidence, the level of such cross-linking necessary to kill a cell is probably beyond the limits of detection by techniques currently available. Thus any meaningful relationship of the sensitivity of the cell to the degree of unresolved crosslinking, at pharmacologically sensible levels of drug treatment, cannot be drawn for many bifunctional antitumour agents. Reverse extrapolation of the levels of DNA interstrand crosslinking from a number of bifunctional antitumour agents, measured at supralethal dose levels suggest that less than fifty unresolved interstrand crosslinks per cell are associated with an LD50 level of the drug. The high level of drug needed to carry out the experiment adversely affects the repair systems themselves. The problems associated with the modelling of such agents for cross-linking activity have been studied in relation to a series of ali-cyclic and aromatic dimethanesulphonates. The basic chemistry of these agents in the environment of the lesion determines the type of DNA-protein cross-linking, which in turn may determine the sensitivity of the cell.

### Abstracts of members' proferred papers

### DNA repair

### Complementation of a DNA repair defect in a mammalian cell by expression of a cloned bacterial gene

J. Hall, H. Kataoka & P. Karran

*Imperial Cancer Research Fund, Clare Hall Laboratories, Blanche Lane, South Mimms, Herts EN6 3LD, UK.*

Mammalian expression vectors derived from pSV2gpt and encoding all or part of the *E. coli ada*$^+$ gene have been constructed. Following transfection and stable integration into Mex$^-$ CHO cells, the whole *ada*$^+$ gene conferred resistance to both cell killing and mutagenesis by MNNG. An N-terminal fragment of the *ada*$^+$ gene, which encodes only the methylphosphotriester DNA repair domain, did not significantly protect CHO cells against MNNG toxicity or mutagenesis. These observations suggest that the increased resistance to cell killing observed in these transfected cells is due to their higher levels of $O^6$-methylguanine-DNA methyltransferase activity. A third plasmid has been constructed which contains only the coding information for the C-terminal $O^6$-methylguanine-DNA methyltransferase repair domain of the Ada protein. Following transfection into CHO cells, 3 cell lines expressing this bacterial polypeptide have been cloned. All 3 clones exhibit enhanced resistance to killing by MNNG. However, the degree of resistance exhibited is different, despite the fact that their overall levels of $O^6$-methylguanine-DNA methyltransferase measured in cell free extracts are the same. This suggests that variations in expression of $O^6$-methylguanine-DNA methyltransferase activity may exist among cells within a cloned population. This variability may provide a model for the Mex$^-$ phenotype in certain transformed human cell lines.

### Forward and reverse mutation at the HPRT locus is decreased in Chinese hamster cells expressing *E. Coli* alkyltransferase

M. Fox[1], J. Brennand[2] & G.P. Margison[1]

[1]*Paterson Institute for Cancer Research, Manchester M20 9BX and* [2]*CBL, ICI, The Heath, Runcorn, Cheshire, UK.*

In order to further assess the importance of $O^6$-alkylguanine ($O^6$-AG) in the mutagenic effects of alkylating agents, we have transfected Chinese hamster cells with the mammalian cell expression vector pZipenoSV(X)1 containing sections of the *E. coli ada* gene that code for $O^6$-AG and alkylphosphotriester (AP) alkyltransferase (ATase) or $O^6$-AG ATase alone. The recipients were wild type and hypoxanthine phosphoribosyl transferase deficient ($HPRT^-$) line in both of which endogenous ATase activity was almost negligible (2–4 fmol mg$^{-1}$ protein). Following transfection a total of ~40 G418 resistant colonies were screened for ATase activity and positive clones expressed between 250 and 1500 fmol ATase mg$^{-1}$ protein. The mutagenic responses of the transfected cells have been analysed at the HPRT locus. At equitoxic doses a 50-fold reduction in MNU induced mutation ($HPRT^+ - HPRT^-$) in cells transfected with the dual function gene and 4-fold reduction in cells transfected only with the $O^6$-AG ATase section was observed. Similar effects were obtained when cell lines were mutagenised with EMS. In the more specific reverse mutation assay $HPRT^- - HPRT^+$ significant protection against the mutagenic effects of a number of monofunctional alkylating agents was also observed. There was a good correlation between the levels of ATase expression in the different cell lines and the reduction in mutagenic effectiveness observed. The data indicate a critical role for alkylation at the $O^6$ position of guanine in both forward and reverse mutation at the HPRT locus.

### Flow cytometric studies on the effects of an ADP-Ribosyl transferase inhibitor in mouse leukaemic cells

P.J. Smith, J.V. Watson & S. Shall

*MRC Clinical Oncology and Radiotherapeutics Unit, Cambridge and School of Biological Sciences, University of Sussex, UK.*

The inhibitor 3-aminobenzamide (3AB) has been used extensively to study the role of poly(ADP-ribosyl)ation in specific cellular functions including DNA repair, pathways for drug cytotoxicity and differentiation. To clarify interpretation of such studies we have used various flow cytofluorimetric techniques to examine the effects of 3AB alone on cell cycle traverse, RNA synthesis and mitochondrial function (by rhodamine uptake). The responses of L1210 cells were compared with those of a resistant variant (L25A). Continuous exposure of L1210 cells to 3AB(5–30 mM) caused a concentration dependent decrease in cell cycle traverse and an eventual (at 24–48 h) G2 block. L25A cells grew more slowly and maintained a greater number of cells in G2. At cytostatic concentrations (25 mM) for L1210 cells G1 emptying was inhibited for a period of 10 h prior to S phase recovery and recruitment into G2 block. Cells blocked in G2 could no longer undergo endo-reduplication and commitment to mitosis appeared to be a lethal event. 3AB (5–30 mM; 2–24 h exposure) reduced cellular RNA content in all phases of the cell cycle and cytostasis was associated with the inability to reach a threshold RNA:DNA ratio. Tests for mitochondrial function showed that cells exposed to 5–25 mM 3AB for up to 24 h were metabolically active, indeed there was a drug related enhancement of rhodamine 123 uptake in S phase cells suggesting an increase in the mitochondrial transmembrane potential. We conclude that 3AB not only modifies the probability of cell cycle transit by altering cellular RNA content but also changes the metabolic status of cycling cells – factors which should be considered in interactive studies with cytotoxic agents.

### The repair of $O^6$-n-butylguanine and $O^2$- and $O^4$-n-butylthymine in rat liver

K. Hellstern, K. Myers & R. Saffhill

*Paterson Institute for Cancer Research, Christie Hospital and Holt Radium Institute, Manchester M20 9BX, UK.*

Butylating agents are potent carcinogens (Druckrey *et al.*, *Z. Krebsforch.* **69,** 103, 1967) but their mechanism of tumour induction has been little studied. However, by analogy with methylating carcinogens promutagenic DNA modifications are most likely to be responsible for the initiation of malignant transformation. Until recently little was known of the reaction of butylating agents with DNA. n-*N*-Butyl-*N*-nitrosourea (BNU) reacts with DNA *in vitro* to form the expected range of products (Saffhill *et al.*, *Biochem, Biophys. Acta.*, **823,** 111, 1985) which include $O^6$-nBug, and $O^4$-nBuT in addition to sec-butyl adducts arising from a rearrangement of the butyl group (Saffhill, *Carcinogenesis*, **5,** 621, 1984). Using radioimmunoassay methods, the repair of $O^6$-nBuG has been observed in CHO cells following treatment with BNU even though the corresponding methyl adduct is not repaired. We now report the formation and repair of $O^6$-nBuG, $O^2$-nBuT and $O^4$-nBuT in the DNA of rat liver following *in vivo* treatment with BNU. Two hours following treatment, the levels of the butyl adducts in the DNA were 14.7, 8.5 and 23.7 umol mol$^{-1}$ of parent base respectively for $O^6$-nBuG, $O^2$-nBuT and $O^4$-nBuT and the adducts were removed with half times of 6, 5 and 4 h respectively.

### ADP-Ribosyl transferase inhibitors potentiate the cytotoxicity of base, but not nucleoside, analogues

K. Moses, B.W. Durkacz & A.L. Harris

*Cancer Research Unit, University of Newcastle upon Tyne, Royal Victoria Infirmary, Newcastle upon Tyne NE1 4LP, UK.*

The base analogues, 6-mercaptopurine (6MP) and 6-thioguanine (6TG), are widely used in the treatment of childhood and adult leukaemias. Since inhibitors of ADP-ribosyl transferase (ADPRT) are known to potentiate the cytotoxicity of monofunctional alkylating agents, we investigated the effects of these inhibitors (the benzamides) on the cytotoxicity of a range of base and nucleoside analogues in CHOK1 cells. 3 mM 3-aminobenzamide potentiated the cytotoxicity of 6TG with a dose enhancement factor (DEF) at 10% survival of 2.0, and 6MP with a DEF of 3.1. Similar results were obtained with a range of ADPRT inhibitors. The cytotoxicity of the nucleoside analogue, 6-thioguanosine, is not potentiated by the benzamides. Likewise, the benzamides potentiated the cytotoxicity of 5-fluoro-1-uracil, but not 5-fluorodeoxyuridine. These observations may reflect a differential effect of the benzamides on the transport of nucleosides and bases. We are investigating the effect of the benzamides on transport. Using the nucleoid technique, there was an increase in DNA strand breaks with increasing 6TG concentrations. No further increase in strand breaks was detected when acetyl-aminobenzamide was present. There is no change in the NAD (which is the substrate for ADPRT) levels of 6TG treated cells. This contrasts with the effect of monofunctional alkylating agents which result in a decrease in NAD levels, and an increase in the number of strand breaks in the presence of the benzamides. Using synchronised cells, we have shown that to obtain potentiation of cytotoxicity of 6TG, the benzamides must be present during the G1 phase of the cell cycle. These results suggest that, for 6TG and 6MP, the potentiation of cytotoxicity is not mediated by an inhibition of DNA repair, which is the widely cited mode of action of the benzamides.

### The role of poly(ADP-Ribose) synthetase in the biological effects of benzamides

M.R. Purnell, J.M. Lunn & A.L. Harris

*Cancer Research Unit, University of Newcastle upon Tyne, Royal Victoria Infirmary, Newcastle upon Tyne NE1 4LP, UK.*

3-Aminobenzamide has been used extensively as a probe for the function of poly (ADP-ribose) synthetase. However, doubts have been cast as to its specificity at the concentrations required to elicit a biological response. To investigate the role of poly(ADPR) in proliferation and MNU potentiation, we have used 4 different benzamides with differing $k_i$ values in human lung adenocarcinoma A549 cells. 3-Acetamidobenzamide (AAB), 3-methoxybenzamide, benzamide and 3-aminobenzamide inhibited cell proliferation in order of potency of their $k_i$ values; more rigorous analysis revealed that a plot of $IC_{50}$ *vs.* $k_i$ (poly(ADPR)) was convex, suggesting that another process may be involved at very high (>10 mM) concentrations. This was confirmed by the observation that potentiation of MNU, which required lower inhibitor concentrations, correlated better with $k_i$ than did inhibition of proliferation. Nicotinamide starvation lowered the $IC_{50}$ for AAB, suggesting that inhibition of proliferation was mediated via a NAD dependent process. Benzamides have been reported to affect glucose transport and metabolism. To assess the contribution of this on proliferation, we studied the influence of glucose concentration on inhibition of proliferation and potentiation of MNU cytotoxicity. The $IC_{50}$ for AAB was not significantly altered by changing the glucose concentration from $0.3\,g\,l^{-1}$ to $3\,g\,l^{-1}$. Potentiation of MNU toxicity was only slightly increased (~20%) when the glucose concentration was decreased from $2\,g\,l^{-1}$ to $0.2\,g\,l^{-1}$, suggesting perturbation of glucose metabolism plays a very minor role in the enhancement of MNU cytotoxicity. We therefore conclude that poly(ADP-ribose) synthetase plays the major role in the mechanism of action of benzamide with regard to inhibition of proliferation and potentiation of MNU cytotoxicity in these cells.

### Characterization of the corrected UV excision repair in a human XPD-like hybrid cell transfected with the phage T4 *DENV* gene

J. Arrand, S. Squires, N. Bone & R.T. Johnson

*CRC Mammalian Cell DNA Repair Group, Department of Zoology, Cambridge, UK.*

A plasmid carrying the T4 *denV* gene, encoding the pyrimidine dimer-specific endonuclease V, and the $neo^R$ gene (Valerie, *et al., Proc. Natl Acad. Sci. USA*, **82,** 7656, 1985) has been introduced by DNA transfection into a UV excision repair defective human cell. High transfection frequencies were achieved ($2–5 \times 10^{-3}$) when both markers, $neo^R$ and $UV^R$, were selected. 15 clones showing an enhanced resistance to killing by UV were examined in detail for their repair capabilities using a variety of techniques. The integrated *denV* plasmid (1–2 copies per cell) improved the UV survival of many though not all clones. The extent of UV resistance correlates well with the ability of the cells to recover DNA synthesis after UV radiation. The UV endonuclease activity of the *denV* gene results in a higher level of DNA breaks and UDS in many of the transfectants. Estimates of the overall excision repair indicates that, for the first 3 h after irradiation, ~4 times more incision events occur in the cells with the *denV* gene (about half of that of a normal human fibroblast). Variation in UV resistance among the transfectants is now being related to the extent of *denV* expression (the number of RNA copies varies widely) and to the rate of repair site completion. In the transfectants about half of the repair sites remain unsealed for abnormally long periods. These studies demonstrate that the product of a prokaryote repair gene can integrate well with the endogenous repair machinery in a human cell and can partially compensate for the lack of UV endonuclease activity in an XPD-like cell.

### Gamma-radiation sensitivity and inhibition of DNA synthesis in human tumour cells *in vitro*

C.N. Parris, C.F. Arlett, A.R. Lehmann, M.L.H. Green & J.R.W. Masters

*Institute of Urology, London WC2H 9AE and MRC Cell Mutation Unit, University of Sussex, Brighton BN1 9OG, UK.*

Cells derived from individuals with the DNA-repair defective syndrome ataxia telangiectasia (A-T) are hypersensitive to gamma-radiation and this is associated with a lack of inhibition of DNA synthesis post-irradiation when compared to normal cells. In this study we show that cells derived from testicular germ cell tumours are more sensitive to *cis*-platin and gamma-irradiation than bladder cancer cells, corresponding with clinical experience. The testicular tumour cells also exhibit a reduced inhibition of DNA synthesis post-irradiation compared with bladder cancer cells (Table). A-T cells are similar to bladder cancer cells in their sensitivity to *cis*-platin, but more sensitive than the normal cells (Table).

| *Cell line* | *Cell type* | *Do (Gy)* | *IC90 cis-platin ($ng\,ml^{-1}$)* | *%DNA synthesis (40 Gy)* |
|---|---|---|---|---|
| AT5BIVA | Ataxia telan. | 1.0 | 220 | 92 |
| MRC5 | Normal | 1.9 | 473 | 40 |
| RT112 | Bladder | 1.7 | 370 | 35 |
| HT1376 | Bladder | 2.1 | 200 | 43 |
| SuSa | Testis | 1.2 | 50 | 62 |
| 833 K | Testis | 1.5 | 60 | 61 |

The sensitivity of testicular tumour cells to *cis*-platin and gamma-irradiation may be analogous to that of AT and related to altered or defective DNA repair.

### Genomic recombination events in a cell line sensitive to difunctional agents

D.A. Lydall & J.J. Roberts

*Department of Molecular Pharmacology, Institute of Cancer Research, Sutton, Surrey SM2 5PX, UK.*

Genetic and biochemical studies in bacteria indicate that excision repair and recombination events are required for the repair of DNA interstrand crosslinks. We have studied a cell line derived from the Walker 256 carcinoma that is sensitive to difunctional but not to monofunctional agents and determined its recombination proficiency in comparison with a derived, resistant, subline (of normal sensitivity).

The plasmid pDR1 which contains two truncated (and therefore nonfunctional) non tandem, but overlapping segments of the *neo* gene separated by a functional transcription unit coding for the *gpt* gene (Subramani & Rubnitz, *Mol. Cell. Biol.*, **5**, 659, 1985). The plasmid pDR1 was transfected into the Walker cells (using the *gpt* gene to select transfectants) and subsequently recombination of the integrated defective *neo* gene segments was assayed by the appearance of G418 resistant cells. The effect of treatment of the cells with *cis*-platin on recombination frequency has also been determined. Recombination between the homologous regions of the segments was confirmed by Southern blot analysis.

### Excision repair of UV damage is insensitive to etoposide

C.S. Downes, A.M. Mullinger & R.T. Johnson

*CRC Mammalian Cell DNA Repair Group, Department of Zoology, Cambridge CB2 3EJ, UK.*

Novobiocin, an inhibitor of DNA topoisomerase II, blocks the excision repair of UV damage, acting at a pre-incision step. A pre-incision topoisomerase action has therefore been postulated as a controlling stage in excision repair. But we have shown (Downes *et al.*, *Carcinogenesis*, **6**, 1343, 1985) that novobiocin also affects mitochondrial structure and ATP metabolism; this action may account for its inhibition of excision repair. We have now investigated the effects of etoposide, another inhibitor of topoisomerase II, on UV repair in human cells. Etoposide is a more specific agent with no side-effects on mitochondria; but at concentrations where its inhibition of topoisomerase II produces DNA strand breaks (and its effects are ultimately toxic), etoposide is without effect on excision repair. UV irradiation does not induce additional strand breaks in the presence of etoposide; nor does etoposide prevent the accumulation of breaks at repair sites in the presence of DNA polymerase inhibitors; nor does it affect religation of repair-induced breaks, or break-induced chromosome decondensation. There is therefore no need to suppose that topoisomerase II is involved at any stage in excision repair.

### Induced mutation frequencies and differential chemosensitivities in human tumour cells *in vitro*

C.N. Parris & J.R.W. Masters

*Institute of Urology, Department of Histopathology, St Paul's Hospital, London WC2H 9AE, UK.*

Drug resistance in tumours may develop as a result of mutations induced by chemotherapy. Testicular germ cell tumours, in contrast to most other types of cancer, are curable even in advanced stages using chemotherapy. We compared spontaneous and induced mutation frequencies (MF) in continuous cell lines derived from two testicular germ cell tumours (833 K, SuSa) and one bladder cancer (RT112). Forward mutations at the hypoxanthine guanine phosphoribosyl transferase locus (HGPRT) were selected following exposure to 10 $\mu g\,ml^{-1}$ 6-thioguanine. Induced mutation frequencies were compared following exposure to equitoxic and equimolar concentrations of ethyl methane sulphonate (EMS). (Table).

| *Cell line* | *Sponstaneous MF/survivor* | *Induced MF at ID50 EMS conc.* | *Induced MF at equimolar EMC conc. (0.6 mg ml$^{-1}$)* |
|---|---|---|---|
| RT112 | $7.5\times10^{-6}$ | $5.6\times10^{-5}$ | $2.4\times10^{-5}$ |
| 833K | $5.3\times10^{-6}$ | $1.2\times10^{-5}$ | $2.5\times10^{-5}$ |
| SuSa | $6.9\times10^{-6}$ | $7.1\times10^{-6}$ | $1.4\times10^{-5}$ |

At equitoxic doses mutations were higher in the bladder than in the testicular cell lines, but were similar at equimolar doses of EMS. Different frequencies of mutation in these cell lines, particularly at equitoxic doses of EMS, may mediate the response of these tumours to chemotherapy.

### Changes in cellular uptake of alkyl-aziridine analogues of the radiosensitizer RSU 1069 as a function of the basicity of the compounds

J.M. Walling, I.J. Stratford & G.E. Adams

*MRC Radiobiology Unit, Chilton Didcot, Oxon OX11 0RD, UK.*

Alkyl aziridine analogues of the hypoxic cell radiosensitizer RSU 1069 that are less toxic *in vivo* have been synthesized. There is a prospect that the therapeutic ratio of some of these analogues will be greater than RSU 1069. In particular, RB 7040, the tetramethyl substituted aziridine, is a more efficient sensitizer *in vitro* than RSU 1069, especially at low concentrations. RSU 1069 and its analogues are weak bases and it is known that such compounds may concentrate intracellularly. We have investigated to what extent variation in drug uptake influences the sensitizing efficiency of RSU 1069 and its analogues. This was done by determining the pH dependence of cellular drug uptake and making comparison with the sensitizing properties of the analogues at extracellular pH values ($pH_e$) in the range 5.4 to 8.4. Three compounds were chosen for study: RSU 1069, RSU 1165 and RB 7040 (values of $pK_a$ of 6.04, 7.38 and 8.45 respectively). Following exposure of V79 cells to these agents for 1 h at room temperature, the ratio of intra- to extra-cellular concentration (Ci/Ce) was near unity at pH 5.4. Increasing $pH_e$ to 8.4 resulted in no change in the ratio Ci/Ce for RSU 1069; in contrast, for RSU 1165 and RB 7040 values of Ci/Ce increased 3 × and 11 × respectively. Radiosensitisation by RSU 1069 was independent of $pH_e$ over the range studied, whereas increasing pH led to an apparent increase in sensitizing efficiency of both RSU 1165 and RB 7040. However, when normalized for difference in drug uptake at the different values of $pH_e$ sensitization was independent of $pH_e$. This study suggests that subtle changes in basicity ($pK_a$) may have potential for therapeutic exploitation on the basis of selective drug uptake, particularly since pH gradients are known to exist across tumours.

### Metastasis and growth factors

### Transfection of metastatic capability with total genomic DNA from metastatic cell lines

A.J. Hayle[1], D.L. Darling[1], P.A. Whittaker[2], K.A. Fleming[1], & D. Tarin[1]

[1]*Nuffield Department of Pathology, Nuffield Department of Pathology, John Radcliffe Hospital, Oxford OX3 9DU and* [2]*Department of Biochemistry, University of Oxford, South Parks Road, Oxford OX1 3QU, UK.*

Transfer of total genomic DNA from a human malignant melanoma cell line, which is capable of metastasis in the nude mouse, to a tumorigenic but nonmetastatic mouse cell line resulted in the latter acquiring the ability to colonise distant organs in a substantial proportion of animals inoculated i.v. with the cells. The results also showed that these cells could form colonies in extrapulmonary sites and Southern blot analysis indicated the presence of human DNA. Additional studies involving transfection of DNA from a highly metastatic mouse cell line (of histiocytic origin) into a very weakly metastatic mouse mammary carcinoma cell line resulted in marked augmentation of spontaneous metastasis from tumours formed by the recipient cells in the mammary fat pad. Transfections were performed using the calcium phosphate precipitation method and incorporated a dominant co-selectable marker (the gene for amino-glycoside transferase, which confers resistance to neomycin). The survival of cell clones in neomycin-containing medium provided further evidence of incorporation of exogenous DNA and these clones were pooled before injection to increase the numbers that could be screened for metastatic behaviour. The results indicate that components of the metastatic phenotype are heritable, highly conserved in evolution and can be dominantly conferred on previously non-metastatic tumour cells by transfer of genomic DNA. These findings open opportunities for the isolation of genes involved in metastatic behaviour and for studies of their regulation.

**Expression of oncogenes in a rat glioma cell line induced transplacentally with ethylnitrosourea**

P.C. Rumsby, L.J. Green, V.T. Chow & J.P. Roscoe

*Department of Cell Pathology, School of Pathology, Middlesex Hospital Medical School, London W1P 7LD, UK.*

The induction of rat brain gliomas by transplacental treatment with ethylnitrosourea (ENU) has proved to be a good model for studying the development of tumours (Roscoe & Claisse, *Nature*, **262,** 314, 1976). We have looked at oncogene expression in the cloned glioma cell line, A15A5 and a cloned line from a normal adult rat brain, ARBO C9, using Northern blot analysis.

Hybridisation with a human genomic N-*ras* probe revealed increased expression of 2 species of mRNA, 3.8 and 3.4kb, in the glioma cells. There was no expression of Ki- or Ha-*ras* nor in this system, of c-*cis* or epidermal growth factor receptor-related mRNAs which have been found in several human neural tumours.

Hybridisation with a v-*myc* probe gave bands at 2.4 and 2.7kb and less distinct ones at 3.8 and 1.8kb. A human c-*myc* probe hybridised weakly to the 2.4kb species. The normal ARBO C9 cell line had mainly the 2.7kb species and A15A5 the 2.4kb mRNA. There was no hybridisation with a human N-*myc* probe.

The *neu* oncogene has been implicated as the activating oncogene in some other ENU-induced glioblastomas (Bargmann *et al. Cell*, **45,** 649, 1986). There was no difference in the expression of a 5kb *neu* mRNA in the ARBO C9 and A15A5 cell lines suggesting that in this glioma any activation by *neu* is by small qualitative change in the gene rather than by alteration in the level of *neu* expression.

**The clinical significance of c-*myc* oncogene expression in colorectal cancer**

D.J. Jones[1,2], A.K. Ghosh[2], M. Moore[2] & P.F. Schofield[1]

[1]*Department of Surgery and* [2]*Paterson Institute for Cancer Research, Christie Hospital and Holt Radium Institute, Manchester M20 9BX, UK.*

The c-*myc* oncogene is involved in the regulation of cell proliferation; elevated levels of c-*myc* mRNA and its product p62c-*myc* have been detected in solid tumours and cell lines using molecular biological techniques. Oncogene expression has recently been assessed using monoclonal antibodies to oncoproteins generated against synthetic peptides. We studied c-*myc* expression in colorectal carcinomas from 100 patients, prospectively followed for 3 years, using Myc 1-6E10 which recognizes p62c-*myc* in paraffin-embedded material (Stewart *et al., Br. J. Cancer,* **53,** 1, 1986). Tissue sections were stained using an immunoperoxidase technique, and assessed independently by two observers. Paradoxically staining was predominantly cytoplasmic despite the reported nuclear location of p62c-*myc* (Evan & Hancock, *Cell,* **43,** 253, 1985). In normal mucosa, maturing crypt cells and surface epithelial cells were weakly positive. All carcinomas stained positively but with varying intensity. However, though distinctive staining patterns could be distinguished for both normal mucosa and for carcinomas, there was also considerable background staining of stromal elements. There was no significant relationship between staining intensity and conventional pathological features or prognosis. These results suggest that c-*myc* expression is a feature of normal maturing epithelial cells and that expression is increased in the majority of carcinomas and may be related to malignant transformation, but not to clinical behaviour. Paradoxical cytoplasmic and stromal staining may be due to fixation artefact. Alternatively in tissue sections Myc 1-6E10 may cross-react with proteins other than p62c-*myc* (cf. Evan *et al., Mol. Cell. Biol.,* **5,** 3610, 1985). Data obtained on histological sections using this antibody should therefore be interpreted with caution.

**Differential responses to activators of cAMP in metastatic human melanoma variants**

E.J. Ormerod & I.R. Hart

*ICRF Laboratories, Lincoln's Inn Fields, London, WC2A 3PX, UK.*

Recently we described the derivation of a series of human melanoma cell lines exhibiting differential lung colonising capacity in nude mice. (Ormerod *et al., Cancer Res.,* **46,** 884, 1986.) In the present study we have examined the response of these variants to activators of cyclic adenosine 3-5-monophosphate (cAMP).

The response of the low-metastatic parental DX3 line and the high-metastatic LT5.1 variants to cholera toxin, forskolin, theopylline and melanocyte stimulating hormone was assessed in terms of *in vitro* growth rates, morphology, plating efficiencies and cAMP levels. Differences were found between the lines in the way that they responded to $10^{-9}$ M cholera toxin such that the *in vitro* growth rate of the DX3 line was hardly affected by the presence of this agent whereas cell division of LT5.1 variants ceased completely within 5–7 days although cAMP levels were elevated 5–10-fold in both lines. Pretreatment of LT5.1 cells prior to s.c. or i.v. injection into athymic nude mice retarded tumour growth and reduced lung nodules 3–5 fold respectively. Currently we are determining whether *in vivo* treatments can bring about similar reductions in metastatic burdens.

That variants of a human melanoma cell line show differential responses to cholera toxin might reflect differences in cAMP metabolism and its growth regulatory function in high and low metastatic lines.

**Regulation of proliferative and metastatic activity of a murine tumour by 12-0-tetradecanoylphorbol 13-acetate (TPA)**

N. Goode & I.R. Hart

*ICRF Laboratories, Lincoln's Inn Fields, London WC2A 3PX, UK.*

Cell division of the macrophage tumour M5076 is inhibited by a variety of agents known to induce differentiation in other tumours (Talmadge *et al.*, *Cancer Res.*, **42,** 1850, 1982). The purpose of this study was to investigate the relationship between this response to one such agent, TPA, and the *in vivo* behaviour of the cells.

A TPA resistant M5076 line ($TPA^R$) was derived by culturing cells continually in $500\,ng\,ml^{-1}$ TPA. Exposure of the wild-type cells to $50\,ng\,ml^{-1}$ TPA for 24 h inhibited cellular proliferation by >95% as determined by [$^3$H]-thymidine ($^3$H-TdR) incorporation, whereas $TPA^R$ cells were still able to grow at $1\,\mu g\,ml^{-1}$. The doubling time of $TPA^R$ cells in normal growth medium was 26 h compared with 32 h for the parental cell line. Experimental metastatic activity was determined by injecting $5\times10^4$ viable cells i.v. into groups of syngeneic C57 mice (10 mice/group) and counting liver tumour nodules 3 weeks later. M5076 cells produced a median of 190 hepatic nodules (range 148–200). While pre-treatment of the cells with $50\,ng\,ml^{-1}$ TPA for 24 h resulted in a median of 9 hepatic nodules (range 0–31). Numbers of liver tumours resulting from $TPA^R$ cells were not reduced (median 142, range 22–194) but their smaller size was reflected by the lower liver weight (mean of 1.2 g *vs.* 2.3 g for livers from mice receiving M5076 cells). Similarly, the s.c. injection of $10^5$ viable cells of M5076 produced palpable tumours within 2 weeks and a time to tumour weight of 0.7 g of 4 weeks whereas tumours resulting from $TPA^R$ cells were palpable at 3–4 weeks after injection and attained a weight of 0.4 g 7 weeks after s.c. injection. The basis of this reduction of malignant capacity of $TPA^R$ cells is currently under investigation.

**Monoclonal antibodies to oncoproteins do not distinguish between normal liver, hepatocellular carcinoma and preneoplastic liver lesions in rats**

M.J. Embleton & P.C. Butler

*Cancer Research Campaign Laboratories, University of Nottingham, Nottingham NG7 2RD, UK.*

Fischer F344 rats were treated with a diet containing 0.06% 2-acetyl-aminofluorene (AAF), administered as a series of 2 week cycles separated by periods of carcinogen-free diet. During 4 such cycles they developed various liver lesions including cirrhosis, oval cell proliferation, foci of cells containing raised levels of gamma-glutamyl transpeptidase and hyperplastic nodules. Nine months after commencing AAF diet they began to develop primary hepatocellular carcinomas. Specimens of normal liver, tumour and preneoplastic liver were frozen for histological study, and both plasma membrane and total soluble extracts were prepared from these tissues. A panel of murine monoclonal antibodies to proteins coded by the *myc*, *myb*, *ras*, *sis*, *erb*-B and *src* oncogenes were tested for reactivity with cryostat sections of normal liver, tumours and preneoplastic liver by immunoperoxidase staining. Reactivity with tissue extracts was tested by radio-immunoassay using $^{125}$I-labelled anti-mouse Ig as second antibody.

The anti-oncoprotein antibodies produced variable staining of normal liver sections, anti-*myc* and anti-*myb* products reacting more strongly than the others. Hepatocellular carcinoma tissue and livers containing identifiable premalignant lesions showed little difference from normal liver, other than increased cellular heterogeneity within some tumours. Radioimmunoassays indicated reactivity of all 6 antibodies with extracts of normal, preneoplastic and neoplastic livers. There was a slight increase in reactivity of anti-*myc* and anti-*myb* products with some tumour extracts, but no major differences. It was concluded that conversion from the normal to the malignant phenotype in rat liver was not accompanied by gross changes in expression of oncogene products detected by these antibodies.

**Levels of soluble class I and class I-like molecules in sera from mice bearing metastasising and non-metastasising tumours**

M. Blackmore, S. Thompson & G.A. Turner

*Department of Clinical Biochemistry, The Medical School, Framlington Place, Newcastle upon Tyne, NE2 4HH, UK.*

Recently discovered truncated MHC class I molecules have been implicated in the host response to cancer (Festenstein & Garrido, *Nature*, **332,** 502, 1986), and may be acting as serum blocking factors that suppress the immune response. We have developed a method to measure class I and class I-like molecules in serum in order to investigate the possible relationship between the blood levels of these substances and tumour growth and metastasis. Class I material was partially purified from serum using lentil lectin coupled to Sepharose, this extract was labelled with $I^{125}$ and subjected to immunoprecipitation using various antibodies. Two groups of antibody have been used; group A recognised classical K and D determinants, group B recognised molecules that were similar to class I MHC but were expressed on embryonal carcinomas. The immunoprecipitates were analysed using 1D-SDS/PAGE electrophoresis followed by autoradiography. Using group A antibodies, molecules of 39–42 K daltons were identified in serum from healthy mice and animals bearing different types of tumour. The levels of these molecules were elevated in the tumour bearers and also appeared to be more marked if the tumours metastasised. Using group B antibodies, extra molecules at 34 and 36 K daltons were seen. The 36 K molecule appeared to be associated with liver metastasis, whereas the 34 K molecule was elevated in certain tumour-bearers. These results suggest that increased release of class I and class I-like molecules is occurring into the blood of tumour bearing mice; this process could have important consequences for the understanding and treatment of cancer.

**Somatomedin-C (Sm-C)/insulin-like growth factor 1 is a mitogen for human small cell lung cancer (SCLC)**

V. Macaulay, G.P. Joshi, J.D. Teale, M. Everard, I.E. Smith & J.L. Millar

*Department of Medicine, Institute of Cancer Research, Sutton, Surrey and Department of Clinical Biochemistry, St Luke's Hospital, Guildford, Surrey, UK.*

Rapid proliferation in SCLC may relate to production of autocrine growth factor(s). Bombesin-like immunoreactivity (BLI) is secreted by and mitogenic to classic SCLC but not the faster growing variant lines. Conditioned medium (CM) from a classic SCLC line, HC12, underwent pressure filtration yielding a high molecular weight (> 10,000) concentrate depleted of BLI. This preparation enhanced $^3$H-thymidine uptake in 2 classic lines HC12, and HX149 over PBS-supplemented controls; it was found to contain immunoreactive Sm-C ($200\,ng\,ml^{-1}$), prompting a survey of human lung cell lines and tissues. Radioimmunoassay revealed Sm-C (ng mg$^{-1}$ protein) in all of 3 classic SCLC lines (HC12 cells 266.7, CM 70.0; HX149 211.1, 60.0; NCI-H69 150.0, 273.3), CM of one variant, ICR-SC17 (4.4, cells 0) and also in a large cell anaplastic lung line, HX147 (cells 242.5, CM 6.8); a myeloma line (RPMI 8226) was negative. Sm-C was present in all of 4 SCLC tissue samples (mean 44.7±6.8; non-tumoural lung 20.0), including the biopsy of origin of ICR-SC17. Large cell anaplastic tissue was positive (40.0) as were 3 of 4 primary lung squamous carcinomas (76.8±25.2) but 0 of 2 adenocarcinomas. We assessed the effect of recombinant Sm-C (0.1–500 ng ml$^{-1}$) on $^{3}$H-thymidine uptake: there was a clear response in 2 of 3 classic SCLC lines and in one variant: maximal effects were seen in HC12 at 100 ng ml$^{-1}$ (uptake 214% over control, $P<0.01$), in HX149 at 300 ng ml$^{-1}$ (193%, $P<0.01$), in NCI-H69 at 100 ng ml$^{-1}$ (137%, NS) and in ICR-SC17 at 200 ng ml$^{-1}$ (207%, $P<0.01$). There was a similar effect in HX147 (242% at 100 ng ml$^{-1}$, $P<0.01$) but no response in RPMI 8226. Sm-C may function as an autocrine growth factor in SCLC. We plan to assess additional lung lines, and to investigate mechanisms of mitogenicity in SCLC.

### Comparison of the effects of a phorbol ester and mezerein on the growth of A549 human carcinoma cells

I. Dale & A. Gescher

*CRC Experimental Chemotherapy Research Group, Pharmaceutical Sciences Institute, Aston University, Birmingham B4 7ET, UK.*

The diterpene 12-0-tetradecanoylphorbol-13-acetate (TPA) exerts a wide range of effects on biological systems, ranging from tumour promotion to growth inhibition, the latter being sometimes the result of the induction of terminal differentiation. As part of an attempt (i) to elucidate the mechanism by which TPA exerts growth inhibition in the human A549 lung carcinoma cell line and (ii) to characterise structural features which confer growth-inhibitory potential on the TPA molecule we investigated the effects of a structurally related tumour promoter, the resiniferonol derivative mezerein (MEZ) on these cells. Cells were incubated with MEZ or TPA and counted after 5 days. Additionally incorporation of $^{3}$H-thymidine into cells was assessed 24 h after addition of MEZ or TPA to the incubate. Whereas the concentration of TPA which inhibited growth or thymidine incorporation by 50% ($IC_{50}$) was 0.1 nM the $IC_{50}$ for MEZ was 5 nM. Maximal inhibition of thymidine incorporation (80%) was achieved after 12 h incubation of cells with either $10^{-7}$ M MEZ or $10^{-8}$ M TPA. After an incubation period of 5–6 days, in which the growth of the cells exposed to either MEZ or TPA was completely arrested, cells began to grow again in the presence of either tumour promoter at a rate similar to that of control cells. However, on removal of the promoters the cells regained their sensitivity towards the growth inhibitory potential of MEZ or TPA. In conclusion, it appears that the growth-inhibitory property of phorbol esters is also a feature of other plant-derived tumour promoters. Nevertheless TPA is a significantly more potent growth inhibitor in A549 cells than is MEZ.

### Epidermal growth factor receptors in colonic adenocarcinoma

M. Moorghen[1], K.J. Finney[1], P.G. Ince[1], J.P. Sunter[1], A.L. Harris[2] & A.J. Watson[1]

[1]*Departments of Pathology and* [2]*Clinical Oncology, University of Newcastle upon Tyne, UK.*

Epidermal growth factor receptors (EGFr) have been demonstrated in a number of normal and neoplastic human tissues. In this study, tissues obtained from colonic carcinomas and non-neoplastic colonic mucosae were investigated for the presence of EGF receptors. Radio-ligand binding assays using I-125 failed to show the presence of EGF receptors in three tumours and their corresponding normal mucosae. Immunoperoxidase studies, using EGF-R1 antibody, performed on 8 cases showed positive staining of both tumour and mucosa in 3 cases and positive staining of the mucosa only in 2 cases.

These preliminary findings suggest that EGF receptors are present at a relatively low frequency in human colonic tissues. This contrasts with the findings of other workers who have shown EGFr to be absent from human colon. In rat colonic mucosa, EGF has been shown to act as a trophic agent in an organ culture system, thus implying the presence of EGFr.

Further experiments are being performed on larger numbers to investigate the presence of EGFr and the effects of EGF.

### Epidermal growth factor receptors in human prostate cancer

S.Q. Maddy, G.D. Chisholm & F.K. Habib

*Department of Surgery, University of Edinburgh Medical School, Teviot Place, Edinburgh EH8 9AG, UK.*

Epidermal growth factors (EGF) are powerful mitogens which stimulate cell division by binding to EGF-receptors on cell surface membranes. Although EGF receptors have been measured in benign prostatic hyperplasia (BPH) no one has, so far, investigated their presence in cancer of the prostate (CaP). Characterisation of EGF-receptors in CaP was therefore carried out and the impact of cell differentiation on receptor levels, investigated. In common with the BPH data our ligand exchange assay on cancerous tissue demonstrate the presence of two classes of receptors both of high affinity ($10^{-9}$ M) which are specific for EGF and are not displaced by other growth factors. However, unlike BPH tissue, the concentrations of EGF receptors in CaP were significantly lower ($P<0.05$) and this seemed to correlate with the degree of differentiation of the tumour: Well differentiated cancer tissues exhibited high concentrations of the receptors whereas in poorly differentiated tumours the receptors were absent.

Immunocytochemical assays by means of an indirect immunoperoxidase technique employing a murine monoclonal antibody (donated by Dr M. Waterfield) confirmed the data obtained by biochemical methods and revealed a positive correlation between $I^{125}$-labelled EGF binding and the intensity of staining. Furthermore the staining by the antibody was confined only to the basal layers of epithelial cells whilst the adjacent stromal sections remained clear. The long term implications of these findings are not evident though obviously our data suggest that the absence of EGF receptors in cancer might be in some way related to tumour development.

### An epidermal growth factor receptor ($EGF_r$) in human bladder cancer

K. Smith, J.A. Fennelly, D.E. Neal & A.L. Harris

*Cancer Research Unit, University of Newcastle upon Tyne, Royal Victoria Infirmary, Newcastle upon Tyne NE1 4LP, UK.*

We have assayed EGFr in bladder cancers to see if the presence of the receptor is related to the degree of tumour invasion. Cell membranes and cytosols were prepared by differential centrifugation and EGFr binding was assayed using $^{125}$I-labelled mouse EGF. The binding of EGF to EGFr was linear with respect to protein concentrations and was complete within 30 min at 26°C. The rate constants of association and dissociation were $0.903 \times 10^{9}$ M$^{-1}$ min$^{-1}$ and 0.05 min$^{-1}$ respectively at 26°C. The binding was also specific to EGF. EGFr cross-linked to EGF could be visualised in 2 distinct bands (mol. wt. = 120,000 and 150,000) on fluorographs and in the presence of unlabelled EGF the bands were not seen. Autophosphorylation of EGFr was enhanced in the presence of EGF and when exogenous $Ca^{++}$ was added, the intensity of the 120,000 mol. wt. band was increased. EDTA inhibited phosphorylation of both proteins. Using Scatchard analysis, the number of binding sites ($B_{max}$) and the dissociation constant (Kd) were assayed in 37 bladder cancers. 15 patients had superficial transitional cell carcinoma and 22 patients had invasive transitional cell carcinoma. 6 of the 15 superficial tumours (40.0%) contained EGFr ($B_{max}$ = 8.5 – 32.4 fmol mg$^{-1}$ protein, mean of 14.6 ± 3.6 s.e.). The Kd values were $0.23 - 1.45 \times 10^{-9}$ MM mean = 0.90 ± 0.23 s.e. 16 of the 22 invasive tumours (72.7%) contained EGFr ($B_{max}$ = 8.9 – 1020 fmol mg$^{-1}$ protein, mean = 141.7 ± 75.5 s.e.). The Kd values were 0.24–$2.38 \times 10^{-9}$ M, mean = $0.90 \pm 0.15 \times 10^{-9}$ M s.e. The difference in the amount of receptor between invasive and superficial tumours was significant ($P < 0.01$ Mann–Whitney Test). Using radioimmunoassay and radioreceptor assays, we detected ng amounts of EGF and EGF-like peptides in bladder cytosols. The finding of a higher EGFr concentration in more invasive tumours may suggest that EGFr expression is related to tumour progression.

### Multidrug resistance and anthracyclines

### Inherent adriamycin resistance in a murine tumour line. I: Circumvention with verapamil and norverapamil

S. Merry, D. Kerr, P. Flanigan, R. Milroy, R.I. Freshney & S.B. Kaye

*CRC Department of Medical Oncology, University of Glasgow, Glasgow, UK.*

The calcium antagonist verapamil (VPM) has been consistently shown to circumvent resistance to Adriamycin (ADR) in a number of experimental tumour models *in vitro* at concentrations of 5–10 μM. An ongoing randomised clinical study in small cell lung cancer indicates that plasma levels of up to 3 μM VPM may be obtained without major toxicity. The major metabolite of VPM is norverapamil (NVPM) which we have found to be present in equimolar concentrations in plasma. If NVPM were equally active in the context of drug resistance the clinical potential of VPM might be significantly improved. We have investigated the ability of VPM and NVPM to circumvent inherent resistance to ADR in the murine tumour line MOG-XMT1 *in vitro* using (a) monolayer cloning and (b) MTT reduction as end points. Cloning was carried out with continuous exposure to drugs and colonies >16 cells were scored after 10 days. In the MTT assay exponentially-growing cells were treated with ADR ± VPM or NVPM for 24 h and viability was assessed after a recovery period of 96 h in the absence of drugs. The $ID_{50}$ for ADR as measured by cloning (34 ± 5 nM; $n = 9$) was reduced 18-fold with 6.6 μM VPM and 5-fold by 6.6 μM NVPM. The $ID_{50}$ for ADR in the MTT assay (126 ± 27 nM; $n = 3$) was reduced 5-fold by either 6.6 μM VPM or 6.6 μM NVPM. These data indicate that NVPM may have similar activity to VPM in overcoming drug resistance. Our preliminary data indicate that the effects of VPM and NVPM are additive. Further studies to confirm this observation are underway.

### Cross-resistance patterns and protein alterations in drug resistant sublines of EMT6 mouse tumour cells

P.R. Twentyman[1], G.L.E. Koch[2], J.G. Reeve[1], N.E. Fox[1] & K.A. Wright[1]

[1]*MRC Clinical Oncology and Radiotherapeutics Unit and* [2]*Laboratory of Molecular Biology, Cambridge CB2 2QH, UK.*

A range of drug-resistant sublines of the EMT6 mouse tumour cell line has been derived by continuous growth in increasing concentrations of cytotoxic drugs. Sublines are now growing in adriamycin (ADM) (10 μg ml$^{-1}$) vincristine (VCR) (10 μg ml$^{-1}$), colchicine (COL) (2 μg ml$^{-1}$), Ro 31-1215 (1215) (0.1 μg ml$^{-1}$) and methotrexate (MTX) (10 μg ml$^{-1}$). Cross-resistance patterns have been studied using a tetrazolium (MTT) colorimetric assay to measure growth inhibition during continuous drug exposure. Cells made resistant to ADM, VCR, COL or 1215 showed cross-resistance to the other drugs in this group. The extent of resistance to 1215 (or to aclacinomycin A) was, however, considerably lower (×5–10) than it was to the other 3 agents (×20–100). These cells were all also highly resistant to VP16 (×20–50) and to mitozantrone (×50–300) but not to melphalan, CCNU or MTX. However, cells made resistant to MTX also showed a small degree of resistance to ADM, VCR and COL. These cells could be sensitised to ADM, but not to MTX, using the calcium transport blocker, verapamil. A monospecific antibody to the $CP_{22}$ cytosolic calcium-binding protein (Koch *et al*, *FEBS Letters*, **195**, 275, 1986) and immunoblotting have been used to quantify the presence of this protein in the various cells. Cellular content of $CP_{22}$ increased progressively during the development of ADM resistance but dropped suddenly at the highest level of resistance. Increased levels of $CP_{22}$ were seen in some but not all of the resistant lines and the amount did not predict for the extent of resistance to any given drug. Further studies using this antibody and also antibodies to the P-170 glycoprotein are in progress.

### Evidence that multidrug resistance in Chinese hamster ovary cells is associated with alterations in the endoplasmic reticulum

J.G. Reeve[1], G. Koch[2] & P.R. Twentyman[1]

[1]*MRC Clinical Oncology and Radiotherapeutics Unit and* [2]*Laboratory of Molecular Biology, MRC Centre, Cambridge CB2 2QH, UK.*

Analysis of microsomal membrane proteins from drug resistant ($CH^{R}C^{5}$) and drug sensitive (AUXBI) Chinese hamster ovary (CHO) cells by SDS gel electrophoresis revealed, in addition to altered P-glycoprotein expression, increased levels of a 92 kDa protein in the fraction from $CH^{R}C^{5}$ cells. The protein was identified as the major glycoprotein, endoplasmin, by its reactivity with a monospecific affinity-purified antibody to endoplasmin and by its calcium-binding properties. Endoplasmin is localised to the endoplasmic reticulum (ER) and is the same as one of the major stress-related proteins GRP. Immunoblotting analyses showed that overall expression of endoplasmin was not altered but that the higher levels in $CH^{R}C^{5}$ microsomal membranes resulted from increased retention of the glycoprotein by the microsomal fraction during subcellular fractionation. These observations indicate that structural changes in the ER which alter the orientation of vesiculation upon disruption occur in drug resistant CHO cells and imply that membrane changes associated with the resistant phenotype are not confined to the plasma membrane.

### Characterisation of a CHO cell line hypersensitive to topoisomerase II inhibitors

C. Robson, S. Davies, P. Hoban, S. Davies, A. Harris & I. Hickson

*Cancer Research Unit, University of Newcastle upon Tyne, Royal Victoria Infirmary, Newcastle upon Tyne NE1 4LP, UK.*

We have isolated a Chinese hamster ovary (CHO) cell line, designated ADR-1, which exhibits hypersensitivity to a range of drugs which are thought to inhibit the action of the enzyme topoisomerase II. These include anthracyclines, other classes of intercalating agents, and the epipodophyllotoxin VP16 (etoposide). No significant sensitivity to radiation, or to mono- and bi-functional alkylating agents, is seen, although mild cross-sensitivity to the radiomimetic agent bleomycin is observed.

We have monitored the level of DNA strand breaks induced by topoisomerase II inhibitors in ADR-1 cells using alkaline elution. At equimolar adriamycin (doxorubicin) doses, more protein-associated DNA strand breaks are induced in ADR-1 cells than in wild-type cells. This enhanced level of drug-induced strand breaks is not a function of increased drug uptake as both lines accumulate similar levels of radiolabelled daunomycin. Both the rate of repair of strand breaks and the final percentage of strand breaks rejoined is equivalent in the 2 cell lines. These results are consistent with there being an enhancement in the level of topoisomerase II-dependent DNA breakage in ADR-1 cells following exposure to topoisomerase II inhibitors.

Topoisomerase II enzyme partially purified from ADR-1 cells shows altered activity in assays of both covalent binding to duplex DNA and decatenation of kinetoplast DNA.

### Hormonal enhancement of etoposide-induced DNA damage and cytotoxicity in a breast cancer cell line

R.J. Epstein, P.J. Smith, J.V. Watson & N.M. Bleehen

*MRC Unit, Clinical Oncology and Radiotherapeutics, MRC Centre, Cambridge CB2 2QH, UK.*

T47D human breast cancer cells were maintained in charcoal-stripped medium and exposed to oestradiol at various intervals prior to administration of etoposide (VP-16), a topoisomerase-II-interactive drug. DNA strand-breakage measured by alkaline unwinding was 40% above control levels after 4h of oestrogen exposure, and 85% above controls after 24h exposure. Oestrogen pre-treatment did not increase DNA damage induced by X-rays or bleomycin; however, administration of another topoisomerase-II-interactive agent, mitoxantrone, led to a similar oestrogen-induced increase in damage to that witnessed with etoposide. No change in rate of DNA repair could be detected following either X-irradiation or etoposide treatment of cells pretreated with oestrogen when compared with controls. The degree of excess damage associated with oestrogen pre-treatment was reduced by either 4-hydroxy-tamoxifen or novobiocin co-administration, which is consistent with an increase in topoisomerase II levels accompanying oestrogen-induced cell activation. Moreover, the excess damage documented on alkaline unwinding could not be detected on nucleoid sedimentation, suggesting further that the differential strand-breakage is protein-(topoisomerase II)-associated. Dose-dependent inhibition of cell growth, clonogenicity and cell-cycle traverse varied directly with the extent of DNA damage induced in both oestrogen-treated and control cells, and analysis of these data suggest the existence of a cellular subpopulation in which topoisomerase-II-induced DNA damage may occur preferentially following cell activation by oestrogen. These findings raise the possibility that novel therapeutic strategies for established human breast cancer may be feasible using topoisomerase-II-interactive drugs.

### Comparative biodistribution of daunomycin-HSA-monoclonal antibody conjugates with different chemical linkages

Y. Ogunmuyiwa, J.A. Clegg, M.V. Pimm, M.R. Price & R.W. Baldwin

*Cancer Research Campaign Laboratories, University of Nottingham, Nottingham NG7 2RD, UK.*

The therapeutic effectiveness of drug-monoclonal antibody conjugates will depend partly on their biodistribution since if they are rapidly eliminated they are unlikely to reach the tumour target. In this study daunomycin-carrier-monoclonal antibody conjugates have been constructed with different chemical linkages and their biodistributions assessed.

Initially daunomycin (daun) was conjugated to human serum albumin (HSA) to molar substitution ratios of up to 20:1 either by reaction with 14-bromo-daunomycin to give a C-N bond between the 14-C of the drug and protein amino groups, or by reaction of succinylated HSA with the 14-bromo-daunomycin to give a succinyl ester linkage. Subsequently these dauno-HSA conjugates were linked to the 791T/36 monoclonal antibody via a thioether linkage at a 1:1 HSA:antibody molar ratio. For biodistribution studies the protein moieties of the dauno-HSA or dauno-HSA-791T/36 conjugates were labelled with radioiodine ($^{125}$I or $^{131}$I) and blood survival and biodistribution in mice compared with radio-iodine labelled HSA, succinylated HSA HSA-791T/36 and 791T/36 alone. With the 14-C linked conjugates, blood survival of dauno-HSA, and dauno-HSA-791T/36 were similar to those of HSA or antibody. Blood contained more radiolabel than any other organ, the survivals at 24h being 27% for 791T/36 antibody, 19.1% for HSA and 20.4% for dauno-HSA-791T/36. In contrast, with the succinyl ester linkage, dauno-HSA and dauno-HSA-791T/36 were rapidly cleared from the blood, particularly to liver and spleen so that blood survivals were $<1\%$ at 24h. Succinylated HSA without drug was similarly cleared, with survival $<1\%$ after 24h.

These studies have demonstrated that the biodistribution of dauno-carrier-antibody conjugate is markedly dependent upon their chemical linkages. This is probably related at least in part to the overall charge on the molecule. The conjugates with the 14-C linkage would have a net positive charge and these survived, whereas succinyl ester linked conjugates would have a net negative charge which encourages clearance by phagocytic cells.

### Anthracycline structure-activity relationships in human lung spheroids

D.J. Kerr, T.E. Weldon & S.B. Kaye

*Department of Medical Oncology, Glasgow University and Radiobiology Group, Belvidere Hospital, Glasgow, UK.*

Penetration barriers have been demonstrated for adriamycin in multi-cellular tumour spheroids. Adriamycin enters the cell by diffusion of the electroneutral molecule through the lipid domain of the cell membrane and we have hypothesised that lipophlic anthracycline analogues would tend to diffuse further into solid tumour masses, with improved cytotoxic efficacy. The activities of adriamycin, 4′-deoxydoxorubicin, Daunorubicin, 4-demethoxydaunorubicin, 4′-deoxy, 4′-iododoxorubicin have been assessed by clonogenic assay (L-DAN line) in monolayers and growth delay in L-DAN spheroids. Intracellular anthracycline levels were measured after exposure to each of these drugs (varying concentrations for 1 h) by an HPLC assay. These is a significant correlation ($P<0.05$) between log oil-water partition co-efficient and log intracellular drug levels and log spheroid growth delay but not with monolayer $ID_{90}$.

| *Drug* | *Mono-layer $ID_{90}$ ($\mu g\,ml^{-1}$)* | *Conc. doubling growth delay ($\mu g\,ml^{-1}$)* | *Oil-water (coefficient)* | *Intra-cellular drug conc. (ng $10^{-5}$ cells)* |
|---|---|---|---|---|
| Adriamycin | 2.2 | 10 | 6.3 | 14 |
| 4′-Deoxy-doxorubicin | 2.3 | 0.6 | 15 | 154 |
| Daunorubicin | 1 | 0.68 | 17.8 | 170 |
| 4-Demethoxy-daunorubicin | 0.1 | 0.05 | 32.3 | 400 |
| 4′-Deoxy-4′-Iodo-doxorubicin | 0.007 | 0.004 | 126 | 1961 |

### Protein microspheres as carriers for adriamycin: Comparison of potency and drug-loading characteristics

N. Willmott[1], Y. Chen[1], J. Cummings[2] & A.T. Florence[1]

[1]*Department of Pharmacy, University of Strathclyde, Glasgow and* [2]*Department of Medical Oncology, University of Glasgow, Glasgow, UK.*

Since the initial descriptions of the preparation of protein microspheres (MS) albumin has been most frequently used as drug carrier. However, whether its properties are optimal for the role of carrier matrix is unknown. Consequently, we have prepared, by glutaraldehyde stabilisation, adriamycin (Adx)-loaded protein MS using albumin ($9\pm2.8\,\mu g$ Adx $mg^{-1}$; $n=5$) and casein ($3.1\pm1\,\mu g$ Adx $mg^{-1}$; $n=6$) as matrix material. We have also developed systems in which Adx is incorporated into albumin MS via a non-covalent complex with polyaspartic acid ($30.5\pm5.8\,\mu g$ Adx $mg^{-1}$; $n=4$). Incorporated drug potency was assessed following direct injection into SC growths of the non-immunogenic rat mammary carcinoma Sp107. It was observed that both albumin ($85\,\mu g$ Adx) and casein ($11\,\mu g$ Adx) systems exerted marked inhibition of tumour growth (GD = 18.6 and 20.7 days respectively), whereas albumin/polyaspartic acid MS ($121\,\mu g$ Adx) were less active (GD = 4.5 days). To explain these results the state of Adx in microspherical form was examined by HPLC with multidiode array spectrophotometric detection. This technique revealed that, in addition to pure Adx, a chromatographically distinct Adx-derived species was present in albumin and casein MS. In albumin/polyaspartic acid MS Adx was present only in native form, possibly due to its inability to react with glutaraldehyde when complexed with polyaspartic acid. These studies reveal the protean nature of Adx incorporated into albumin and casein microspheres and are consistent with the presence of an Adx conjugate (probably with protein), the presence of which correlates with potency in this system.

### Tumour markers

### Cellular and serum carcinoembryonic antigen levels in colorectal cancer

L.G. Durrant[1], K.C. Ballantyne[2], R.A. Robins[1], D. Henson[3], R.W. Baldwin[1] & J.D. Hardcastle[2]

[1]*Cancer Research Campaign Laboratories and Departments of* [2]*Surgery and* [3]*Biochemistry, University of Nottingham, Nottingham NG7 2RD, UK.*

Carcinoembryonic antigen (CEA) is expressed by ~80% of colorectal cancers (CRC). However serum CEA levels are raised in under 50% of patients with primary CRC. Preoperative serum CEA in 66 patients with primary CRC (42 men, 24 women) was compared with a quantitative assessment of tumour cell CEA expression, histological tumour grade, pathological stage and maximum tumour diameter. Eight tumours were well differentiated, 46 moderately and 12 poorly differentiated. Twelve were Stage A tumours, 26 Stage B, 16 Stage C and 12 Stage D. Serum CEA was measured by radioimmunoassay. Tumour cell CEA expression was measured by flow cytometry on disaggregated primary tumour cells using 3 anti-CEA antibodies and the results expressed in fluorescence units (FLU) for each tumour. Serum CEA levels were elevated ($>10\,mmol\,l^{-1}$) in 23 patients (34%). However, 92% of tumours expressed CEA at a cellular level. Mean tumour fluorescent ± s.e. (Fl U): anti-CEA = $848\pm85.5$ normal mouse immunoglobulin (control) $46.5\pm3.9$ ($t=9.4$; $P<0.0001$).

Serum CEA levels did correlate with pathological stage and maximum tumour diameter ($P<0.001$). Since most colorectal cancers express CEA at a cellular level elevated serum levels of CEA are a reflection of tumour load.

### Mapping of monoclonal antibody-defined epitopes on carcinoembryonic antigen, CEA

M.R. Price, P. Tighe & R.W. Baldwin

*Cancer Research Campaign Laboratories, University of Nottingham, Nottingham NG7 2RD, UK.*

The reactivity of a panel of 10 anti-CEA monoclonal antibodies with CEA molecules was analysed using a variety of test procedures. These tests were designed to explore possible relationships between antibody-defined epitopes in order to construct an epitope map of the molecule.

Assay procedures included indirect radioisotopic anti-globulin tests using CEA and the normal tissue component, NCA as target antigens, and double determinant ('sandwich') immunoassays which evaluate both the co-expression of pairs of epitopes defined by different antibodies and their topographical relationships on the CEA molecule. The latter relationships were also analysed by

'cold' antibody inhibition of radiolabelled antibody binding to CEA. Further classification of epitopes was achieved by determining their lability to various chemical, physical (heat) and enzymic treatments.

The epitope map prepared permits the selection of antibodies which react independently with the epitopes on CEA molecules so that these may be employed for the more effective *in vivo* targeting of diagnostic radioisotopes or therapeutic agents (cytotoxic drugs, or plant or bacterial toxins) to the tumour.

### The biological and prognostic significance of DNA ploidy in colorectal cancer

D.J. Jones[1,2], D. Haran[3], M. Moore[1] & P.F. Schofield[2]

[1]*Paterson Institute for Cancer Research and* [2]*Department of Surgery and* [3]*Department of Epidemiology and Social Research, Christie Hospital and Holt Radium Institute, Manchester M20 9BX, UK.*

There is a certain lack of unanimity on the prognostic significance of DNA ploidy in colorectal carcinoma, which is apparent from the different aneuploidy rates (14–67%) in various published studies (Perrez *et al, Br. J. Cancer,* **43,** 526, 1981; Rognum *et al ibid,* **45,** 921, 1982; Woolley *et al, J. Natl Cancer Inst.,* **69,** 15, 1982), and from the interpretation of the biological significance of the phenomenon. 119 consecutive patients presenting from 1981–83 were prospectively studied. DNA ploidy status was determined by flow cytometry according to the method of Hedley *et al.,* (*J. Histochem. Cytochem.,* **31,** 1333, 1983). 39 (33%) were DNA diploid and 80 (67%) DNA aneuploid. 67% of patients with DNA diploid tumours survived 3 years compared with only 35% of those with DNA aneuploid tumours ($P=0.007$). When DNA ploidy status was included in a Cox regression analysis with clinicopathological features, only achievement of 'curative resection', Dukes' stage and age were of independent prognostic significance. DNA index varied randomly between tumours, but was constant within an individual tumour. If an aneuploid population was detected in a lymph node metastasis, it invariably had the same DNA index and prognosis, but there was an inverse relationship between the proportion of aneuploid cells and survival. DNA aneuploidy was not a prerequisite for malignant behaviour and the presence of a detectable aneuploid population did not necessarily result in a highly malignant phenotype. DNA aneuploidy is probably thus an epiphenomenon, reflecting genetic diversity among neoplasms as distinct from malignant status *per se.*

### Flow cytometry and the rat model of azoxymethane-induced colonic neoplasia

G.V.N. Appleton[1], P. Quirke[2], M.F. Dixon[2], C.C. Bird[2] & R.C.N. Williamson[1]

[1]*University Department of Surgery, Bristol Royal Infirmary, Bristol BS2 8HW and* [2]*Department of Pathology, University of Leeds, UK.*

The rodent model of azoxymethane-induced colonic carcinogenesis is helpful in the study of human colorectal cancer. We have used flow cytometry to investigate the presence of DNA aneuploidy in rat intestinal tumours, and to evaluate the rat model as an experimental system. Fifty male Sprague–Dawley rats weighing $185 \pm 9.2$ g were given azoxymethane 15 mg kg$^{-1}$ week$^{-1}$ s.c. for 6 weeks and then underwent 80% small bowel resection ($n=25$) or jejunal transection ($n=25$). Half the animals in each group had calcium lactate 24 g l$^{-1}$ added to the drinking water. Ten further non-operated rats (NOP) received azoxymethane 10 days later than the others. 43 rats survived 26 weeks and yielded 149 colonic and duodenal tumours of which 140 were measurable by flow cytometry. The incidence of DNA aneuploidy was 43% in NOP which was higher than in rats with resection (9%: $P<0.0005$) or transection (24%: $P<0.0005$). There was no significant difference in the prevalence of DNA aneuploidy between adenomas (32%) and carcinomas (17%) or between calcium treated (11%) and non-calcium groups (12%). However metastases were more commonly DNA aneuploid than the primary tumours (62% *vs.* 20%: $P<0.005$). DNA aneuploidy is present in rat intestinal tumours and levels can vary widely with manipulation of the model. Metastases are associated with a high incidence of DNA aneuploidy.

### Electrophoretic studies on protein extracts from normal, diseased and malignant human tissue

R.C.Y. Ng, A.L. Latner & G.A. Turner

*Department of Clinical Biochemistry, The Medical School, The University of Newcastle upon Tyne, Newcastle upon Tyne NE2 4HH, UK.*

It is frequently difficult to diagnose the severity of cancer; there is a need to develop additional tests to help clinicians plan therapy. Analysis of protein composition in tumour biopsies represents one approach to this problem. Healthy, diseased and malignant human tissue biopsies from the breast, cervix and gastrointestinal tract were extracted with Triton X-100. Their protein and glycoprotein composition were investigated using 1D-electrophoresis in SDS-containing gradient polyacrylamide slab gels (Laemmli, *Nature,* **227,** 680, 1970) followed by Coomassie blue (CB) staining and the binding of radio-iodinated wheat germ agglutinin (WGA) (Burridge, *Proc. Natl Acad. Sci., USA,* **73,** 4457, 1976). All results were examined with reference to histopathology. For some tumours, comparisons were made between extracts of the malignant and the healthy or non-malignant tissue of adjacent areas. After CB staining, extracts from the malignant tissues showed more electrophoretic bands when compared with extracts from the control tissues. A particular consistent change was the appearance of extra band(s) at ~50 kd. After staining with radiolabelled WGA, the autoradiographs indicated that certain glycoproteins in the molecular weight range 40–60 kd were much reduced in the malignant extracts. These changes were consistently observed for all the different types of tumour studied, and suggest that this type of analysis may be very useful for diagnosis and prognosis in cancer

### Age-related cell surface proteins of human astrocytoma and mammary carcinoma in culture: Unique age- and tumour grade-related proteins in astrocytomas

G. Hunt & G.V. Sherbet

*Cancer Research Unit, University of Newcastle upon Tyne, Royal Victoria Infirmary, Newcastle upon Tyne NE1 4LP, UK.*

The cell surface protein patterns of primary cultures of 10 human malignant astrocytomas have been investigated and the protein patterns related to the age of the patients. Cultures of 5 fibroadenomas and 5 carcinomas of the breast were also examined. In the carcinomas, the expression of 3

groups of proteins with mol. wt. 63 kd (p63), 48 kd (p48) and 32 kd (p32) was found to be age-related. No age-related proteins were found in the fibroadenoma cultures. There were 4 age-related proteins on the surface of the astrocytoma cultures: 75 kd (p75), p63, p48 and p32. The age-related expression of p75, therefore, appears to be unique to astrocytomas. Of the age-related proteins, tumour grade influenced the levels of p75 only; grade III tumours showed lower levels than grade IV tumours. Although not age-related, the expression of a protein group of mol. wt. 148 kd (p148) was greatly increased in the astrocytomas as compared with normal glial cells. The expression of p148 was strongly influenced by tumour grade, grade III tumours showing lower levels than grade IV tumours. The expression of these age- and tumour grade-related proteins may be of significance in view of the reported patterns of age- and tumour grade-related resistance of malignant glioma to BCNU.

**Two abnormally fucosylated proteins in cancer sera: Markers of tumour burden and of response to therapy**

S. Thompson[1], D. Guthrie[2] & G.A. Turner[1]

[1]*Department of Clinical Biochemistry, The Medical School, Newcastle upon Tyne NE2 4HH and* [2]*Department of Radiotherapy, Newcastle General Hospital, Newcastle upon Tyne NE4 6BE, UK.*

We recently reported a markedly increased level of a diffuse 40–45 Kd band in cancer sera following electrophoresis of fucoprotein extracts from normal and cancer sera. This band being subsequently identified as additional forms of fucosylated haptoglobin $\beta$-chains. In an attempt to determine whether the levels of these haptoglobins correlated with tumour burden, fucosylated haptoglobin was measured in sera from ovarian cancer patients who were receiving chemotherapy. Increased fucosylated haptoglobin correlated well with the extent of tumour burden in all 8 patients who were examined; decreasing as the patients went into remission and increasing as the tumour recurred.

During these latter experiments another fucosylated protein of $\sim$58 Kd (later identified as a fucosylated form of $\alpha$1-antitrypsin) was discovered. Initially, this marker appeared to be related to the recurrence of cancer. Patients who had responded to therapy had low levels of this 58 Kd molecule, even when the initial tumour burden was high. This low level was maintained throughout remission and only became elevated when there was a recurrence of tumour growth. However, further studies on 7 patients who did not respond to therapy revealed elevated levels of this 58 Kd component throughout the period of treatment. The presence of this marker can therefore be used to determine if a patient will not respond to treatment. It would seem possible that we have discovered a new group of serum markers that may be useful for developing better methods to monitor the progress of cancer.

**Use of prostate specific antigen assay in the baseline assessment of patients with advanced prostate cancer**

L.A. Emtage[1], P.W. Lewis[2] & G.R.P. Blackledge[1]

[1]*Cancer Research Campaign Clinical Trials Unit, Queen Elizabeth Hospital, Edgbaston, Birmingham B15 2TH and* [2]*The General Hospital, Birmingham B4 6NH, UK.*

104 patients entering a randomised study comparing ICI Zoladex 3.6 mg with stilboestrol 3 mg day$^{-1}$ were assessed prospectively using an immunoradiometric assay of prostate specific antigen (PSA) (Hybritech-Europe) as well as the usual clinical and biochemical assessments of disease extent. All the patients had either locally advanced or metastatic carcinoma of the prostate. All assessments were carried out prior to therapy. Analysis was made of these entry data.

Using a cutoff point of 10 ng ml$^{-1}$ PSA, the assay gave 92/104 (88.5%) positive values, compared with 68/104 (65.4%) for AP. This difference was highly significant ($\chi^2 = 9.77$, $P < 0.01$). Taken together, PSA and AP gave 95/104 (91.4%) high values. This was not significantly different from PSA alone.

In the asymptomatic group, 7/36 (19.4%) had a normal PSA, compared to 17/36 (47.2%) for AP. Of those with symptoms, 5/68 (7.4%) had a normal PSA, compared to 19/68 (27.9%) for AP. In those with local disease, 8/36 (22.2%) had a normal PSA compared to 20/36 (55.5%) for AP. In those with bone metastases, 4/68 (5.9%) PSA assays were normal, but 16/68 (23.5%) AP were normal. Comparison of PSA against total alkaline phosphatase (ALKP) in those with bone metastases showed a normal PSA in 4/68 (5.9%), but a normal ALKP in 19/68 (27.9%). All of these differences were statistically significant.

The assay is reproducible, and PSA is a better marker of disease extent in advanced prostate cancer prior to treatment than either acid phosphatase measured enzymatically or total alkaline phosphatase in those with bone metastases. Further studies are underway to determine its value in the monitoring of patients with advanced prostate cancer.

### Immunology

**Altered class 1 antigen expression, NK cell sensitivity and metastatic capacity of melanoma cells induced by interferon-$\gamma$**

T.J. McMillan, J. Rao, C.A. Everett & I.R. Hart

*ICRF Laboratories, Lincoln's Inn Fields, London WC2A 3PX, UK.*

We found that IFN-$\gamma$ has a direct effect on malignant tumour cells such that treated cells form an increased number of experimental metastases following i.v. injection into recipient mice.

B16-F1 murine melanoma cells were treated *in vitro* for 48 h with 1000 U ml$^{-1}$ recombinant IFN-$\gamma$. Intravenous injection of $5 \times 10^4$ tumour cells into syngeneic mice resulted in a median of 87 (range 70–99) lung tumour nodules compared with 3 (range 0–15) in mice receiving untreated control cells. A similar increase was seen when cells were injected into athymic nude mice (medians of 3: range 0–8 for control cells and 28; range 19–49 for IFN-$\gamma$ treated cells) but not when the same cells were injected into NK-cell-deficient beige nude mice.

Cells were analysed for class 1 antigen expression by FACS analysis. IFN-$\gamma$ treatment increased H-2 antigen expression with $K^bD^b$ expression rising from 5% of controls to 99% of treated cells.

We have found, like Taniguchi *et al.* (*Int. J. Cancer*, **36**, 503, 1985), an inverse correlation between H-2 class 1 antigen expression and sensitivity to NK cells. Splenic NK cells were less effective *in vitro* against IFN-$\gamma$ treated cells than against untreated controls. (0% cytoxicity *versus* 28% cytotoxicity at 200:1 effector: target cell ratio).

These results are consistent with the possibility that IFN-$\gamma$ pretreatment increases experimental metastatic capacity of melanoma cells by decreasing their NK cell sensitivity. This effect could be associated with the alteration of H-2 class 1 antigen expression since NK cells may serve to recognise cells deficient in these antigens (Karre, K. *et al. Nature*, **319**, 675, 1986).

### Differential expression of HLA antigens on colorectal tumours

A.M. Buckle[1], G. Jacob[2], K. Rogers[2], V. James[3], C.W. Potter[1] & R.C. Rees[1]

[1]*Department of Virology, University of Sheffield Medical School, Beech Hill Road, Sheffield S10 2RX,* [2]*University Department of Surgery, Northern General Hospital, Sheffield and* [3]*Blood Transfusion Centre, Longley Lane, Sheffield, UK.*

We have studied the expression of specific HLA antigens on colorectal carcinomas and colonic epithelium to identify phenotypic differences which may be associated with the acquisition of malignant potential. Serial frozen sections of 30 human colorectal carcinomas and adjacent, normal colonic epithelium were stained with the monoclonal antibodies NFK1 and W6/32 using an indirect immunoperoxidase technique. NFK1 recognised a monomorphic determinant expressed on HLA class II DP, DQ and DR antigens, and W6/32 recognises a framework determinant of class I, A, C and C heavy chain. Individual class I, A and B locus antigens were studied using antibodies against A2 and Bw4 specificities. NFK1 staining revealed areas of class II positive malignant cells in 14/30 tumours and 0/30 of the surrounding mucosa ($P<0.001$ Chi squared). W6/32 staining revealed areas of class I negative epithelium in 7/30 tumours and 0/30 of the surrounding mucosa ($P<0.001$). Loss of individual A and B locus antigens was demonstrated in tumours with W6/32 class I positive epithelium.

The altered expression of HLA antigens was confined to small areas within tumours and confirms the hypothesis of tumour heterogeneity but its exact relationship to the metastatic process is unclear and warrants further investigation.

### Cyclosporin A inhibits the immune response to a mouse monoclonal anti-tumour antibody in rabbits

J.A. Ledermann, R.H.J. Begent, F. Searle T. Adam & K.D. Bagshawe

*Cancer Research Campaign Laboratories, Charing Cross Hospital, London W6 8RF, UK.*

In a study of 15 patients given 2.5 mg of $^{131}$I polyclonal sheep or goat antibody against carcino-embryonic antigen for therapy of advanced colorectal cancer 9/15 (60%) developed human anti-antibodies measured by ELISA. For effective tumour therapy anti-tumour antibodies need to be given on several occasions. The presence of anti-antibodies increases the clearance of the anti-tumour antibody and can lead to severe hypersensitivity reactions if therapy is repeated. We examined methods of inhibiting the immune response to xenogeneic antibodies in rabbits, using the immunosuppressive agent cyclosporin (CyA). The immunogen was 200 $\mu$g of the mouse monoclonal anti-human chorionic gonadotrophin antibody, injected i.v. The antibody was prepared from ascites and immunopurified. Some animals were given antibody which had been 'deaggregated' by ultracentrifugation. The CyA, 20 mg kg$^{-1}$ i.m. was given for 6 days starting day −1 and this regimen was repeated when the animals were re-challenged at 14 days. In those animals given CyA the clearance of the antibody was significantly prolonged and the rabbit anti-mouse antibody response measured by ELISA was suppressed in 8/8 animals given 'deaggregated' antibody and 6/8 given the standard preparation. 'Deaggregated' antibody alone did not inhibit the immune response.

These experiments indicate that CyA may be a suitable agent to inhibit the anti-antibody response in humans to xenogeneic antibodies used for tumour therapy.

### Potentiation of immunotoxin activity *in vitro* and *in vivo*

S.A. Eccles[1] & D.P. McIntosh[2]

[1]*Section of Medicine, Institute of Cancer Research, Sutton, Surrey and* [2]*Chester Beatty Laboratories, Fulham Road, London SW3 6JB, UK.*

Current attempts to develop selective anti-tumour agents include the coupling of plant toxins (or their isolated A chains) to monoclonal antibodies with specificity for tumour cells; however not all antibody-A chain conjugates are effective. One such immunotoxin, comprising a tumour-specific monoclonal antibody (11/160) linked to ricin A chain, was inactive in *in vitro* cytotoxicity assays against $HSN_{tc}$ rat sarcoma target cells. However, addition of ricin B chain as a second-stage reagent resulted in an immunospecific cytotoxicity comparable with that of ricin. *In vivo* studies have shown that i.v. inoculated B chain can also potentiate growth inhibition of conjugate-coated tumour cells in syngeneic rats: Tumour incidence at s.c. sites was reduced to 75% or 30% by 15 $\mu$g or 150 $\mu$g B chain respectively, and lung colonisation was similarly inhibited in a dose-dependent manner. Thus systemically administered ricin B chain is capable of gaining access to antibody-A chain conjugates bound to the surface of sarcoma cells in subcutaneous and pulmonary sites, at concentrations sufficient for a significant therapeutic effect.

### The effect of human recombinant tumour necrosis factor (rHuTNF) and rat gamma interferon (rIFN-$\gamma$) on nitrosomethylurea (NMU) induced rat mammary tumour growth

P. Shah & R.C. Coombes

*Ludwig Institute for Cancer Research, St George's Hospital Medical School, Cranmer Terrace, London SW17 0RE, UK.*

The NMU and DMBA rat mammary tumour models are well established as *in vivo* systems for testing antitumour activity of drugs. We therefore used the NMU-induced primary tumour model to study the effects of rHuTNF and rIFN-$\gamma$ on mammary tumour growth.

An inbred strain of female Ludwig/Wistar/Olac rats with NMU-induced mammary tumours were randomized to either treatment or control groups, each containing 12–18 animals. Treatment groups received either 50 $\mu$g rHuTNF and 30,000 units rIFN-$\gamma$ as a combined dose or 100 $\mu$g TNF alone: control groups received saline. Tumour growth was monitored twice weekly for 4 weeks.

Tumour size in control groups increased almost linearly over this period. Treatment with a combined dose of rIFN-$\gamma$ and rHuTNF reduced tumour growth with 44% regression after 4 weeks. If rHuTNF was administered alone initial tumour regression was noted in the first week ($\sim$25%) after which the tumours appeared to regrow at rates similar to that of control animals. $\gamma$IFN alone had no effect. Evidence from *in vitro* cell culture studies suggests that rIFN-$\gamma$ can up-regulate TNF receptors.

In conclusion we have demonstrated that the combination of rHuTNF and rIFN-$\gamma$ are effective in causing tumour regression after a single dose, whereas each agent alone has little or no effect. This may indicate that the combination may be active in human breast cancer and studies are underway to determine this.

### Suppression of human granulocyte/macrophage colony formation *in vitro* by natural killer cells

A.M. Dickinson, E.A. Jacobs, M.M. Reid & S.J. Proctor

*Department of Haematology, Royal Victoria Infirmary, Newcastle upon Tyne NE1 4LP, UK.*

The effect of natural killer (NK) cell activity in normal human bone marrow on autologous granulocyte/macrophage colony forming activity has been investigated. NK activity was demonstrated against $^{51}$Cr labelled K562 cells in all 7 marrow samples tested (9.1 ± 1.2% $^{51}$Cr release in 4 h) and could be significantly increased by pre-incubation of bone marrow mononuclear cells with αIFN (18.9 ± 2.6). Bone marrow pre-incubated with αIFN produced significantly fewer colonies in bone 7 day (29 + 12/10$^5$ cells) and 14 day (38 + 14/10$^5$ cells) colony assays compared with untreated marrow (88 + 36/10$^5$ cells and 128 + 32/10$^5$ cells respectively).

Removal of active NK cells by Leu llb and complement lysis *in vitro* significantly increased the number of colonies observed in day 7 and day 14 assays ($P<0.01$). These results support the suggestion that NK cells have a role *in vitro* in the inhibition of granulocyte/macrophage colony formation and pose the question as to their involvement in bone marrow graft recovery following *in vivo* bone marrow transplantation.

### Drug resistance and alkylating agents

### Elevation of glutathione S-transferase activity in alkylating agent-resistant cell lines

A.T. McGown & B.W. Fox

*Paterson Institute for Cancer Research, Christie Hospital and Holt Radium Institute, Manchester M20 9BX, UK.*

The glutathione S-transferase (GS-T) enzymes are known to be involved in the deactivation of a wide variety of electrophilic species within the cell. The activity of these enzymes has been measured in cell extracts from parental Yoshida sarcoma cells (YS) together with cell lines which have been made resistant to the alkylating agents cyclophosphamide (YR cyclo), busulphan (YR bus), and methylene dimethane sulphonate (YR 8), by incremental challenge with the relevant drug *in vitro*. Glutathione S-transferase activities were determined spectrophotometrically using 1-chloro-2, 4-dinitrobenzene (CDNB) and glutathione as cosubstrates. The resistant cell lines all show elevated GS-T activities compared with the parental cell line. The levels of elevation are 4-fold for the YR bus and YR 8 cell lines, and 6-fold for the YR cyclo cell line. All three alkylating agents can inhibit GS-T activity against CDNB. It is proposed that a part of the resistance mechanism operating in these cell lines is due to an increased deactivation of alkylating species by the elevated levels of GS-T enzymes. This may also account for the cross resistance observed between these cell lines and alkylating agents, as well as the decreased damage to cellular DNA, as measured by alkaline elution and inhibition of DNA synthesis, observed in the resistant cell lines.

### The relative effectiveness of alternative platinum complex drugs in the experimental chemotherapy of human tumour spheroids

J. Russell, J. Adam, T.E. Wheldon & S.B. Kaye

*Glasgow Institute of Radiotherapeutics and Oncology, Belvidere Hospital, Glasgow G31 4PG and Department of Medical Oncology, University of Glasgow, UK.*

*Cis*-platin is a potent anticancer drug whose use is restricted by toxicity. Less toxic analogues (JM8, JM9) have recently been introduced but their anti-tumour potencies are not yet well established. We have compared the cytotoxic activities of *cis*-platin, JM8 and JM9 by assessing growth delay in multicellular spheroids derived from human tumours. A neuroblastoma line (NB1-G) and one derived from non-small-cell lung cancer (L-DAN) were used. L-DAN was found to be about ten times more resistant (in terms of concentration) to both JM8 and JM9 than *cis*-platin. NB1-G was 10× more resistant to JM9 but 40× more resistant to JM8 than to *cis*-platin. The clinical effectiveness of JM8 and JM9 may depend on whether they can be safely used at such enhanced concentrations.

### Immunochemical detection of *cis*-platin-DNA adducts in human testicular and bladder tumour cell lines

P. Bedford[1,2], A.M. Fichtinger-Schepman[3], J.R.W. Masters[2] & B.T. Hill[1,2]

[1]*Imperial Cancer Research Fund Laboratories, Lincoln's Inn Fields, London WC2A 3PX;* [2]*Institute of Urology, Endell Street, London WC2H 9AE, UK and* [3]*Medical Biological Laboratory, TNO, 2280 AA Rijswijk, The Netherlands.*

Polyclonal antisera raised to synthetic platinated nucleotides coupled to bovine serum albumin were used to quantitate four Pt-DNA-adducts in DNA extracted from two human teratoma cell lines (SUSA; 833K), a subline derived by fractionated X-irradiation (SUSA-DXR$_{10}$) and a bladder carcinoma cell line (RT112), each exposed for 1 h to 20 μg ml$^{-1}$ *cis*-platin. Digested DNA was separated on an anion exchange column and adducts measured by competitive ELISA. The following adducts were detected: *cis*-Pt(NH$_3$)$_2$d(pGpG) [Pt-GG], *cis*-Pt(NH$_3$)$_2$d(pApG), monofunctionally platinated DNA: Pt(NH$_3$)$_3$dGMP and an adduct derived from DNA interstrand crosslinks and intrastrand crosslinks between guanines separated by intervening bases: *cis*-Pt(NH$_3$)$_2$d(GMP)$_2$. The major adduct was Pt-GG (70%–80%) and whilst the overall distribution of adducts was similar in all lines, the total amount of platination varied considerably, for example:

| *Cell line* | *Pt-GG (nmol g$^{-1}$ DNA)* |
|---|---|
| SUSA | 294 |
| SUSA-DXR$_{10}$ | 301 |
| 833 K | 78 |
| RT112 | 252 |

The possibility exists that differences in induction and repair of Pt-DNA adducts might be related to the differential sensitivities of these lines to *cis*-platin.

### Determination of urinary alkylated nucleic acid bases by immunoassay

D.E.G. Shuker

*International Agency for Research on Cancer, 150 cours Albert-Thomas, 69372 Lyon Cedex 08, France.*

Many carcinogens react with DNA to give alkylated purines

(e.g. 7-alkylguanine, 3-alkyladenine) which are subsequently released by glycosylases and excreted in urine. The simple and reliable determination of such alkylated bases in human urine could be used in 'molecular epidemiology' studies to evaluate carcinogen exposure. However, the determination of low molecular weight adducts (e.g. methyl, ethyl, etc) of urinary purines by procedures such as immunoassay have hitherto been unsuccessful.

A new approach has been developed which enables antibodies to be raised which recognise such adducts.

A novel analogue of 3-methyladenine (3-MeA) containing a carboxyl group was covalently conjugated to methylated BSA (7 moles hapten per mole of carrier). After following a standard immunisation schedule in rabbits, antisera were obtained which recognised, at high dilution, 3-MeA conjugated to keyhole limpet haemocyanin (KLH). Preliminary experiments indicate that the free base, 3-MeA, is also recognised by the antisera. A quantitative assay for urinary 3-MeA is currently being developed using a competitive ELISA technique.

A similar protocol is also being used for other alkylated adenines and guanines.

### *In vivo* sensitization of nitrosourea resistant Lewis lung tumour cells to MeCCNU, by pretreatment with MNU

T.C. Stephens, J. Eady, J.H. Peacock, G. Harris & P.D. Lawley

*Institute of Cancer Research, Surrey, UK; ICI Pharmaceuticals Division, Cheshire, UK and Kennedy Institute of Rheumatology, London, UK.*

Mice bearing wild-type Lewis lung tumours and a MeCCNU resistant subline, were treated sequentially *in vivo* with a mono- and a bi-functional nitrosourea (MNU and MeCCNU respectively) to test the effect of combined treatment with these agents. MNU (10 or 30 mg $kg^{-1}$ was administered 2 h before MeCCNU (5 to 15 mg $kg^{-1}$) and the response of tumours was measured 24 h later using an excision clonogenic assay. With the wild-type tumour, both agents were active, and the combined drug effect was simply additive. However, in the nitrosourea resistant tumour line, MNU had very little effect alone, yet cell killing due to the combination was much greater than additive. Biochemical studies with radiolabelled MNU have suggested that nitrosourea resistance was due to over expression of the repair psuedo-enzyme, $0^6$-methylguanine-DNA methyltransferase. This may account for the synergistic combined response in the resistant tumour line as follows: Pretreatment with MNU should lead to depletion of repair pseudo-enzyme in nitrosourea resistant cells, as the monofunctional lesions are repaired. Subsequent treatment with a bi-functional agent should then be much more effective. If the MeCCNU treatment was delayed until 24 h after MNU, when new repair pseudo-enzyme had been synthesized, synergy was lost. In the wild-type tumour, which has very little repair capacity, lesions produced by each agent should be simply additive.

### A comparison of DNA-protein crosslinking in chromatin following methylene dimethane sulphonate and formaldehyde treatment

P.M. O'Connor & B.W. Fox

*Paterson Institute for Cancer Research, Christie Hospital and Holt Radium Institute, Manchester M20 9BX, UK.*

Methylene dimethane sulphonate (MDMS) possesses excellent antitumour activity against the rodent Yoshida lymphosarcoma and is currently undergoing Phase II clinical trials in Europe. MDMS is, however, rapidly hydrolysed with a half life of 22 min at 37°C to release formaldehyde (HCHO), and methane sulphonic acid (MSA). Both MDMS and HCHO produce DNA-protein cross-linking, but only MDMS causes DNA-DNA interstrand crosslinks. MDMS induced cytotoxicity in Yoshida (YS) cells could be correlated with DNA-DNA inter-strand crosslinks and not with DNA-protein crosslinks as assayed in the Alkaline Elution procedure.

However, SDS-polyacrylamide gel electrophoresis of DNA-crosslinked proteins following MDMS and HCHO teatment revealed some interesting differences in the proteins crosslinked to DNA. Only, MDMS cross-linked proteins of 29 and 48 Kd to DNA. Whilst, HCHO crosslinked a 26 Kd protein to DNA that was not observed following MDMS treatment. HCHO was shown to be responsible for all DNA-protein crosslinking seen after MDMS treatment. The differences in proteins crosslinked to DNA by MDMS and HCHO could be attributed to the simultaneous release of MSA in the molecular environment of the nucleophilic site.

The 26 Kd 'HCHO related' protein has been identified as a H4-H2b histone protein dimer and the 29 Kd 'MDMS related' protein as a H2a-H2b histone dimer. The selective formation of the H2a-H2b histone dimer following MDMS treatment is believed to result from a pH dependent alteration in nucleosome structure induced by MSA. The 48 Kd 'MDMS related' protein is still under study.

### Induction of hypoxia in tumours in order to exploit the cytotoxicity of bioreductive drugs

I.J. Stratford & G.E. Adams

*MRC Radiobiology Unit, Chilton, Didcot, Oxon OX11 0RD, UK.*

RSU 1069 is a compound which shows 100 × greater toxicity towards hypoxic relative to aerobic mammalian cells *in vitro*. In air the compound acts as a typical monofunctional alkylating agent whereas in hypoxia RSU 1069 is reduced to yield a highly active bifunctional cytotoxic species. In the past, treating tumour bearing animals with bioreductive compounds, such as RSU 1069, as single agents, has shown little benefit. This is due to the fact that solid tumours are a mixed population of hypoxic and aerobic cells and the latter will be resistant to bioreductive agent therapy. In this study, we have taken the approach of *selectively* rendering tumours hypoxic allowing the potential toxicity of RSU 1069 to be fully expressed *in vivo*. We have used two methods for selectively reducing tumour oxygenation. Firstly, by using the agent BW12C, a compound that causes an increase in the $0_2$ affinity of haemoglobin and secondly, by the use of anti-hypertensive agent, hydralazine. Both these methods can result in tumours being rendered close to 100% hypoxic for several hours. Treatment of mice bearing the Lewis Lung carcinoma with 5 mg $kg^{-1}$ hydralazine i.v. 15 min following administration of 80 mg $kg^{-1}$ RSU 1069 i.p. results in a reduction in tumour cell survival to $5 \times 10^{-3}$, whereas RSU 1069 alone results in only 50% cell kill. Hydralazine itself has no cytotoxic effect at this dose. A similarly substantial degree of cell kill is also to be found in the KHT sarcoma using this drug combination. Further, hydralazine does not cause any significant increase in the whole body toxicity of RSU 1069 indicating that this approach may be therapeutically beneficial.

**Hyperthermia-stimulated nitroreductive bioactivation of the 2-nitromidazole benznidazole *in vitro* and *in vivo***

M.I. Walton, N.M. Bleehen & P. Workman

*MRC Unit and University Department of Clinical Oncology and Radiotherapeutics, Hills Road, Cambridge CB2 2QH, UK.*

Hyperthermia enhances the cytotoxicity of nitroheterocyclic drugs *in vitro* and *in vivo*, possibly through increased reductive metabolism of the nitro group. Using an HPLC assay for the 2-nitroimidazole benznidazole (BENZO) and its corresponding amine metabolite, we have studied the effects of temperature (33–44°C) on BENZO amine formation kinetics *in vitro*. Reactions were carried out using mouse liver microsomes and whole KHT tumour homogenates under $N_2$ in the presence of NADPH and NADH. Hyperthermia markedly increased BENZO amine formation rates in both microsomes and tumour homogenates up to 41°C e.g. tumour reduction rates were increased by 35% from 42.5 to 57.4 pmol min$^{-1}$ mg$^{-1}$ protein at 41 compared to 37°C. At 44°C microsomal reduction rates were increased by 0–54% depending on substrate concentration, whereas tumour rates were substantially increased (26–79%) at both 1 and 0.1 mM BENZO. We have also studied the effects of local hyperthermia (LH) on BENZO reduction to its amine metabolite in C3H/He mice with KHT i.m. leg tumours. LH (waterbath + radiofrequency heating; 43.5°C x 30 min) was given to conscious mice 2.5 h after 2.5 mmol kg$^{-1}$ BENZO i.p. LH greatly increased tumour amine levels compared to controls, e.g. by 81% from $4.75 \pm 1.6$ to $8.61 \pm 0.91$ $\mu$g g$^{-1}$ (mean $\pm$ 2s.e., $n=6$; $P<0.01$) immediately after heating. Parent BENZO concentrations were correspondingly decreased, e.g. by 58% from $137 \pm 37.4$ to $57.2 \pm 5.84$ $\mu$g g$^{-1}$ ($P<0.01$). Plasma and liver BENZO and BENZO amine concentrations were similar in heated and control mice. These results clearly demonstrate that hyperthermia can enhance the reductive bioactivation of BENZO *in vitro* and *in vivo*. In view of the targetable nature of LH, these results have important implications for its use in combination with bioreductively activated antitumour agents.

### Posters

**NCL-5D3: An anti-cytokeratin monoclonal antibody raised against proteins released by breast cancer cells in tissue culture and recognising simple epithelia**

B. Angus, J. Purvis, D. Stock, S. Kiberu, E. Routledge, B. Westley, F. Carpenter & C.H.W. Horne

*Department of Pathology, University of Newcastle upon Tyne, UK.*

Proteins secreted or shed by breast cancer cells are of interest as potential tumour markers. The aim of our study was to generate monoclonal antibodies against proteins secreted or shed by MCF7 breast cancer cells in tissue culture. A standard hybridoma protocol using BALB-C mice and NS1 cells was followed. The immunogen used was an 'all protein' preparation derived from serum free supernatants of confluent MCF7 cell cultures. Antibodies were screened by immunocytochemistry using an indirect immunoperoxidase technique on breast carcinoma tissue sections. The fusion described here yielded 12 antibodies showing reactivity, and two of these showed high specificity for tumour cells with low background staining. One has not yet been fully evaluated. The other has been designated NCL-5D3. Comparison with the anticytokeratin antibody PKK1 shows a very similar westen blot pattern, with reactivity against several bands between 38 and 46 Kd. Screening against a wide variety of normal and neoplastic tissues using an indirect immunoperoxidase technique on formalin fixed paraffin embedded tissues has demonstrated reactivity with all simple epithelia tested and with almost all tumours derived from such epithelia. For example, 60 out of 60 breast carcinomas showed strong reactivity. No reactivity was observed against non-epithelial tissues or against stratified squamous epithelium, and only a small proportion (5 of 17) of squamous carcinomas showed weak reactivity. As the antibody works well with fixed, embedded tissue and shows more intense reactivity than some currently available monoclonal antibodies recognising simple epithelia, NCL-5D3 should prove of value in diagnostic histopathology.

**Characterisation of ZR-75-1 human breast cancer cells grown in phenol red free tissue culture medium**

H.W. van den Berg & M. Lynch

*Department of Therapeutics and Pharmacology, The Queen's University of Belfast, Northern Ireland, UK.*

It has been shown recently that the pH indicator phenol red, (PR), has significant oestrogenic activity at concentrations used in tissue culture medium, (Berthois *et al*, *Proc. Natl Acad, Sci., USA*, **83**, 2496, 1986). This finding has important implications for studies designed to elucidate the mechanisms of action of oestrogens and anti-oestrogens using human breast cancer cell lines in long-term culture.

In culture medium containing PR and 5% dextran coated charcoal stripped foetal calf serum (FCSdcc), cell population doubling time, (DT), of ZR-75-1 human breast cancer cells was 2–2.9 days for cells initially plated at $1\text{–}5 \times 10^4$ per microwell plate. Cells expressed both ER, ($147 \pm 19$ fmol mg$^{-1}$ protein) and PGR, ($80 \pm 24$ fmol mg$^{-1}$ protein) as assessed using a whole cell binding assay. Cells transferred to PR free medium containing 5% FCSdcc showed an immediate slowing of growth rate at an initial seeding density of $5 \times 10^4$ cells per well, (DT 4.1–5 days). Continuous exposure to oestradiol, ($10^{-9}$ M), shortened DT to 3 days. This effect was most consistent when the serum concentration was raised to 20%. PR deprived cells retained sensitivity to oestrogen reversible growth inhibition by 4-hydroxy tamoxifen ($10^{-9} - 10^{-7}$ M). Cells plated at densities $<4 \times 10^4$/well failed to proliferate in the absence of phenol red.

The DT of cells deprived of PR for 4 months has extended to 8.4–10 days and remains at 5.6–6 days in the presence of oestradiol. ER content, ($157 \pm 15$ fmol mg$^{-1}$), is not significantly different from that of routinely cultured cells whilst PGR are undetectable but inducible by oestrogen treatment.

Our data show that ZR-75-1 cells are capable of proliferation in the absence of oestrogenic stimulus but proliferation is density dependent. Our observations would be consistent with the proposal that cells are capable of low level autonomous secretion of oestrogen inducible growth factor(s).

**Quantitation of microvilli density on MCF-7 cell surface**

J. Nelson, R. Clarke, N.V. McFerran & R.F. Murphy

*Department of Biochemistry, The Queen's University of Belfast, Belfast BT9 7BL, UK.*

Oestrogen is known to stimulate the production of microvilli (MV) in target cells. Phenol red is ubiquitously used in tissue culture media, but it has recently been shown to be a weak oestrogen (Berthois *et al.*, *Proc. Natl Acad, Sci, USA*, **83**, 2496, 1986). We have examined the effect of phenol red withdrawal on hormone free MCF-7 cells which have been maintained in steroid-free medium (containing phenol red) for 2 years. Cell surfaces of fixed cells were examined by scanning electron microscopy and MV density per 482 $\mu m^2$ was measured by computerized image analysis (McFerran & Quigley, *Biochem. Soc. Trans.*, **12**, 1000, 1984). It was found that MV density decreased following phenol red withdrawal and reached a minimum after 2 weeks. Restimulation of phenol red- and steroid-withdrawn cells resulted in increases in MV density as shown in the Table.

MV density (percentage of control)

| | *Phenol red free* | | *Restimulated* | |
|---|---|---|---|---|
| *HFMCF-7* | *1 week* | *2 week* | *(E2)*[a] | *(PR)*[a] |
| 100 (+14) | 61 (+21) | 14 (+10) | 110 (+28) | 89 (+35) |

[a]E2: $10^{-1}$ M oestradiol. PR: 48 $\mu$M phenol red.

Cells were steroid and phenol red free for 2 weeks prior to restimulation for 3 days.

### Morphological and functional characteristics of mouse mammary carcinoma cells separated on nycodenz columns

T.C. Ford, T. Lai & M.O. Symes

*University Department of Surgery, Bristol Royal Infirmary, Bristol BS2 8HW, UK.*

Tissue from 4 mouse mammary carcinomas was enzymatically disaggregated and cells from the resulting cell suspension were fractionated on a discontinuous density gradient column (5–20%) of Nycodenz (Nycomed A.S. Oslo). The cell fractions separating at the 10–15 and 15–20% interfaces (density 1.082 and 1.110 g ml$^{-1}$ respectively) contained a mean of 83.2 ± 10.8 (s.d.) and 79.9 ± 17.4 tumour cells. Compared with the original cell suspension these cell bands contained less cell aggregates and cell debris. Also the cells in the bands showed an equivalent ability to grow in tissue culture and to form pulmonary tumours on i.v. injection into isogenic mice, when compared with the tumour cells in the original suspension. The relatively pure preparations of carcinoma cells thus separated may be of value in limiting the unwanted effect of normal cell contamination when testing the neoplastic cells *in vitro* for sensitivity to drugs or hormones.

### The effects of glucagon on ZR-75-1 human breast cancer cells

M. Cremin, R. Clarke, J. Nelson & R.F. Murphy

*Department of Biochemistry, The Queen's University of Belfast, Medical Biology Centre, 97 Lisburn Road, Belfast BT9 7BL, Northern Ireland, UK.*

We have examined the effects of glucagon on the oestrogen responsive ZR-75-1 human breast cancer cell-line. While $10^{-10}$ M to $10^{-6}$ M glucagon fails to influence the rate of DNA synthesis, the rate of [$^3$H]-leucine incorporation into gross protein is stimulated to greater than 150% of that determined in untreated cell populations. Maximal stimulation is observed following exposure to concentrations of glucagon from $10^{-9}$ M to $10^{-7}$ M.

Specific binding of [$^{125}$I]-glucagon was demonstrated using a competitive binding assay. Bmax and Kd of glucagon binding are 5.18 ± 0.68 fmol $10^{-6}$ cells and 1.02 nM respectively. This Kd is comparable with that found for the high affinity receptors in canine hepatocytes (Bonnevie-Nielsen & Tager, *J. Biol. Chem.*, **258**, 11313, 1983) and reflects the concentration range where maximal biological response is observed.

### The effect of 17β oestradiol (E2) and tamoxifen (TAM) on the membrane fluidity of both oestrogen responsive and unresponsive human breast cancer cells

R. Clarke[1], H.W. van den Berg[2], J. Nelson[1] & R.F. Murphy[1]

*Departments of [1]Biochemistry and [2]Therapeutics, The Queen's University of Belfast, Medical Biology Centre, 97 Lisburn Road, Belfast BT9 7BL, UK.*

Whilst the majority of breast tumours which respond to endocrine manipulation contain oestrogen receptors (ER) some tumours which do not have ER respond to these therapies. We have examined the ability of pharmacological and suprapharmacological concentrations of both E2 and TAM to modulate the membrane fluidity of MCF-7 (E2 responsive) and MDA-MB-436 (E2 unresponsive) cells as determined by the steady-state polarisation of fluorescence of the probe 1,6 diphenylhexatriene. The ability of E2 and TAM to influence cell proliferation was also determined. E2 produces an equivalent increase in membrane fluidity in both cell lines, oestrogenic effects being observed only in MCF-7 cells. In both MCF-7 and MDA-MB-436 cells $10^{-5}$ M E2 is equitoxic and produces similar perturbations in membrane fluidity. $10^{-6}$ M TAM decreases the membrane fluidity in both cell lines but inhibition of proliferation reversible by E2 is observed only in MCF-7 cells. $10^{-5}$ M TAM is less cytotoxic towards MDA-MB-436 cells than MCF-7 cells and produces less perturbation of membrane fluidity.

The inhibitory effects of high doses of E2 and TAM are unlikely to be mediated through the ER and may be the result of altered membrane function. We have previously demonstrated that $10^{-6}$ M E2 reduces the steady-state levels of methotrexate in both MCF-7 (Clarke *et al*, *Br. J. Cancer*, **51**, 365, 1985) and MDA-MB-436 cells (Clarke *et al*, *Eur. J. Cancer Clin. Oncol.*, **17**, 1275, 1983). These results may reflect a physical restriction of the folate membrane-transport system caused by the E2-induced reductions in membrane fluidity.

### Energy expenditure and protein synthesis rates in an animal model of cancer cachexia

J.A. Plumb[1], K.C.H. Fearon[2], K. Carter[2], H.J.G. Burns[2] & K.C. Calman[3]

*Departments of [1]Medical Oncology, [2]Surgery and [3]Postgraduate Medical Education, University of Glasgow, Glasgow, UK.*

Although anorexia is often associated with cancer cachexia the reduced food intake alone is not sufficient to produce a severe weight loss. Increased rates of both protein synthesis and energy expenditure have been proposed to account for the weight loss. We have investigated both possibilities in an animal model of cancer cachexia. A chemically induced adenocarcinoma of the mouse colon (MAC-16) grown subcutaneously in NMRI mice produces a 20% loss of body wt after 4 weeks. However, the food intake of the mice remains constant until the fourth week after tumour implantation. At this time the tumour represents only 6% of the mouse body wt. Energy expenditure was measured over a 24 h period by indirect calorimetry both before and 1, 2, 3 and 4 weeks after tumour implantation. Although weight loss commenced one week after tumour implantation there was no change in either oxygen consumption or carbon dioxide production, expressed per mouse, even after 4 weeks of tumour growth. However, since the mice lost weight during tumour growth the rate of energy expenditure increased when expressed $g^{-1}$ body wt. Protein synthesis rates were measured *in vivo* by the flooding phenylalanine method. Although the protein content of the gastrocnemious muscle was decreased by 35% four weeks after tumour implantation there was no change in the rate of protein synthesis in skeletal muscle. Thus in this model weight loss cannot be explained by a decreased food intake, an increased energy expenditure or an increase in the rate of protein synthesis.

### Cachectic factor(s) produced by the MAC 16 adenocarcinoma

R.A. Brennan, S.A. Beck & M.J. Tisdale

*CRC Experimental Chemotherapy Group, Department of Pharmaceutical Sciences, Aston University, Birmingham B4 7ET, UK.*

The MAC 16 is a transplantable colon adenocarcinoma which produces extensive weight loss in tumour-bearing animals without a reduction in food or water intake. In males a 0.6 g tumour will produce 33% loss of body weight within 35 days of tumour transplantation. Body composition analysis shows a progressive decrease in adipose tissue and muscle mass without a change in body water. While plasma glutamine levels are elevated 25% in tumour-bearing animals the plasma concentrations of most other amino acids including glycine are reduced by 30–40% and thus the situation differs from chronic malnutrition. Cell-free extracts of the MAC 16 tumour cause a release of free fatty acids (FFA) from mouse fat pads while extracts from two colon carcinomas, which do not produce cachexia in recipient animals, MAC 13 and MAC15A, have no effect on FFA release. The FFA releasing activity of the MAC 16 tumour is dramatically reduced after acid or heat treatment. Cell-free extracts of the MAC 16 tumour also cause an enhanced release of amino acids from mouse diaphragm, while extracts from MAC 15A do not. These results suggest that the cachexia produced by the MAC 16 tumour may be due to the presence of a tumour-associated catabolic factor.

### Thymidylate synthase (TS) activity in L1210 cells following *in vitro* or *in vivo* exposure to quinazoline antifolates

G.A. Taylor, A.L. Jackman, K. Balmanno & A.H. Calvert

*Drug Development Section, Institute of Cancer Research, Sutton, Surrey, UK.*

$N^{10}$-propargyl-5,8-dideazafolate (CB3717) lacks antitumour activity against a range of rodent tumours despite demonstrable clinical efficacy. This apparent anomaly may be due, at least in part, to rodents having higher circulating levels of thymidine (THd) which may circumvent the cytotoxic locus of CB3717, i.e. inhibition of TS. In the absence of a suitable tumour model methods have been developed to measure the *in situ* activity of enzymes of deoxypyrimidine metabolism in tumour cells following *in vitro* or *in vivo* exposure to CB3717 and its congeners. This involves monitoring the *in vitro* distribution of radiolabelled precursors under conditions where the intracellular deoxypyrimidine nucleotide pools are in a state of dynamic equilibrium. Studies of L1210 cells incubated with 5[$^3$H] deoxyuridine revealed that the rapid inhibition of TS following short exposures (<4 h) to either CB3717 or its 2-desamino analogue (desamino-CB3717) recovers rapidly following resuspension in fresh medium. However, with longer exposure times prolonged TS inhibition was observed, presumably due to the formation of non-effluxable products (e.g. polyglutamates). After 15 h exposure (to a dose $10\times IC_{50}$, in the presence of 10 $\mu$M Thd) the release of tritiated water (via TS) from these cells was inhibited by >90% in the absence of extracellular compound. Following resuspension in fresh medium containing Thd, this inhibition was maintained (>75% after 8 h). Similarly we have demonstrated the inhibition of tritiated water release in L1210 cells following *in vivo* treatment with both analogues (100 mg kg$^{-1}$, i.v.). The inhibition observed with desamino-CB3717 was much greater and more prolonged than that seen with CB3717. It is therefore now possible to compare the effects of analogues upon the target enzyme while retaining effects due to route of administration, pharmacokinetics etc.

### Polyglutamation of the thymidylate synthase (TS) inhibitor $N^{10}$-propargyl-5,8-dideazafolic acid (CB3717) in L1210 cells *in vitro*

E. Sikora, A.L. Jackman, D.R. Newell & A.H. Calvert

*Drug Development Section, Institute of Cancer Research, Sutton, UK.*

Polyglutamation is a common intracellular fate for both natural folates and synthetic antifolates. We have investigated the formation and retention of CB3717 polyglutamates in L1210 cells. Cells were exposed to 50 $\mu$M $^3$H-CB3717 (10 x IC50) in the presence of thymidine (10 $\mu$M) to prevent cell death. CB3717 polyglutamates were identified by co-chromatography of $^3$H-metabolites on HPLC (10 x 0.46 cm Polygosil 5 $\mu$m C18 column, 5–16% $CH_3CN$ in 0.1 M NaAc pH 5, 15 min) with standards and by their ability to inhibit TS. The results showed extensive CB3717 polyglutmate formation.

| *Incubation time (h)* | *Cellular $^3$H ($\mu$M)* | *% Cellular $^3$H as* CB3717(Glu1) | Glu3 | Glu4 | Glu5 |
|---|---|---|---|---|---|
| 6 | 4.5±1.5 | 92,71 | 0,2 | 5,17 | 2,4 |
| 12 | 6.8±3.6 | 57±3 | 3±1 | 23±4 | 13±1 |
| 24 | 5.9±3.4 | 51±5 | 1±1 | 20±1 | 21±3 |

After 24 h incubation followed by resuspension in drug free medium for 6 and 24 h, parent CB3717 comprised only 5% and 2% of the cellular 3H, respectively. In contrast, CB3717 glu4 and glu5 were retained with levels declining solely due to dilution during cell division. Measurement of whole cell TS activity using [6-$^3$H]deoxyuridine incorporation into DNA indicated that complete suppression of activity (<10% control) was maintained throughout the 24 h resuspension period. Thus despite poor cellular accumulation CB3717 undergoes polyglutamation (~50% at 50 $\mu$M) in L1210 cells *in vitro*. The products are retained following removal of extracellular drug and by virtue of their potent activity give rise to maintained TS inhibition.

**Some biological properties of 2-desamino-N$^{10}$-substituted 5,8-dideazafolates**

B.M. O'Connor, A.L. Jackman, J.A.M. Bishop, T.R. Jones, T.J. Thornton & A.H. Calvert

*Institute of Cancer Research, Sutton, Surrey, UK.*

N$^{10}$-substituted 5,8-dideazafolates are dual inhibitors of thymidylate synthase (TS) and dihydrofolate reductase (DHFR) although inhibition of TS appears to be the usual cytotoxic event. The N$^{10}$-propargyl compound (CB3717) acts purely as a TS inhibitor and has anti-tumour activity in man. We have now synthesised 2-desamino-5,8-dideazafolates with aliphatic N$^{10}$-substituents which are up to 10-fold more cytotoxic despite being poorer inhibitors of TS and DHFR. In both series the N$^{10}$-propargyl compound was the most potent against TS ($I_{50}$ desamino-CB3717 = 16 $\mu$M; $I_{50}$ CB3717 = 0.02 $\mu$M) i.e., propargyl > ethyl > methyl > allyl > hydrogen. Against DHFR hydrogen > methyl > ethyl > allyl > propargyl (desamino-CB3717 Ki = 2.25 $\mu$M; CB3717 = 0.075 $\mu$M). Hence propargyl has the lowest TS/DHFR ratio, an important property for a compound to be rate-limiting on TS. The activities against 3 cell lines are shown below. The figures in brackets represent the values obtained with the equivalent 2-amino compounds.

| $N^{10}$ | *WI-L2* ($IC_{50}$ $\mu$M) | *L1210* ($IC_{50}$ $\mu$M) | *R7A* $IC_{50}$ ($\mu$M) (*DHFR over-producing*) | *R7A/L1210* (*ratio*) |
|---|---|---|---|---|
| H | 0.26 (0.48) | 0.43 (2.68) | 426 (300) | 991 (111) |
| $CH_3$ | 0.72 (1.60) | 0.91 (4.5) | 335 (230) | 368 (51) |
| $CH_2CH_3$ | 2.78 (1.32) | 2.65 (9.0) | 390 (240) | 144 (27) |
| $CH_2CH=CH_2$ | 1.87 | 2.48 (6.0) | – | – |
| $CH_2C\equiv CH$ | 0.55 (2.45) | 0.4 (3.4) | 7 (38) | 19 (11) |

In both series the R7A/L1210 ratio decreased as the N$^{10}$-chain length increased (paralleling the decrease in the Ki TS/Ki DHFR ratio). The greater degree of cross-resistance towards the desamino series is not readily explained.

**2-desamino-10-propargyl-5,8-dideazafolic acid (desamino-CB3717) a thymidylate synthase (TS) inhibitor devoid of renal and hepatic toxicities in mice**

A.L. Jackman[1], D.R. Newell[1], G.A. Taylor[1], B.M. O'Connor[1], L. Hughes[2] & A.H. Calvert[1]

[1]*Institute of Cancer Research, Sutton, Surrey and* [2]*ICI Pharmaceuticals, Macclesfield, Cheshire, UK.*

N$^{10}$-Propargyl-5,8-dideazafolate (CB3717) has demonstrated the clinical potential of inhibiting TS in that it has activity against breast, ovary and liver cancer. However the dose-limiting renal toxicity together with reversible hepatic toxicity has led us to seek less toxic analogues. We report here the interesting biological properties of desamino-CB3717, an inhibitor of L1210 TS (Ki = 26.4 ± 3.53 nM). Despite being 7-fold less potent than CB3717 as an inhibitor of TS it is 10-fold more potent than CB 3717 against cultured mouse L1210 cells ($IC_{50}$ = 0.35 $\mu$M). Other properties include (i) weak inhibition of dihydrofolate reductase (Ki = 2.25 ± 0.23 $\mu$M), (ii) thymidine reversal of cytotoxicity (3.5 $\mu$M) in L1210 cells (iii) inactivity at 200 $\mu$M against a TS over-producing cell line (L1210:C15). Following a single i.v. injection of 100 mg kg$^{-1}$ desamino-CB3717 to L1210 i.p. tumour-bearing mice, tumour TS activity was inhibited by > 60% for 6 h with recovery by 24 h. The effect was more pronounced and for a longer duration than with CB3717 at the same dose despite a very much more rapid plasma clearance (1752 ml h$^{-1}$ kg$^{-1}$; CB3717 = 300 ml h$^{-1}$ kg$^{-1}$). In acute toxicity studies (0–24 h) desamino-CB3717 was considerably less toxic than CB3717 in mice. A 500 mg kg$^{-1}$ i.v. dose of desamino-CB3717 (plasma AUC 620 $\mu$M h) caused no renal or hepatic toxicities. This contrasted with a 100 mg kg dose of CB3717 (plasma AUC 680 $\mu$M h) which caused toxicity to both these organs as evidenced by histopathological damage to the kidneys and elevations in plasma urea (100%), and alanine transaminase (4000%). The reduced toxicity of desamino-CB3717 is possibly a result of its greater solubility (> 1000-fold more soluble than CB3717 at physiological pH).

**Potentiation of CB3717 toxicity by dipyridamole in A549 cells**

N.J. Curtin & A.L. Harris

*Cancer Research Unit, University of Newcastle upon Tyne, Royal Victoria Infirmary, Newcastle upon Tyne NE1 4LP, UK.*

CB3717 is an antifolate inhibitor of thymidylate synthase which does not affect other pathways. The only means of circumventing such inhibition is via salvage of exogenous thymidine. Dipyridamole is a nucleoside transport inhibitor that has been shown to enhance the toxicity of some drugs by the prevention of salvage and others by the prevention of the efflux of toxic metabolites.

Using A549 lung carcinoma cells, we have shown that 1 $\mu$M dipyridamole reduces the uptake of [$^3$H]thymidine by over 95% in both control and CB3717 treated cells. At this concentration, dipyridamole does not affect cell growth but significantly reduces the $ID_{50}$ of CB3717 from 2.376 ± 0.533 $\mu$M to 0.979 ± 0.279 $\mu$M ($P$ < 0.001). Elimination of exogenous salvagable thymidine by the use of dialysed serum also caused a significant reduction in the $ID_{50}$ of CB3717 to 1.497 ± 0.956 $\mu$M ($P$ < 0.05). The greater potentiation of CB3717 toxicity by the dipyridamole suggests a second mechanism for dipyridamole action. If the toxicity of thymidylate synthase inhibition is due to misincorporation of dUTP into DNA followed by its excision, leading to strand breaks, then the intracellular level of dUTP will be important. Our experiments show that 1 $\mu$M dipyridamole inhibits the efflux of [$^3$H5]deoxyuridine by almost 90%. This may help maintain high intracellular deoxyuridine nucleotide concentrations and increase uracil misincorporation.

The potentiation of CB3717 toxicity by dipyridamole, via two mechanisms at concentrations achievable in patients, suggests that dipyridamole would be a useful means of enhancing the chemotherapeutic potential of CB3717 in patients.

**Selective radiosensitisation by an antibody-bromodeoxyuridine**

A. Smith, J.A. Gardner, D.M. Tidd & H.M. Warenius

*Department of Radiation Oncology, Clatterbridge Hospital, Bebington, Wirral, Merseyside L63 4JY, UK.*

The application of ricin or ricin A-chain conjugates as selective cytotoxins *in vivo* has proven to be a difficult objective to achieve both because of the affinity of the whole toxins for non-target cells and because of other pharmacokinetic considerations. The substitution of less interactive molecular species in place of ricin may go some way towards resolving these problems and we present here a study on the selective delivery of the radio-sensitising compound 5-bromo-

2′-deoxyuridine to the human adenocarcinoma cell line HT29/5. The route of intra-cellular delivery chosen was by conjugation to transferrin (thereby utilising the transferrin receptor internalisation pathway) or to the AUA1 monoclonal antibody. To increase the loading of the carrier with bromodeoxyuridine without impairing its ability to bind to its cellular target the radiosensitiser was first linked to poly-L-lysine which was in turn conjugated via succinimidyl 4-(*p*-maleimidophenol) to the carrier. The resultant conjugate, when employed *in vitro*, displayed an ability to selectively radiosensitise HT29/5 cells to both photon and neutron irradiation.

### Heat shock and other agents which induce thermotolerance bring about the terminal differentiation of HL-60 human promyelocytic leukaemia cells

F.M. Richards & J.A. Hickman

*CRC Experimental Chemotherapy Group, Pharmaceutical Sciences Institute, Birmingham B4 7ET, UK.*

HL-60 cells will undergo terminal differentiation to mature granulocytes in response to a wide variety of agents. It has previously been shown that the optimum concentration for the induction of differentiation by all the agents was only marginally below the cytotoxic concentration (Langdon & Hickman, *Cancer Res.*, **47**, 140, 1987). This suggests that differentiation induction in HL-60 cells may be an adaptive response to a sub-toxic threat or stress.

HL-60 cells were incubated for 4 days with agents known to induce a stress response and short term resistance to a subsequent stress such as a heat shock (thermotolerance) in other cell types. The local anaesthetic lidocaine (3 mM) induced $48.34 \pm 5.89$ ($n = 4$) percent of cells to differentiate (NBT+). Procaine (5 mM) similarly induced $46.32 \pm 17.57\%$ ($n = 4$) and 6 $\mu$M sodium arsenite induced $30.10 \pm 11.50\%$ ($n = 5$), which was significantly higher than in untreated controls ($2.09 \pm 1.31\%$ $n = 8$).

When HL-60 cells were incubated for 60 min at 43.5°C (heat shocked) followed by a recovery period of 4 days at 37°C, $20.06 \pm 7.29$ ($n = 6$) percent of the cells differentiated. This is the first example of a physical rather than chemical stress inducing differentiation of HL-60 cells. We consider that a stress response may be important in those events leading to the induction of terminal differentiation in HL-60 cells.

### Inhibition by adriamycin of the calcium-mediated ankyrin breakdown in human erythrocytes

K.Y. Tang, M.G. Thompson & J.A. Hickman

*CRC Experimental Chemotherapy Group, Pharmaceutical Sciences Institute, Aston University, Aston Triangle, Birmingham B4 7ET, UK.*

In response to treatment with 5 $\mu$M calcium ionophore (A23187) in the presence of calcium, human erythrocytes underwent calcium-concentration-dependent and time-dependent echinocytosis. This morphological transition could be observed as early as 30 sec after ionophore addition. SDS-PAGE of the human erythrocyte cytoskeleton revealed the breakdown of two cytoskeletal components: ankyrin (Mr 200,000) and band 4.1 (Mr 82,000). However, only ankyrin degradation was rapid enough to account for the rapid onset of calcium-induced echinocytosis. The anthracycline anti-tumour drug, adriamycin, was found to exert protective effect against calcium-induced echinocytosis. In control incubations there were ~70–80% echinocytes after 5 min calcium-loading (150 $\mu$M) at 37°C. In comparison, there were only 15–20% echinocytes when erythrocytes were pre-incubated with 10 $\mu$M adriamycin. This potent inhibition of echinocyte formation by adriamycin was observed at calcium concentrations from 0–150 $\mu$M. At concentrations above 200 $\mu$M, pre-incubation of adriamycin has no significant effect on the morphological transition process compared with the control. SDS-PAGE of isolated erythrocyte membrane vesicles show that adriamycin did not cause significant protein cross-linking. However, adriamycin was found to be capable of inhibiting the calcium-mediated breakdown of ankyrin at calcium concentrations as high as 400 $\mu$M. This inhibition of ankyrin degradation was closely correlated to the ability of adriamycin to protect human erythrocytes against calcium-induced echinocytosis. The erythrocyte membrane and cytoskeleton are widely considered to be an excellent model for other cell types; Adriamycin may, therefore, exert part of its activity at this locus.

### Growth inhibition of a human lung tumour xenograft by selective manipulation of cellular energy metabolism

J. Plumb, R.M. Sri-Pathmanathan & K.C.H. Fearon

*Department of Medical Oncology, University of Glasgow, Glasgow G12 9LX, UK.*

Rhodamine 6G (R6G), a mitochondrial dye, has been shown to act via inhibition of oxidative phosphorylation. Incubation of a human non-small cell lung cancer cell line, WIL, in the presence of R6G (1–10 $\mu$M) resulted in a decrease in the rate of oxygen consumption by the cells by up to 50%. In contrast, the rate of anaerobic metabolism, estimated as the rate of lactate production by the cells, was increased by up to 60% in the presence of R6G (1–10 $\mu$M). The magnitude of both these changes was dose dependent. However, even at a concentration of 10 $\mu$M, R6G had no significant effect on either oxygen uptake or lactate production by freshly prepared rat liver hepatocytes.

The doubling time of the W1L tumour, grown as a xenograft in athymic mice, was increased by about 30% after a single i.p. injection of R6G (2 mg kg$^{-1}$). A single i.p. injection of 5-fluorouracil (50 mg kg$^{-1}$), a known inhibitor of mitochondrial biogenesis, had no effect on tumour doubling time. However, combination of R6G (2 mg kg$^{-1}$) and 5-fluorouracil (50 mg kg$^{-1}$) resulted in an increase in tumour doubling time of greater than 100%.

These results demonstrate a selective inhibition of aerobic metabolism in tumour cells by R6G which may be compensated for, in part, by an increase in the rate of anaerobic metabolism. Inhibition of tumour growth *in vivo* by R6G is enhanced by a non-cytotoxic dose of 5-fluorouracil. This may be the result of an inhibition of tumour cell mitochondrial biogenesis.

### Evidence for polyamine involvement in feeder cell enhancement of tumour cell clonogenicity

A.P. Wilson

*Oncology Research Laboratory, Derby City Hospital, Uttoxeter Road, Derby DE3 3NE, UK.*

The use of feeder cells as a means of improving clonogenicity of tumour cells (TC) is well-documented. Conditioned medium from feeder cells is variable in its ability to enhance clonogenicity of the same cells, and little is known concerning the mechanisms underlying the epithelial – mesenchymal interactions. A model using mesothelial cells

(MC) and an ovarian tumour cell line (TC) has been used to investigate possible mechanisms, and results suggest that polyamines may be implicated. In the model used MC can respond to mitogenic stimulation and clonogenicity of MC can therefore be determined in parallel with TC. Results suggest that the responsiveness of MC to EGF influences interactions between MC, TC, EGF and an amine oxidase inhibitor – iproniazid, (IP). Late passage MC with reduced responsiveness to EGF fail to enhance to growth in the presence of EGF. Stimulation can be restored in the presence of IP which does not itself affect either TC or MC clonogenicity. Conversely early passage MC, which are very responsive to EGF still stimulate to clonogenicity in the presence and absence of EGF. In these circumstances, IP has no effect on enhancement of TC clonogenicity by MC. Experiments are in progress to evaluate the role of polyamine metabolism in more detail.

**Investigations into the mode of action of trimelamol**

I.R. Judson, C.J. Rutty, G. Abel, M. Graham, B.C. Millar[1] & K.R. Harrap

*Drug Development Section and [1]Section of Medicine, Institute of Cancer Research, Sutton, Surrey, UK.*

The mode of action of cytotoxic melamines is unknown. Although the requirement for N-hydroxymethyl groups is well established, whether formed by metabolic activation (HMM, PMM) or chemical synthesis (Trimelamol), there is still little evidence to indicate that DNA alkylation is the cause of cytotoxicity. Trimelamol (T) at 100 $\mu$M for 4 h did not affect calf thymus DNA template activity. Exposure to T at 100 $\mu$M for 2 h caused 46% inhibition of $^3$H-TdR incorporation by PC6 cells *in vitro* but this was completely abolished by semicarbazide (SC) pretreatment (i.e., formaldehyde (F) trapping). However, SC had little effect on the inhibition of growth of PC6 cells *in vitro* by T 125–400 $\mu$M, merely reducing mean inhibition from 62% to 55%. Hence F release contributes little to toxicity in this cell line. Treatment of Balb $C^-$ mice bearing PC6 ascites tumour with T 50 mg kg$^{-1}$ i.v. daily $\times 3$ gave a 77% prolongation in survival. In addition to showing *in vivo* sensitivity, the PC6 tumour proved to be predictive for anti-tumour activity of T in man both in terms of an effective plasma concentration and schedule dependent differences in therapeutic index. Hence the PC6 is a suitable model for mechanistic investigations. A 29% inhibitory effect on $^3$H-TdR incorporation by PC6 ascites cells removed 2 h after T 100 mg kg$^{-1}$ i.v., was virtually absent (5%) by 5 h. Flow cytometric analysis of T-treated PC6 cells failed to show a $G_2$ block suggesting that DNA-DNA *x*-linking is not an important event. Nevertheless T did cause imbalanced growth in PC6 cells at 50–100 $\mu$M *in vitro* as shown by an increase in cell size (70%) and protein content (67%) at 24 h. This was associated with a small reduction in $G_1$ and an increase in S phase cells. Thus T appears to affect the regulation of cell division in this sensitive cell line but the mechanism of this activity remains obscure.

**Effects of N-methylformamide (NMF) on the cell cycle of murine TLX5 lymphoma cells *in vitro*: Characterization of a reversible quiescent $G_1$ phase substate**

C.A. Bill, A. Gescher & J.A. Hickman

*CRC Experimental Chemotherapy Group, Pharmaceutical Sciences Institute, Aston University, Birmingham B4 7ET, UK.*

NMF is one of a number of polar solvents which can induce the terminal differentiation of certain malignant cell lines *in vitro*. We have previously demonstrated an NMF concentration-dependent decrease in the growth rate and an associated $G_1$ phase accumulation of murine TLX5 lymphoma cells *in vitro* (Bill *et al*, *Br. J. Cancer*, **54**, 168, 1986). In this study we investigated further the $G_1$ phase sub-compartments of TLX5 cells incubated with NMF *in vitro* and assessed whether or not terminal differentiation had resulted from the drug exposure. Simultaneous DNA and RNA analysis of TLX5 cells treated with NMF *in vitro* by flow cytometry revealed a concentration-dependent decrease in cellular RNA content, indicative of an early $G_1$ phase arrest. TLX5 cells exposed to 170 mM NMF for 48 h resulted in 99.7% of the $G_1$ population in the subcompartment with the lowest RNA content, termed $G_{1A}$, whereas for TLX5 cells at plateau phase for 3 days $G_{1A}$ contained 88.5% of $G_1$ phase cells. TLX5 cells were incubated with 106 mM NMF for 48 h, cells were then washed free of the drug and cultured in fresh medium. There was an initial fall in viability (82$\pm$3% to 45$\pm$4%) as measured by trypan blue exclusion and rise in the viable $G_1$ phase population (77% to 95%) 24 h after NMF removal. However, by 72 h a normal control cell cycle distribution was evident. Anchorage independent growth analysis of these cells in soft agar, plated immediately after NMF was removed from the medium gave a clonogenic efficiency of 54.5$\pm$4.3% of the control value, indicating complete proliferative recovery of the viable cell population. We conclude that murine TLX5 lymphoma cells exposed to NMF *in vitro* are not terminally differentiated, but reside in a quiescent substate which was reversed on drug removal.

**N-methyldeuteroformamide, an antineoplastic isotopomer of N-methylformamide with markedly reduced toxicity**

D. Chubb, M.D. Threadgill & A. Gescher

*CRC Experimental Chemotherapy Group, Pharmaceutical Sciences Institute, Aston University, Birmingham B4 7ET, UK.*

Results of clinical trials of N-methylformamide ($OHCNHCH_3$, NMF) indicate that its therapeutic potential is low. The major toxicities of NMF in patients were nonspecific malaise and liver damage. During investigations on the formyl-deuterated isotopomer of NMF ($ODCNHCH_3$, D-NMF) a primary kinetic isotope effect on the metabolism of NMF to methylamine and N-acetyl-S-(N-methylcarbamoyl)cysteine (Threadgill *et al*, *Br. J. Cancer*, **54**, 193, 1986) was discovered. In order to establish whether this metabolic pathway is involved in the mechanism of the antitumour activity of NMF, the efficacy of D-NMF was tested against the M5076 reticulum cell sarcoma grown in female $BDF_1$ mice. The compound was administered i.p. daily for 17 consecutive days and tumour volumes were measured at regular intervals. D-NMF at 100 mg kg$^{-1}$ was inactive and at 200 mg kg$^{-1}$ it afforded a T/C value of 28 (mean of 5 mice), which is almost identical to the activity observed with 100 mg kg$^{-1}$ NMF. At a dose of D-NMF of 400 mg kg$^{-1}$, tumour volume was not measurable. The difference between D-NMF and NMF in toxicity was most dramatic. At 800 mg kg$^{-1}$ D-NMF did not cause weight loss, whereas 300 mg kg$^{-1}$ of NMF was near the $LD_{50}$ in $BDF_1$ mice. Two conclusions can be drawn from these results: (i) Cleavage of the formyl C-H bond appears to be a prelude to the generation of both the antitumour activity and toxicities of NMF. (ii) The startling reduction of toxicity on substitution of the formyl H with D indicates that the dose-limiting toxicity of NMF in mice is possibly mediated by processes other than or additional to the hepatic lesion.

### Characterisation of the growth of human promyelocytic HL-60 cells implanted into millipore chambers *in vivo*

L. Hughes, D. Chubb & J.A. Hickman

*CRC Experimental Chemotherapy Group, Pharmaceutical Sciences Institute, Aston University, Birmingham B4 7ET, UK.*

A number of *in vitro* systems have been used to examine the potential of a variety of agents to induce the terminal differentiation of malignant cells. Few attempts have been made to conduct such experiments under conditions of appropriate drug pharmacodynamics *in vitro*, and fewer have utilised an *in vivo* assay of drug-induced terminal differentiation. We have investigated the growth characteristics of the human promyelocytic leukaemia HL-60 cells when they were implanted in millipore chambers, into 20 g, female CBA/CA mice. Growth characteristics in chambers were dependent on a number of variables. When 150 $\mu$l of $1 \times 10^6$ HL-60 cells ml$^{-1}$ in RPMI 1640 medium ($\pm$10% foetal calf serum), which had an *in vitro* doubling time of 24 h, were implanted in chambers, with a pore size of 0.45 $\mu$m, immediate and rapid growth (doubling time = 8 h) was observed when the chambers were adjacent to the incision wound (final cell density = $1.3 \times 10^7$ cells ml$^{-1}$ on day 2). When distal from the wound site a growth lag of 72 h was observed, followed by log. phase growth (doubling time = 24 h) to plateau at 168 h at $2 \times 10^7$ cells ml$^{-1}$. Under these conditions, growth characteristics were optimally reproducible when an initial cell density of $1 \times 10^6$ cells ml$^{-1}$ and a 0.45 $\mu$m Millipore filter was used. In the absence of serum in the chamber, spontaneous differentiation, assessed by biochemical and morphological tests, was <10% in cells which had been implanted 22 days earlier. We consider this to be a potentially useful model for assessing drug-induced terminal cell differentiation *in vivo*.

### Anti-tumour activity of TCNU in transplantable colon tumours in NMRI mice

M.C. Bibby & J.A. Double

*Clinical Oncology Unit, University of Bradford, Bradford, West Yorkshire BD7 1DP, UK.*

1-(2-chloroethyl) 3-[2-(dimethylaminosulphonyl) ethyl]-1-nitrosourea (TCNU) is a new nitrosourea with greater water solubility. Following improved activity over other nitrosoureas in experimental tumour systems it has undergone Phase I evaluation. Objective responses have been seen in squamous cell, adenocarcinoma and large cell carcinoma of the lung as well as in mesothelioma and breast cancer. Phase II evaluation in non-small cell lung cancer, melanoma, breast cancer and colorectal carcinoma are now in progress. The purpose of this study was to examine its activity in a panel of transplantable adenocarcinomas of the mouse colon with varying sensitivities to standard nitrosoureas. Chemosensitivity was assessed in three tumour lines, the ascitic line MAC 15A, a poorly differentiated subcutaneous solid tumour MAC 13 and a well differentiated solid tumour MAC 26. Effects of treatment were determined as previously described (Double *et al*, *Br. J. Cancer*, **54**, 595, 1986). Antitumour activity was also assessed in systemic tumours produced by i.v. inoculation of MAC 15A cells. TCNU was active against all tumour lines. This represents an improvement over standard nitrosoureas. Response of MAC 13 was similar to those seen with standard nitrosoureas whereas improved responses were seen in MAC 15A and MAC 26. The activity against MAC 26 was particularly interesting as this tumour is unresponsive to standard nitrosoureas. These latter improvements may indicate that the water solubility of TCNU may alter pharmacokinetics which may have therapeutic implications. Tissue distribution and pharmacokinetic investigations are currently in progress.

### Antitumour screening models relevant to the development of platinum drugs

K.R. Harrap, M. Jones, P.M. Goddard, R.M. Orr & Z.H. Siddik

*Drug Development Section, The Institute of Cancer Research, Sutton, Surrey, UK.*

Carboplatin (CBDCA, JM8, Paraplatin) was developed in the expectation that it would possess clinical activity comparable to that of the parent drug, *cis*-platin, yet be much better tolerated [Harrap K.R., *Cancer Treat. Rev.*, **12**, (Supp A), 21 1985]. It was not expected to possess activity in *cis*-platin-resistant disease, since only platinum complexes containing a diaminocyclohexane ligand possessed this property in preclinical screening models [Burchenal, J.H. *Rec. Res. Cancer Res.*, **4**, 146, 1980]. The lowered clinical toxicity of carboplatin has been demonstrated [Calvert, A.H. *et al*, *Cancer Chemother. Pharmacol.*, **9**, 140, 1982], while also the drug is active in a small, yet significant, proportion of ovarian cancer patients who have relapsed on previous *cis*-platin therapy [Wiltshaw, E. *Cancer Treat. Rev.*, **12**, (Supp A), 67, 1985]. Further, the spectrum of clinical activities possessed by carboplatin may not coincide with that of *cis*-platin (Cannetta *et al*, *Cancer Treat. Rev.*, **12**, (Supp A), 125, 1985). These findings suggest that more effective platinum derivatives may be discovered with suitably predictive screening models. The limitations and disparate predictiveness of widely used experimental tumours (L1210 and ADJ/PC6 plasmacytoma) and their *cis*-platin-resistant variants are presented in the light of the established clinical requirements. Preliminary studies with a number of human ovarian tumour xenografts grown in the nude mouse suggest that a panel of such tumours, characterised for sensitivity against appropriate reference platinum drugs (*cis*-platin, carboplatin, iproplatin, tetraplatin), may be of greater utility in the development of novel platinum agents.

### Relationship between pharmacokinetics and stability of TCNU and *in vitro* sensitivity of mouse colon tumour cells

R.M. Phillips, P.M. Loadman, M.C. Bibby & J.A. Double

*Clinical Oncology Unit, University of Bradford, Bradford, West Yorkshire BD7 1DP, UK.*

Preliminary studies in this laboratory with 1-(2-chloroethyl) 3-[2-(dimethylaminosulphonyl)ethyl]-1-nitrosourea (TCNU) have been shown to be highly active against transplantable mouse colon tumours (MAC). *In vivo* pharmacokinetics and *in vitro* stability of TCNU have been studied using a reverse phase HPLC technique giving a sensitivity of 5 ng ml$^{-1}$. Rate of breakdown of TCNU at 37°C *in vitro* in the dark was 0.14 g min$^{-1}$ in mouse plasma, 0.08 g min$^{-1}$ in human plasma, 0.10 g min$^{-1}$ in PBS and 0.11 g min$^{-1}$ in RPMI 1640 with 10% foetal calf serum. Rate of breakdown in 0.9% saline at 20°C on the bench was 0.02 g min$^{-1}$. Non tumour bearing NMRI mice were injected i.p. with therapeutic doses of TCNU in 0.9% saline and plasma profiles measured between 0–4 h. TCNU was not detectable in the plasma at 2 h. *In vitro* cell lines were derived from 3 transplantable MAC tumours of differing growth characteristics and histology MAC 13, MAC 15A and MAC 26. Chemosensitivity to TCNU was determined at a range of concentration and exposure times for each of these lines using a modified clonogenic assay (Hamburger & Salmon, *Science*, **187,** 461, 1977) and cytotoxic effects of treatment were expressed in terms of percentage survival. Drug levels within *in vivo* achievable plasma concentrations were employed. All 3 cell lines responded to TCNU and the spectrum of chemosensitivity was similar to that previously seen *in vivo* with MAC 13 being the most sensitive and MAC 26 being less responsive. This study demonstrates that *in vitro* chemosensitivity using a clonogenic assay at relevant biologically achievable concentrations predicts for *in vivo* activity of TCNU in 3 histologically different transplantable mouse colon tumours.

### Characterisation of a *cis*-platin-induced DNA damage in a human bladder tumour continuous cell line

S.A. Shellard[1], P. Bedford[1,2], M.C. Walker[2], J.R.W. Masters[2] & B.T. Hill[1,2]

[1]*Laboratory of Cellular Chemotherapy, Imperial Cancer Research Fund, London WC2A 3PX and* [2]*Institute of Urology, St Paul's Hospital, London WC2H 9AE, UK.*

Two sublines have been derived from a human bladder carcinoma cell line (RT112-P) by either continuous exposure to *cis*-platin (RT112-CP+) or to fractionated X-irradiation (RT112-DXR$_8$). *In vitro* sensitivities to *cis*-platin were determined by clonogenic assay following a 1 h exposure to drug. The RT112-DXR$_8$ subline exhibited a 1.6-fold hypersensitivity and the RT112-CP+ subline a 2-fold resistance to *cis*-platin compared to the parent cells (IC$_{50}$ for RT112-P is 1.1 $\mu$g ml$^{-1}$). Similar patterns of cross-resistance and collateral sensitivity to carboplatin and iproplatin were also observed. Since DNA is considered an important target for the cytotoxic action of *cis*-platin, DNA-DNA interstrand crosslinking and single-strand breakage were quantitated in these lines at 0, 5, 14 and 24 h, following a 1 h *in vitro* exposure to drug, by the technique of alkaline elution. Interstrand crosslinks were induced to a far greater extent in the sensitive subline and to a similar extent in the resistant subline, when compared to the parent, with peak levels of 126.5, 52.8 and 58.2 rad-equivalents respectively. A significantly increased uptake of $^{195m}$*cis*-platin was noted in the sensitive subline with a similar uptake in the resistant line compared to the parent cells, whereas the extent of binding to DNA was comparable in all three lines. Significantly increased levels of both glutathione and glutathione reductase occurred in the RT112-CP+ cells compared to the parent and RT112-DXR$_8$ cells, which had similar levels. Therefore, the underlying mechanisms of resistance or collateral sensitivity to *cis*-platin may not be conversely related.

### The effect of mitozantrone on viability and differentiation of human promyelocytic leukaemia (HL60) cells

L.H. Patterson, M. Ruffet, M.A. Aulton, J.K. Sugden & M.J. Taylor

*School of Pharmacy, Leicester Polytechnic LE3 9BH, UK.*

The effect of mitozantrone on HL60 cells was investigated since this drug is indicated in the treatment of certain myelogenous leukaemias. HL60 cells (3 x 10$^5$ ml$^{-1}$) in log phase growth maintained in RPMI 1640 medium supplemented with foetal calf serum (10%) were incubated at 37°C in the presence of 0.1 $\mu$M mitozantrone for 24 h. The cells were then washed to remove drug and incubated for a further 4 days in fresh medium. Induction of differentiation was monitored by assessment of phagocytosis of compliment-coated yeast cells and phorbol ester mediated superoxide anion production as measured by nitrobluetetrazolium (NTB) reduction. The results shown in the table which are typical of 2 separate experiments indicate that mitozantrone produces a progressive inhibition of HL60 cell survival even after the drug is removed (days 2 and 5) from the incubation medium. Furthermore mitozantrone has promoted differentiation in 14–28% of the remaining cells. These results are consistent with the concept that the cytotoxic threat to HL60 cells is sufficient to induce differentiation.

| *Treatment* | *Incubation time (days)* | *Viable cells* ($\times 10^5$) | % *Viability* | % *NBT reduction* | % *Phagocytosis* |
|---|---|---|---|---|---|
| None | 1 | 3.5 | 95 | 1 | 1 |
| | 2 | 7.2 | 94 | 1 | 1 |
| | 5 | 7.6 | 92 | 2 | 3 |
| Mitozantrone | 1 | 1.6 | 66 | 5 | 4 |
| (0.1 $\mu$M) | 2 | 0.4 | 22 | 28 | 14 |
| | 5 | 0.1 | 7 | 14 | 25 |

### Monitoring of exposure to alkylating agents by analysis of N-terminal protein adducts

P.B. Farmer, E. Bailey & B.J. Passingham

*MRC Toxicology Unit, Woodmansterne Road, Carshalton, Surrey SM5 4EF, UK.*

The interaction of proteins with alkylating carcinogens frequently results in the formation of covalent bound adducts of the carcinogen with specific nucleophilic sites in amino acids. Amongst these sites is the amino group of the N-terminal amino acid, an example of the reaction being the formation of N-(2-hydroxyethyl)valine at the N-terminal valine of haemoglobin following exposure to ethylene oxide. This adduct has been quantified in human haemoglobin by Törnqvist *et al.*, (*Anal. Biochem.*, **154,** 255, 1986) following its conversion to a penta-fluorophenylthiohydantoin derivative by a modified Edman degradation procedure. We have now modified this analytical approach and applied our method to the determination by GC-MS of hydroxyethylvaline in the globin of human subjects. The method involves the following steps: 1. Addition of stable isotope labelled internal standard (globin treated with d$_4$-ethylene oxide); 2. Reaction of globin (50 mg) in formamide solution with pentafluorophenyl isothiocyanate; 3. Extraction of N-(2-hydroxyethyl)valine pentafluorophenylthiohydantoin; 4. Derivatisation with a trimethylsilylating agent; 5. Electron impact capillary GC-MS using selected ion recording of m/z 440 and 444. The sensitivity of the method is sufficient to detect 'background' hydroxyethylvaline in control subjects of $<100$ pmol g$^{-1}$ globin and is currently being used to measure levels of the adduct in smokers, (who are exposed to ethylene oxide through the inhalation of ethene), and in non-smokers.

### The effect of blood transfusion on tumour spread

P.J. Clarke & D. Tarin

*Nuffield Department of Pathology, (University of Oxford), John Radcliffe Hospital, Oxford OX3 9DU, UK.*

Routine blood transfusion in patients awaiting cadaver renal transplantation has improved graft survival rates at one year. Although the immunological mechanisms involved remain unclear, there is concern that, if a similar process occurs in transfused cancer patients, this may be reflected in a poorer prognosis. Retrospective clinical studies have supported this theory.

The effect of blood transfusion was therefore studied using 3 transplantable mouse tumour lines (UV-2237, KHT and B16-F10). On day −14, animals were transfused with 0.25 ml of allogeneic blood. Tumour inoculation into the appropriate syngeneic host was performed on day 0 and subcutaneous tumour growth was assessed by weekly measurements. Metastatic spread of tumour to the lungs and other organs was determined at autopsy and histologically.

There was no difference in tumour growth rate in those animals which were transfused compared with saline infused controls. However there was an increase in the number of pulmonary metastases in certain, but not all, of the transfused groups. If a similar phenomenon occurs in man, these findings may have important implications.

### New B lymphoblastoid parents for human fusion

H.Y. Youd, S. Speirs & H.M. Warenius

*Department of Radiation Oncology, Clatterbridge Hospital, Bebington, Wirral, Merseyside L63 4JY, UK.*

One of the main problems concerned with the production of human monoclonal antibodies has been the limited availability of a suitable fusion partner. Past problems include low Ig production, low fusion success rate, and instability of hybrids. We are concerned with the production of alternative fusion partners and comparing them with other lines used in Human X Human B lymphocyte fusions. Using lymphocytes taken from a patient with plasma cell leukaemia (PCL) at plasmaphoresis, we attempted to obtain better immortal lines by growing the cells in nude mice, which up to the present has been unsuccessful, but by hybridisation with UC729-6 four cell lines have been produced. The hybrid nature of the cells have been confirmed by examination of the ploidy on a FACS 420. Tissue typing for histocompatibility antigens showed in addition to the presence of antigens associated with the UC729-6, those of the plasma cell leukaemia. Surface and cytoplasmic immunoglobulin characteristics have been examined using the FACS and enzyme linked immunoabsorbent assay. All cell lines produced IgA, lambda of the PCL parent and varying levels of IgM, known to be produced by UC729-6. The cloning, plating efficiency and growth characteristics in comparison of UC279-6 and LICR-LON-HMY2, are much improved. These new lines have been in culture for 18 months, can be preserved in liquid nitrogen and recovered with high viable cell yield; they are now being used a fusion partner to produce hybrids with spleen cell and from a patient with idiopathic thrombocytopenic purpura and lymph node cells from patients with colonic cancer.

### Interferon (IFN) modulation of MHC antigen expression and cytostasis on human tumour cell lines *in vitro*

R.K. Iles[1], C. Navarrete[2], R.T.D. Oliver[1] & H. Festenstein[2]

[1]*Medical Oncology Unit, Department of Urology and* [2]*Department of Immunology, The London Hospital Medical College, London E1, UK.*

Fourteen cell lines: 12 of genitourinary tumour origin and 2 from normal or SV40 immortalised skin were studied for their major histocompatibility complex (MHC) antigen expression and subsequent modulation by IFN$\alpha$ and IFN$\gamma$. The cytostatic effect of the IFNs on these cell lines was also measured.

In order to quantify MHC antigen expression, as detected by monomorphic class I and class II monoclonal antibodies (W6/32 and EDU1), three methods were used: Fluorescent activated cell sorter (FACS), enzyme linked immunosorbant assay (ELISA) and *in situ* immunofluorescence microscopy. Results from all 3 were combined to give more representative estimates. Cytostasis was measured as relative %$^3$H-thymidine incorporation compared to untreated controls.

The results show heterogeneous MHC antigen expression and cytostasis responses to both IFNs between the different cell lines. When these two parameters were compared, we found a strong correlation between class II expression responses and cytostasis, i.e. strong class II induction – positive cytostasis and *vice versa*. In contingency table analysis (Fisher's exact test) $P<0.05$ and $P<0.01$ for IFN$\alpha$ and IFN$\gamma$ respectively.

These results indicate for all the cell lines studied, alterations in IFN cellular kinetic pathways, which are common for these two biological phenomena, may result in diminished responsiveness to IFN modulation, particularly for IFN$\gamma$ which induces the expression of class II antigens

It can be postulated that the down regulation of IFN induced responses may be important in the development of neoplasia. However it is also possible that the results occur as a consequence of the neoplasia.

### Monoclonal antiidiotypic antibodies and immune responses to tumours

P. Dunn, O. Leger, C. Johnson, S. Pease, J. Styles & C. Dean

*Institute of Cancer Research, Block X, Clifton Avenue, Sutton, Surrey, UK.*

The Hooded rat sarcoma HSN metastasises to the lungs when grown in immune suppressed hosts. In immunocompetent syngeneic rats spontaneous metastasis is rare and growth of the tumour elicits the production of antibodies directed against a 180 kD surface protein. Hybridomas secreting specific monoclonal antibodies of differing isotype have been prepared from tumour bearers. All of the monoclonal antibodies obtained compete with each other for binding to HSN cells.

Monoclonal antiidiotypic antibodies ($Ab_2$) directed against combining site idiotopes of two of the specific antibodies have been prepared by immunising syngeneic rats with the tumour specific $Ab_1$. The antiidiotypes obtained (HIM/1/230 and HIM/8/88) are individually specific for the immunising antibodies 11/160, $\gamma_{2b}$ or ALN/11/53, $\gamma_{2a}$). We conclude that there are two overlapping epitopes on the 180 kD antigen and these elicit antibody responses in tumour bearers that are isotype specific. One of the antiidiotypes (HIM/1/230, $\gamma_{2a}$) has been used to 'vaccinate' naive Hooded rats. The animals generate antibodies ($Ab_3$) which include those with specificity similar to the $Ab_1$ (11/160) isolated from tumour bearers. The vaccinated rats show resistance to a subsequent challenge with tumour.

**DNA cross-linking and platinum resistance in ovarian carcinoma cell lines**

G.J. Beattie, I.P. Hayward & J.F. Smyth

*Medical Oncology Unit, Department of Clinical Oncology, Western General Hospital, Edinburgh EH4 2XU, UK.*

The difference in sensitivity to *cis*-platinum (*cis*-DDP) between 2 human ovarian adenocarcinoma cell lines established from ascites samples from one patient before (PE01) and after (PE04) the onset of clinical resistance to the drug was studied using the technique of alkaline elution. After 5 h of treatment with 50 $\mu$M *cis*-DDP no significant difference in total cross-linking as expressed in rad equivalents was observed between the two cell lines. There was however a 2–3 fold difference in protease-resistant cross-linking between PE01 and PE04; see table.

| | *Total cross-linking* | *Protease-resistant cross-linking* |
|---|---|---|
| PE01 | 468 (±35) | 231 (±23) |
| PE04 | 422 (±32) | 97 (±8) |

These figures are in agreement with the 3-fold difference in sensitivity obtained in clonogenic assays using these cell lines. Protease-resistant cross-linking showed a linear-dose response as measured 4 h after a 2 h dose of 5–50 $\mu$M *cis*-DPP. Here a 2-fold difference in protease-resistant cross-linking was observed over the range studied. A subline derived from PE01 (PE01 *cis*-DPP$^R$) with 25-fold resistance to *cis*-DPP acquired *in vitro* showed even less protease-resistant cross-linking than either PE01 or PE04. These data suggest that the same total amount of platinum/DNA adducts occur in PE01 and PE04 but DNA-interstrand cross-links are reduced in PE04 and further reduced in PE01 *cis*-DDP$^R$. This would implicate either specific DNA lesions or particular DNA repair pathways in the resistance observed. This work is being extended to study DNA damage *in vivo* in peripheral blood lymphocytes from patients with ovarian carcinoma currently being treated with *cis*-platinum.

**Quantitation of DNA adducts caused by chemotherapeutic drugs using monoclonal antibodies**

M.J. Tilby, J.M. Styles, C.A. Johnson, R. Knox, J.J. Roberts & C.J. Dean

*Institute of Cancer Research, Block X, Clifton Avenue, Belmont, UK.*

We have produced a number of monoclonal antibodies that bind to DNA that has been alkylated with melphalan but not to normal DNA. One of these has been used in a sensitive enzyme-linked immunoabsorbent assay (ELISA) to detect DNA damage caused by therapeutic doses of this drug. We present data showing that the sensitivity of this technique permits detection of less than 1 adduct in $10^6$ bases and that the assay correlates with the less sensitive radiochemical detection of melphalan adducts. We are using these antibodies to study the formation and repair of DNA lesions in lymphocytes of patients undergoing melphalan therapy and to study the distribution of DNA damage in various cell types.

More recently we have produced monoclonal antibodies that bind to DNA that has been modified with *cis*-platinum and these are being used in similar studies.

**Reactivity of 3-substituted imidazotetrazinones towards DNA, RNA and protein**

V.L. Bull & M.J. Tisdale

*CRC Experimental Chemotherapy Group, Pharmaceutical Sciences Institute, Aston University, Aston Triangle, Birmingham B4 7ET, UK.*

The reactivity of mitozolomide and the 3-methyl (CCRG81045) and 3-ethyl (CCRG82019) analogues, towards DNA, RNA and protein has been investigated both with the isolated macromolecules and in cells sensitive (GM892A, Mer$^-$) or resistant (Raji, Mer$^+$) to drug action. The extent of total alkylation of all three macromolecules increases as the concentration of drug increases, and towards isolated DNA and RNA the extent of alkylation by CCRG81045 is about twice that of mitozolomide, and both are 5 to 10 times more reactive than CCRG82019. Mitozolomide is about 10-fold more reactive towards isolated protein than the other two imidazotetrazinones. When macromolecules are isolated from cells treated with these agents, the relative extents of alkylation differ from that observed with isolated macromolecules. For all three macromolecules in both cell lines, the levels of alkyl adducts remaining 24 h after mitozolomide treatment exceeds that of the other two agents. While the levels of alkyl adducts remaining on DNA and RNA in cells after CCRG81045 and CCRG82019 treatment approximates to that seen with isolated macromolecules, there are more chloroethyl adducts remaining 2 h after mitozolomide treatment (0.1 mM) on DNA in intact cells (0.48 pmol drug bound $\mu g^{-1}$ DNA) than on isolated DNA (0.12 pmol drug bound $\mu g^{-1}$ DNA). The levels of alkyl adducts remaining on DNA and RNA after treatment with all three agents is greater in Mer$^-$ than Mer$^+$ cells, but there is no difference in the levels of alkyl adducts on protein. These results suggest that the inability to repair specific base alkylations may be responsible for the selective cytotoxicity of the imidazotetrazinones.

**Expression of cytochrome P450S in tumour-derived cell lines**

L.A. Stanley[1], A.R.R. Scott[1], N. Spurr[2] & C.R. Wolf[1]

*Imperial Cancer Research Fund, [1]Molecular Pharmacology Laboratory, Hugh Robson Building, George Square, Edinburgh and [2]Human Genetic Resources Laboratory, Clare Hall, Potter's Bar, Hertfordshire, UK.*

The level of drug-metabolising enzymes in a tumour is important in determining its response to chemotherapy. Regulation of cytochrome P450 (P450) expression by drugs and endogenous factors (e.g. interferons (Ifns), interleukins (ILs), and tumour necrosis factor (TNF)) is also potentially important where chronic inflammation is associated with exposure to carcinogens (e.g. in the smoker's lung). In order to select a cell culture model to examine this problem, constitutive P450 expression in several cell lines has been studied immunochemically, along with regulation of this expression by P450 inducing agents such as phenobarbital, dexamethasone, Aroclor 1254, and 1,2-benz-anthracene. The results show that the human tumour-derived cell lines HepG2 (hepatoma), NCI-H322 (non-small cell lung carcinoma), MCF-7 (breast carcinoma) and LS174T (colon carcinoma) express the P450 isozyme $MC_{1b}$. This enzyme has high activity in the metabolism of a wide variety of drugs and chemical carcinogens. The above cell lines will therefore be of value in studying the effects of inflammatory mediators such as Ifn$\gamma$, IL-1, and TNF on P450 expression and elucidating the role of P450 $MC_{1b}$ in the disposition of carcinogens and chemotherapeutic drugs.

### Elevated expression of glutathione-S-transferase subunits in granulocytes following drug priming

C.R. Wolf[1], A.D. Lewis[1] & J.D. Hayes[2]

[1]*Imperial Cancer Research Fund, Laboratory of Molecular Pharmacology and Drug Metabolism, George Square, Edinburgh EH8 9XD and* [2]*Department of Clinical Chemotherapy, Edinburgh Royal Infirmary, Edinburgh, UK.*

We have studied the response of bone marrow cells to cytotoxic drugs as a model for evaluating the regulation of glutathione-dependent enzymes as stress response proteins. The model chosen is based on the observation that when mice are dosed with a low 'priming' dose of cyclophosamide, they become resistant to a subsequent potentially lethal dose given 5 to 6 days later (Millar *et al*, *Br. J. Cancer*, **32,** 193, 1975). We have shown previously that both glutathione (Gsh) and glutathione-S-transferases (Gst) (which have been implicated in the detoxification of the cytotoxic metabolites of cyclophosamide) are increased at the points of maximum protection. We have now extended this study to identify the Gst isoenzymes affected. SDS/PAGE analysis of bone marrow proteins following 'priming' with cyclophosamide has shown an elevation of a polypeptide that comigrated with rat YbYb Gst subunit. An elevation in levels of a protein equivalent to the neutral YbYb subunit was confirmed by Western blot experiments. A slight elevation in the basic (YaYa) subunit was also observed. The elevation in the YbYb subunit was shown to occur within the granulocyte cell population and could be seen in both peripheral cells as well as those from the bone marrow. Lymphocytes in contrast, contained high levels of acidic YfYf and low levels of YbYb subunits. The levels of the Gsts in these cells were unchanged following priming. In view of the importance of these enzymes in the protection of cells from cytotoxic electrophiles, e.g. cancer chemotherapeutic agents, the study of the Gst gene expression may provide information about factors which regulate drug resistance in tumour and normal cells.

### Expression of glutathione-S-transferases in tumour cell lines

A.D. Lewis[1], J.D. Hayes[2] & C.R. Wolf[1]

[1]*Imperial Cancer Research Fund, Laboratory of Molecular Pharmacology and Drug Metabolism, George Square, Edinburgh EH8 9XD and* [2]*Department of Clinical Chemotherapy, Edinburgh Royal Infirmary, Edinburgh, UK.*

Glutathione and glutathione-dependent enzymes such as the glutathione-S-transferases (Gsts) play a central role in the protection of cells from cytotoxic and carcinogenic compounds. However, relatively little is known about the glutathione levels and Gst expression in tumour cells. Nevertheless, there is evidence which indicates that it is an important factor in tumour cell susceptibility to anticancer drugs. As a consequence the expression of Gst in 10 tumour cell lines of different origin has been studied. A large variation in Gst activity, determined using CDNB as a substrate was measured. The human breast cancer cell line MCF-7 had extremely low activity and was ~100 fold less than either the ovarian cell line PE-04 or the non-small cell lung carcinoma line H358. Western blots of soluble fractions from these lines using antibodies raised against human Gsts basic ($\varepsilon$, YaYa), neutral ($\mu$, YbYb) and acidic ($\lambda$, YfYf) showed high levels of the acidic YfYf poltpeptide (an enzyme which has been associated with drug resistance) in most of the cell lines studied. Ovarian adenocarcinoma cell line PE-04, lung carcinoma line H358 and the human bladder carcinoma line EJ contained highest levels of this enzyme. Mouse hepatoma line HEPA 1 contained extremely low amounts of the YfYf subunit and no visible band was seen in the MCF-7 cells. No basic YaYa Gst was found at significant levels in any of these cell lines. Neutral enzyme YbYb was only detected in MCF-7, HEPA 1 and foetal fibroblasts. Interestingly the data shows that different tumour cell lines have marked differences in their Gst isoenzyme expression and levels. The possibility exists therefore that these variations are related to the susceptibility of tumours to chemotherapy.

### Cross-resistance of a daunorubicin resistant cell line towards anthrapyrazolone

D.G. Poppitt, A.T. McGown & B.W. Fox

*Paterson Institute for Cancer Research, Christie Hospital and Holt Radium Institute, Manchester M20 9BX, UK.*

The anthrapyrazole class of intercalating agents have been developed in order to reduce free radical production associated with the anthraquinone anti-tumour drugs. Two P388 cell lines resistant to daunorubicin have been developed from the parental cell line by incremental challenge with the drug *in vitro*. Growth inhibition studies on these cell lines show cross resistance towards anthrapyrazolone. The $ID_{50}$ values (concentration of drug required to cause a 50% decrease in cell growth) are shown in the table. Resistance to daunorubicin in these cell lines has been shown to be associated with a 4-fold decreased cellular accumulation of drug.

| *Cell line* | $ID_{50}$ *daunorubicin* | $ID_{50}$ *anthrapyrazolone* |
|---|---|---|
| P388 | $19 \times 10^{-9}$ M | $8 \times 10^{-9}$ M |
| P388R8/13 | $6.5 \times 10^{-7}$ M | $7 \times 10^{-7}$ M |
| P388R8/22 | $2.7 \times 10^{-6}$ M | $1.8 \times 10^{-6}$ M |

(McGown *et al*, *Cancer Chemother. Pharmacol.*, **11,** 113, 1983). Biochemical extraction of anthrapyrazolone following treatment (1 h; 37°C) from the parental and resistant cell lines however shows no difference in drug accumulation. Co-incubation with verapamil (10 $\mu$m), which causes the resistant cells to incorporate daunorubicin to the same level as the parental cells, causes a small (~20%) but significant ($P<0.05$) increase in athrapyrazolone accumulation in each of the cell lines tested.

Resistance to daunorubicin in these cell lines is accompanied by cross resistance to anthrapyrazolone. However, no differential drug accumulation could be observed between the parental and resistant cell lines.

### Inhibition of adriamycin superoxide formation and lipid peroxidation by the anthrapyrazolone CI941

M.A. Graham[1], D.R. Newell[1] & L.H. Patterson[2]

[1]*Drug Development Section, Institute of Cancer Research, Sutton, Surrey and* [2]*The School of Pharmacy, Leicester Polytechnic, UK.*

Reactive free radical formation and the subsequent induction of lipid peroxidation have been implicated in the serious dose limiting cardiotoxicity of adriamycin (Myers *et al*, *Science*, **197,** 165, 1977). Adriamycin can undergo a one electron reduction to form a drug free radical which can react directly with molecular oxygen to form superoxide ($O_2^-$) and thence other activated oxygen species such as the hydroxyl radical OH• and $H_2O_2$. These activated oxygen species can in turn initiate lipid peroxidation and other destructive cellular events such as membrane damage, enzyme inactivation and DNA strand cleavage. The anthrapyrazole CI941 is one of a new series of DNA complexing drugs which have demonstrated high level antitumour activity against a broad spectrum of murine tumours (Leopold *et al*., *Cancer Res*., **45**, 5532, 1985) and is currently undergoing phase I clinical evaluation. We have examined the effect of CI941 on adriamycin free radical generation, superoxide formation and lipid peroxidation, using rat liver microsomes fortified with NADPH. The results show that CI941 can inhibit adriamycin (50 μM) induced NADPH consumption (56% inhibition at 50 μM CI941). Electron spin resonance studies have shown that CI941 can diminish the adriamycin drug free radical signal in a dose dependent manner. Similarly adriamycin superoxide formation (using purified cyt. $P_{450}$ reductase) can be inhibited by CI941 (IC50 = 75 μM). An examination of the effects of the two agents on lipid peroxidation reveals that CI941 is a potent inhibitor of adriamycin stimulated lipid peroxidation (IC50 = 5 μM). In conclusion therefore, the inhibitory effect of CI941 on adriamycin activation may form the basis for the combined use of the two drugs in attempts to diminish adriamycin cardiotoxicity.

**Interaction of doxorubicin and mitozantrone with myoglobin**

T.J. Lavelle & L.H. Patterson

*School of Pharmacy, Leicester Polytechnic, Leicester LE1 9BH, UK.*

The interaction of doxorubicin and mitozantrone with myoglobin has been investigated to establish whether this haemoprotein could be involved in the cardiotoxic actions of these antitumour agents. Oxymyoglobin ($MbFe(II)0_2$) and metmyoglobin (MbFe(III)) were prepared as described by Taylor & Hochstein, (*Biochem. Pharmacol*., **27**, 2079, 1978) and the effects of doxorubicin and/or mitozantrone on them monitored spectrophotometrically between 450–650 nm. Doxorubicin was shown to stimulate autooxidation of $MbFe(II)0_2$ to MbFe(III) (basal rate typically 100 pmol $min^{-1}$) by up to 500% in a dose dependent manner. This resulted in doxorubicin dependent reactive oxygen formation as indicated by stimulation of adrenochrome formation from adrenaline and greater than 90% inhibition of $MbFe(II)O_2$ conversion in the presence of the reactive oxygen scavengers superoxide dismutase plus catalase. Mitozantrone also stimulated $MbFe(II)O_2$ oxidation (up to 150%) but only at drug concentrations up to 50 μM, after which mitozantrone produced progressively less stimulation and above 200 μM actually inhibited the oxidation process. Furthermore mitozantrone (400 μM) totally inhibited doxorubicin dependent $MbFe(II)O_2$ oxidation. The results show that doxorubicin and to a lesser extent mitozantrone can interact with $MbFe(II)O_2$ to produce metmyoglobin. The reactive oxygen liberated by this process can directly oxidise $MbFe(II)O_2$ thereby exacerbating formation of metmyoglobin which cannot function as an oxygen storage protein. Furthermore reactive oxygen formation by this mechanism may contribute to other cardiotoxic consequences associated with doxorubicin and mitozantrone.

**Inherent adriamycin resistance in a murine tumour cell line. II: Influence of verapamil on adriamycin intracellular drug levels, metabolism and binding to DNA**

J. Cummings, R. Milroy, S. Merry, J.G. Morrison & S.B. Kaye

*Department of Medical Oncology, Glasgow, UK.*

The mechanism(s) by which verapamil (VPM) enhances the chemosensitivity of drug resistant tumour cells remains unclear. We have demonstrated that 6.6 μM VPM increases chemosensitivity of our mouse tumour line (MOG-XMT1) to Adriamycin (ADR) by up to 20-fold. Three possible mechanisms have been investigated in an attempt to explain this finding: increased intracellular ADR levels, changes in drug metabolism and changes in DNA binding affinity. For uptake studies intracellular drug and metabolite concentrations were measured by HPLC after exposure to 5 μg $ml^{-1}$ ADR. Competitive binding was performed on DNA isolated and purified from $10^8$ cells ($n = 3$). Apparent binding affinity constants (Kb(app)) and number of binding sites to DNA ($n$) were calculated by Scatchard analysis. In cell monolayers VPM at a concentration of 6.6 μM had no effect on intracellular ADR concentrations and no effect on qualitative or quantitative pathways of metabolism was observed. VPM at a concentration range of $1 \times 10^{-3}$ to $1 \times 10^{-6}$ M had no effect on the binding of ADR to isolated purified DNA (Kb(app) $2.2 \times 10^{-6}$ M, $n = 0.28$). We conclude that whilst VPM alters chemosensitivity in XMTI, it does so without altering drug accumulation, metabolism, or the capacity of ADR to bind to DNA. Our recent observations suggest that VPM may be acting by altering intracellular drug distribution, rather than overall concentration and further studies involving subcellular fractionation are in progress.

**Ethoxyquin induces preneoplastic changes in rat kidney**

M.M. Manson, J.A. Green & H.E. Driver

*MRC Toxicology Unit, Woodmansterne Road, Carshalton, Surrey SM5 4EF, UK.*

The antioxidant ethoxyquin (EQ) has been shown both to inhibit tumour formation, mainly in liver, and also to act as a tumour promoter in some extrahepatic tissues. At a dose (0.5% in the diet) which completely inhibited induction of hepatocellular preneoplastic foci by aflatoxin $B_1$, considerable renal damage was observed in 30 week old Fischer rats after 23 weeks of EQ treatment. Many of the pathological changes resembled those of chronic progressive glomerulonephrosis (CPGN), normally seen in very old animals, suggesting that EQ was accelerating the ageing process. Formation of haemosiderin and large amounts of lipofuscin were prominent in tubular cells. In addition to the changes of CPGN, EQ treated kidneys contained many hyperplastic tubules and small nodules of epithelial proliferation, often obliterating the lumen. Such changes are potentially preneoplastic and may be the precursors of adenocarcinomas. These hyperplastic tubules differed in a number of respects from those occasionally seen in CPGN and support for their preneoplastic nature was obtained by their basophilic staining with H and E, loss of GGT activity and PAS-positive brush borders, and strong staining for guanidinobenzoatase; hyperplastic tubules of CPGN do not stain for this tumour-associated enzyme. EQ-treated kidneys also showed induction for one or more isozymes of gamma glutamyl transpeptidase and glutathione S-transferase P (7-7 form), mainly in undamaged tubules of the outer cortex. Results suggested that EQ might be exerting a carcinogenic action in the kidney.

### DNA repair characteristics of Walker cells sensitive or resistant to difunctional agents

R.J. Knox & J.J. Roberts

*Department of Molecular Pharmacology, Institute of Cancer Research, Sutton, Surrey SM2 5PX, UK.*

The Walker 256 carcinoma cell (WS) is inherently sensitive only to difunctional agents such as *cis*-platin. Resistant Walker cells (WR) show comparable sensitivity as conventional cell lines. Both WS and WR cell lines are transfectable with pSV2gpt in suspension culture. Reaction of this plasmid with *cis*-platin or the monofunctional reacting Pt(Dien) prior to transfection caused a dose dependant decrease in the subsequent expression of XGPRT. At the same binding of Pt to the plasmid DNA a large difference in the effects of the monofunctional as opposed to the difunctional agent was apparent and this was a reflection of the relative cytotoxicities of these agents towards mammalian cells. However no significant difference was seen between WS or WR cells on the expression of pSV2gpt reacted with *cis*-platin even though WS cells are 20 fold more sensitive to this agent than WR cells. Based upon a knowledge of the proportions of the various adducts formed in DNA reacted with *cis*-platin, the lesion that inactivates expression of XGPRT was probably the intra-strand crosslink. Due to the size of the plasmid inter-strand crosslinks were not present in the plasmid DNA at these inactivating doses. Sensitivity to such relatively rare lesions is probably the basis of the defect in certain cells types such as the WS.

### Synthesis and structure of DNA containing O$^6$-methylguanine, O$^6$-ethylguanine and O$^4$-methylthymine

B.F.L. Li[1], P.F. Swann[1], M. Kalnik[2] & D.J. Patel[2]

[1]*Biochemistry Department, Middlesex Hospital Medical School, London W1P 7PN, UK and* [2]*Department of Biochemistry and Biophysics, College of Physicians and Surgeons, Columbia University, New York NY 10032, USA.*

An understanding of the recognition of modified DNA by DNA repair enzymes may throw light on some general aspects of protein DNA recognition. Self-complementary dodecadeoxynucleotides containing either one O$^6$-methylguanine (O$^6$-meG), one O$^6$-ethylguanine (O$^6$-etG), or one O$^4$-methylthymine (O$^4$-meT) residue have been synthesized by the phosphotriester approach in solution. They anneal to give double-stranded DNA in solution. The structure of double helices containing O$^6$-meG: cytosine (C), O$^6$-meG: thymine (T), O$^6$-etG: C, and G: O$^4$-meT base-pairs have been studied by NMR. The helices were right-handed with every nucleoside having the *anti* glycosidic torsion angle found in B-DNA. The modified bases stacked into the helix, and the alkyl groups were shown to be in the major groove of the DNA. NMR and optical melting profiles indicate that the helices are less stable than the non-alkylated parents but only in helices containing O$^6$-etG did the $^{31}$P spectrum give evidence of severe distortion of the phosphodiester backbone of the DNA. It has previously been believed that O$^6$-meG forms a stable base-pair with T and that O$^4$-meT forms one with G, and that in these base-pairs the imino protons of T and G are hydrogen bonded to N(1) or N(3) of the alkylated base. However, NMR shows that these protons are not involved in strong hydrogen bonds and casts doubt on the accepted structures for O$^4$-meT:G and O$^6$-alkylG:T mispairs.

### Repair of synthetic DNA: Kinetics of repair of oligodeoxy-nucleodites containing O$^6$-alkylguanine or O$^4$-methylthymine by *E.Coli* O$^6$-alkylguanine-DNA-alkyltransferase

R. Graves, B.F.L. Li & P.F. Swann

*Biochemistry Department, Middlesex Hospital Medical School, London W1P 7PN, UK.*

Self-complementary synthetic dodecadeoxynucleoties containing either one O$^6$-methlyguanine, one O$^6$-ethylguanine, or one O$^4$-methylthymine have been synthesized by the phosphotriester approach in solution or on solid phase supports. They have been used as substrates for DNA repair by the 18 kD O$^6$-alkylguanine-DNA-alkyltransferase of *E.coli* or its 39 kD precursor, the 39 kD product of the *ada* gene. The protein removed the alkyl cell group from all the alkylated bases. The reaction followed second-order chemical kinetics. The rates constants were:

O$^6$-methylguanine residues $k = 2.73 \times 10^7\ \text{M}^{-1}\ \text{sec}^{-1}$

O$^6$-ethylguanine residues $k = 2.58 \times 10^4\ \text{M}^{-1}\ \text{sec}^{-1}$

O$^4$-methylthymine residues $k = 2.52 \times 10^3\ \text{M}^{-1}\ \text{sec}^{-1}$

The rate of repair of O$^6$-methylguanine residues is close to the diffusion controlled limit ($7 \times 10^9\ \text{M}^{-1}\ \text{sec}^{-1}$) for second order chemical reactions. The availability of the defined sequence DNA-fragments containing modified bases promises to allow very sensitive and absolutely specific assay of DNA repair enzymes and to open the way to studies of, for example, mechanism of repair enzymes and the influence of DNA sequence on repair.

### High uptake of RSU 1069 into B16 melanoma

J. Walling[1], J. Deacon[1,2] & I. Stratford[1]

[1]*MRC Radiobiology Unit, Chilton, Didcot, Oxon and*
[2]*Institute of Cancer Research, Sutton, Surrey, UK.*

Pharmacokinetic studies of the hypoxic cell radiosensitizer RSU 1069 in B16 melanoma have revealed marked selective uptake. (Deacon *et al.*, *Int. J. Radiat. Oncol. Biol. Phys.*, **12,** 1087, 1986). This contrasts with low tumour to plasma ratios obtained in a previous study of RSU 1069 in the KHT sarcoma (Workman & Walton, *Int. J. Radiat. Oncol. Biol. Phys.*, **10,** 1307, 1984). We describe here experiments designed to determine the basis for the very large difference between these tumour systems for their uptake of RSU 1069.

The pharmacokinetics of RSU 1069 has been studied in C57 mice carrying the B16 or Lewis Lung tumours and in C3H mice bearing the KHT tumour. In each strain of mouse the maximum brain to plasma ratio was always less than 1.0, whereas the tumour/plasma ratio was 3.8, 0.5 and 0.4 for the B16, KHT and Lewis Lung tumours respectively. In order to test whether the differences observed have a cellular basis, drug uptake experiments were carried out *in vitro* using freshly exised tumour cells from B16, KHT, Lewis Lung, HX34 and HX118 tumours, the latter two are human amelanotic and melanotic melanoma xenografts respectively. Uptake of RSU 1069 into B16 melanoma cells *in vitro* is pH dependent. Values of the ratio of internal to external concentration (Ci/Ce) were 0.99, 1.35, 1.88 and 3.04 when cells were exposed at pH 5.4, 6.4, 7.4 and 8.4 respectively. In contrast RSU 1069 is *not* selectively taken up by any of the other tumour cell types *in vitro*, a Ci/Ce of unity being obtained over the pH range 5.4–8.4.

The results suggest the high tumour/plasma ratio to a characteristic of B16 cells alone and may indicate an intracellular pH difference (relative to the other cell types) that facilitates transport of the weak base, RSU 1069, into B16 cells.

**A model for the study of treatment response in human normal and tumour cells *in vitro***

C. Mothersill[1], C.B. Seymour[1], A. Cusack[1], A. O'Brien[2], M. Moriarty[1] & T.P. Hennessy[3]

[1]*Saint Luke's Hospital, Rathgar, Dublin 6;* [2]*Meath Hospital, Dublin 8 and* [3]*St James's Hospital, Dublin 8, Eire.*

Since all known chemotherapy and radiation treatments affect normal cells to a certain extent, the establishment of favourable differential sensitivities is fundamental to the success of treatment with a particular agent. This type of information can be gained by animal testing and using cultured cells but ultimately use of the agent in the patient is the only way to determine the response.

Our group has developed a model for testing the response of oesophageal explants from tumour and surrounding normal tissue in the same patient to chemotherapy and radiation, both singly and in combination. The test allows treatment combinations and time and order of administration of agents to the tissue to be accurately controlled. Cytotoxicity – determined by measuring the area of outgrowth from an explant two weeks after plating – is the most useful short-term endpoint although many others are possible.

Results on the differential cytotoxicity of bleomycin with and without radiation in adenocarcinoma of the oesophagus and surrounding normal tissue from the same patient indicate that low levels of bleomycin with or without radiation preferentially spare tumour cells while high levels, in combination with any dose of radiation tested, but not without radiation spare the normal cells and give a significantly high amount of relative tumour cell kill.

**Multicellular tumour spheroids as an *in vitro* model for antibody-targeted radiotherapy of cancer**

K.A. Walker, T. Murray, T.W. Wheldon, A. Gregor & I.M. Hann

*Glasgow Institute of Radiotherapeutics and Oncology, Belvidere Hospital, Glasgow G31 4PG and Radioisotope Dispensary, Glasgow Western Infirmary and Royal Hospital for Sick Children, Yorkhill, Glasgow, UK.*

Tumour spheroids have been used to provide *in vitro* simulation of antibody-targeted irradiation of micrometastases. A human neuroblastoma spheroid line (NB1-G) was exposed to $^{131}$I irradiation by incubation for 2 h with free $^{131}$I, $^{131}$I bound to non-specific protein and $^{131}$I conjugated to the anti-neuroectodermal monoclonal antibody UJ13A. Cells were then repeatedly washed to remove unbound activity and spheroid growth observed for up to 4 weeks. Spheroid response to $^{131}$I irradiation was assessed as growth delay and could be compared with the effects of known doses of external beam X-rays. The results demonstrated the relative effectiveness of $^{131}$I-UJ13A irradiation of neuroblastoma spheroids. A dose-response relationship was obtained by varying the concentration of the $^{131}$I-UJ13A conjugate. The tumour spheroid model provides a new approach to evaluation of antibody-targeted radiotherapy of microscopic tumours using different antibodies, antibody fragments and alternative radionuclides.

***In vitro* fibrin formation by ascitic and peritoneal fluids: A novel system for the study of fibrin-cell interactions**

A.P. Wilson

*Oncology Research Laboratory, Derby City Hospital, Uttoxeter Road, Derby DE3 3NE, UK.*

A large number of ascitic/peritoneal fluids have been tests for colony-stimulating-activity against tumour cells (TC) and transforming activity against mesothelial cells (MC) in a soft agar assay. A proportion of these fluids were found to induce varying degrees of spreading of MC in soft agar, leading to monolayer formation in some cases. Limited spreading of tumour cells also occurred. Addition of some of these fluids to monolayers of TC or MC resulted in visible formation of a fibrin mat which bound to the surface of MC but not to 4/4 ovarian tumour cell lines. Only one of these fluids had measurable levels of fibrinogen, though all had high levels of FDP. It was noteworthy that the majority of fluids which caused high-grade spreading came from patients who had not been exposed to chemotherapy. Fibrin mats in association with cells could be identified under phase contrast in the soft agar, and spreading of cells was presumably due to interaction between fibrin and the cell. It is suggested that the soft agar model provides a novel system for studying interactions between fibrin and normal cells or TC. The system also allows study of clonal heterogeneity in TC populations with respect to fibrin binding, as well as drug modulation of fibrin/cell interactions.

**Synthesis of oncogene anti-sense oligonucleotides and oligonucleotide analogues**

D.M. Tidd & H.M. Warenius

*University of Liverpool, CRC Department of Radiation Oncology, Clatterbridge Hospital, Bebington, Wirral L63 4JY, UK.*

Anti-sense oligodeoxynucleotides (20-mers) complementary to the untranslated sequence and codon 12 region of the human EJ/T24 Ha-*ras* gene have been synthesized and are being tested for effects on gene expression in *ras*-transformed NIH 3T3 cells. A system for post-synthesis end-on coupling of deprotected oligonucleotides to cell delivery molecules such as antibodies or polypeptides has been perfected. This comprises the coupling of the new agent 9-fluorenylmethoxycarbonyl-6-amino-1-hexanol-1-0-(2-chlorophenyl-1-benzotriazolyl)phosphate as the last step of phosphotriester synthesis. Following normal deprotection procedures a strongly nucleophilic free amino group is generated which participates readily in protein cross-linking reactions. The simplicity of the entire procedure was demonstrated by the synthesis of oligo(dT)$_{20}$-5′-0-(6-amino-1-hexanol) phosphate and its subsequent crosslinking to poly-L-lysine with dithio*bis* (succinimidyl propionate). We have also investigated the synthesis of oligonucleotide analogues containing modifications to the normal phosphodiester linkage. The novel analogue monomer, 5′-0-dimethoxytrityl-thymidine-3′-0-(4-anisidino)phosphate was synthesized and incorporated into oligonucleotides, however, the coupling efficiency was only 50% and therefore it could only be used realistically as a means of protecting 3′-termini from exonucleolytic degradation. Higher coupling efficiencies were achieved with fully protected 2′-deoxy-ribonucleoside-3′-0-(1-benzo-triazolyl)methylphosphonates (50–80%) and oncogene anti-sense oligonucleotides incorporating these entities are being synthesized in an attempt to influence gene expression from without.

### Synthesis and evaluation of substituted flavones as inhibitors of tyrosine protein kinase

B.D.M. Cunningham, M.D. Threadgill & J.A. Hickman

*CRC Experimental Chemotherapy Group, Pharmaceutical Sciences Institute, Aston University, Birmingham B4 7ET, UK.*

A number of proto- and viral oncogene products, which are considered to be involved in malignant transformation, are tyrosine specific protein kinases. Quercetin, 3,3′,4′,5,7-pentahydroxyflavone, a naturally occurring plant product, has been shown to inhibit the transforming gene product of the Rous Sarcoma virus, $pp60^{src}$ (Glossman *et al.*, *Naunyn Schmeidebergs Arch. Pharmacol.*, **317**, 100, 1981). $pp60^{src}$ is responsible for the transformation of certain cell types and its activity is required for the maintenance of the transformed phenotype. Since the inhibition of $pp60^{src}$ by quercetin is only one of its many activities, it is the aim of this project to synthesise rationally a series of analogues of quercetin that may show specific activity against $pp60^{src}$ and other associated tyrosine protein kinases. Compounds were synthesized by the acylation of 4,6-disubstituted 2-hydroxyacetophenones with 3,4-disubstituted benzoyl chlorides, followed by base catalyzed rearrangement and acid catalyzed ring closure. The results of cytotoxicity assays using Abelson-transformed NIH3T3 cells (ANN-1 cells) which express the Abelson tyrosine-protein kinase, show a range of I.C.$_{50}$s from 8 μM (3′4′-dihydroxyflavone) to 91 μM (quercetin).

### Tumour metastases can be augmented by drug treatment

P.J. Clarke & D. Tarin

*Nuffield Department of Pathology, (University of Oxford), John Radcliffe Hospital, Oxford OX3 9DU, UK.*

The cells within a tumour are known to be heterogeneous with regard to their metastatic capability. Environmental agents and drug treatments can both induce stable alterations in gene expression which are then reflected in heritable behavioural changes. To study the possible effect of such drugs on metastasis, the tumorigenicity and metastatic behaviour of a murine mammary tumour (P574) were studied before and after treatment *in vitro* with two drugs, 5-aza-2′-deoxycytidine (a hypomethylating agent), and tetradecanoylphorbolacetate (TPA), a known promotor of murine skin tumours.

P574 cells were treated *in vitro* with a single dose of either drug (5 μm 5-aza-2′-deoxycytidine, 10 ng ml$^{-1}$ TPA) for times varying from 1–24 h. After 24 h recovery, cells were inoculated into the fat pad of syngeneic mice and the effects on tumour growth and metastatic behaviour were documented.

P574 normally has a latent period of tumourigenicity of 12 weeks and is only weakly spontaneously metastatic. Both drugs accelerated the growth of fat pad tumours and significantly increased the numbers of pulmonary metastases. This increase in spontaneous tumour spread must reflect a heritable change induced by the drug treatments as many generations of cell division occur between tumour cell inoculation and metastases.

### Genotypic heterogeneity of human colorectal tumour cell populations

N.Y. Yassen[1], G. Jacob[2], C.W. Potter[1], A. Potter[3], A. Watmore[3] & R.C. Rees[1]

[1]*Department of Virology;* [2]*Department of Surgery, University of Sheffield Medical School and* [3]*Centre for Human Genetics, Langhill, Manchester Road, Sheffield 10, UK.*

Cytogenetic analysis of 6 long-term colon adenocarcinoma cell lines has been undertaken, and the results compared with those obtained from freshly derived short-term cultured (6 h) tumour cells. With established cell lines the cytogenetic characteristics included deletions, translocation events, addition and absence of chromosomes as well as the identification of a number of marker chromosomes, and other chromosomal abnormalities. A consistent feature of the cell lines was the prominent involvement of chromosomes numbers 5, 7, 11, 19 and 20, and the absence of the Y chromosome in tumours of male origin. In many of the commonly used SW cell lines up to five genotypically distinct subpopulations could be identified inferring considerable heterogeneity within any given cell line.

Analysis of freshly derived colon adenocarcinoma cells also showed genotypic heterogeneity within a given population, and the predominant population exhibited many of the characteristics associated with established cell lines: *viz.* additional chromosomes 5, 7, 11 and 20 and abnormalities in chromosomes 2, 3 and 19. It will be of considerable importance in further studies to define the phenotypic and biological characteristics associated with apparent genotypic changes.

### Modulation of type IV collagenase and plasminogen activation secretion in hamster fibrosarcoma sublines and clones possessing differing metastatic potential

R.C. Rees, D.M. Teale & I.A. Khidair

*Department of Virology, University of Sheffield Medical School, Beech Hill Road, Sheffield S10 2RX, UK.*

The effect of basement membrane components (laminin, fibronectin and type IV collagen) on type IV collagenase and plasminogen activator secretion was investigated in a primary HSV-2 induced hamster fibrosarcoma, and its *in vivo* derived sublines and *in vitro* derived clones of previously defined high or low metastatic potential. Fibronectin and type IV collagen were ineffective in influencing the expression of either type IV collagenase or plasminogen activator activity. Laminin however, at concentrations of 1–10 μg ml$^{-1}$, when added to serum-free cultured supernatants, increased the release of type IV collagenase by up to 100% for the parental cell line. Three highly metastatic sublines (two *in vivo* derived, and one *in vitro* cloned) showed increases of up to 300% in type IV collagenase secretion upon laminin stimulation; in contrast non-metastatic sublines (two *in vivo* and one *in vitro* cloned) showed no increase in type IV collagenase activity in response to laminin. Plasminogen activator released from either the parental cell line or its metastatic sublines and clones was unaffected by the addition of laminin. These studies infer a role for laminin, via its interaction with the laminin receptor site on tumour cells, leading to an increase in type IV collagenase secretion; this process may contribute to the process of tumour metastases and invasion of tumour cells through basement membrane matrices.

### Mutant *ras* genes influence early stages in spontaneous metastasis

S.A. Eccles[1], H. Purvies[1]* & C.J. Marshall[2]

[1]*Institute of Cancer Research, Sutton, Surrey and* [2]*Chester Beatty Laboratories, London, UK.*

Introduction of an activated c-Ha-*ras*-1 gene by transfection with the pSV2-neo vector has been shown to enhance the spontaneous metastatic capacity of syngeneic mouse mammary carcinoma cells from $<10\%$ incidence to $>90\%$. (Vousden *et al., Int. J. Cancer,* **37,** 425, 1986). Control transfectants (pSV2-neo alone) showed a slightly increased tendency to metastasize which was rapidly lost during *in vitro* or *in vivo* passage, whereas the *ras*-induced metastatic phenotype appeared stable. Various parameters are being investigated with the aim of elucidating the mechanism of action of this oncogene. *Ras* mutants with a serine residue at codon 12 were capable of inducing metastasis of transfected cells, indicating that the glycine→valine mutation is not unique in this capacity. The *ras* transfectants were found not to differ significantly from the parental or pSV2-neo controls in terms of: growth rate, immunogenicity, ploidy, collagenase activity, size distribution of cell surface glycopeptides, or lung colonisation potential. We infer from these data that the *ras* oncogene influences as yet unidentified events early in the metastatic process (e.g. cell detachment, invasion, intravasation), and these aspects are now being investigated.

### Observations on the fluorescent staining of melanocytes in benign naevi and malignant melanoma

F.S. Steven, U. Suresh, T. Wong & M.M. Griffin

*Department of Biochemistry and Molecular Biology, University of Manchester, Manchester M13 9PT and Department of Histopathology, Withington Hospital, Manchester M20 8LR, UK.*

Guanidinobenzoatase is a cell surface, trypsin-like, protease capable of degrading fibronectin. Cells possessing this enzyme exhibit cell surface fluorescence when treated with a competitive inhibitor such as 9-amino-acridine. Melanocytes in routine wax embedded skin sections could be classified as benign naevus cells or malignant melanoma cells according to their ability to bind 9-aminoacridine. This binding ability was controlled by the presence in benign naevus cells of an inhibitor whilst in the malignant melanoma cells the inhibitor was absent.

The inhibitor could be displaced experimentally and exchanged with an inhibitor of guanidinobenzoatase obtained from fresh liver homogenates. We suggest guanidinobenzoatase and its inhibitor may have roles in the control of cell migration.

### A histopathological and DNA cytophotometric study of benign and malignant oesophageal tissue cultured *in vitro*

A. O'Brien[1], A. Cusack[2], C. Mothersill[2], C. Seymour[2], M. Moriarty[2] & T.P.J. Hennessy[1]

[1]*University Department of Surgery, St James's Hospital, Dublin 8 and* [2]*Department of Radiobiology, St Luke's Hospital, Rathgar, Dublin 6, Eire.*

In an attempt to look at the problem of growing human oesophageal mucosal cells in culture our group have correlated the histopathological findings with observed growth and with DNA cytophotometric analysis. In 6 cases of surgical resection of adenocarcinoma (3) or squamous carcinoma (3) of the oesophagus, specimens of tumour and macroscopically normal mucosa were cultured *in vitro* for 28 days. The extent of growth was correlated with results from sample biopsies submitted for histopathological examination. DNA cytophotometry was also studied using a single cell scanning microdensitometer. Biopsies of oesophageal and gastric mucosa from endoscopically normal patients were used to determine the normal diploid DNA content.

The study showed no correlation between normal or tumour cell growth *in vitro* and histological tumour type, stage, grade, ploidy value dysplasia, carcinoma *in situ* or presence or absence of Barret's mucosa. Tumour tissue in general grew significantly less than normal tissue ($P<0.01$) and there was a significantly greater amount of growth in samples of tumour tissue from patients with regional nodal metastasis ($P<0.01$). However, when growth of macroscopically normal mucosa was correlated with the presence or absence of regional node metastases, the presence of positive nodes was associated with significantly less normal mucosal cell growth *in vitro* ($P<0.05$). The association of tumour cell growth *in vitro* with metastatic spread might be expected but the reason for reduced normal growth in these circumstances is unclear. The results indicate that apart from metastatic status, the histopathology of the tumour has no relevance to the subsequent growth of either normal or tumour cells *in vitro*.

### Dietary calcium supplementation reduces carcinogenesis and crypt cell production rates in normal and adapting colonic epithelium

G.V.N. Appleton, P.W. Davies, J.B. Bristol & R.C.N. Williamson

*University Department of Surgery, Bristol Royal Infirmary, Bristol BS2 8HW, UK.*

Small bowel resection and intrarectal administration of sodium deoxycholate each stimulate cell proliferation and promote carcinogenesis in the large intestine; oral supplements of calcium reduce the mitogenic effect of bile acids on colorectal mucosa. Potential suppression of intestinal adaptation and carcinogenesis by intraluminal calcium was tested in 120 male Sprague–Dawley rats weighing $186\pm9$ g. Rats were randomised to receive azoxymethane s.c. $15\,mg\,kg^{-1}\,wk^{-1}$ for 6 wk or vehicle, followed by 80% mid small bowel resection or transection with reanastomosis. Half the animals in each group received supplemental calcium in the drinking water (calcium lactate $24\,g\,l^{-1}$). Crypt cell production rate (CCPR) in descending colon was determined 7 wk postoperatively in vehicle-treated rats; in the remainder colonic tumour yield was assessed at 26 wk. Among rats with transection, calcium supplements reduced colonic CCPR by 26% from $4.49\pm0.33$ to $3.32\pm0.40$ cells per crypt/h ($P<0.05$) and more than halved tumour yield from 4.3 to 1.8 tumours per rat ($P=0.0007$). Jejunoileal resection increased both CCPR (by 51–61%: $P<0.001$) and tumour yield (by 65–105%: $P<0.005$), but again calcium lowered CCPR by 31% ($7.23\pm0.44$ *vs.* $4.98\pm0.70$; $P<0.02$) and tumour yield by 46% (6.9 *vs.* 3.7; $P=0.0006$). Increased dietary levels of calcium diminish both adaptive and neoplastic growth in the colon, and calcium also blunts the co-carcinogenic stimulus of massive enterectomy.

**Survival of multiple cycles of DNA synthesis detected in single mammalian cells using cytochalasin B. A flow cytometric method**

J.B. Court, C. Burn & J.L. Moore

*Radiation Sciences Laboratory, Velindre Hospital, Cardiff CF4 7XL, Wales, UK.*

We have developed an assay for Chinese hamster ovary cell viability that is based on the ability to multinucleate in the presence of the fungal metabolite cytochalasin B (CB) following exposure to radiation or to cytotoxic drugs. The degree of multinucleation achieved can be assessed after a given incubation period in CB either by a count of nuclei/cell or by measurement of the cellular DNA content. The method is intended to permit estimation of the survival of DNA synthesis in situations where cells cannot be fully monodispersed. Using a flow-cytometric method to find the cellular DNA content and to enumerate the absolute number of cells per culture, we have investigated the effect of cell density and glucose concentration on the cellular DNA content achieved after 50 h incubation in the presence of CB. Above densities of $\sim$80,000 cells $cm^2$ the proportion of seeded cells accumulating at least 3 times the DNA content of untreated G1 cells is reduced, though the effect on the measured survival following 20 Gy irradiation is small. The proportion of cells exceeding a DNA content of $3 \times G1$ is sensitive to the concentration of glucose in the incubation medium. We postulate that the reduction in the cellular surface area/volume ratio that may occur as cultures become dense limits the acquisition of medium energy sources.

We have also investigated the shape of survival curves following irradiation or drug exposure. The survival of cells able to exceed a DNA content of $3 \times G1$ after 50 h in CB seems to decrease exponentially with dose. Generally, survival is markedly greater than the corresponding clonogenic survival.